# Identification of Peptides and Their GPCRs in the Peppermint Shrimp *Lysmata vittata*, a Protandric Simultaneous Hermaphrodite Species

**DOI:** 10.3389/fendo.2020.00226

**Published:** 2020-04-30

**Authors:** Chenchang Bao, Fang Liu, Yanan Yang, Qi Lin, Haihui Ye

**Affiliations:** ^1^School of Marine Science, Ningbo University, Ningbo, China; ^2^College of Ocean and Earth Sciences, Xiamen University, Xiamen, China; ^3^Fisheries Research Institute of Fujian, Xiamen, China

**Keywords:** peptides, GPCRs, bioinformatics, protandric simultaneous hermaphrodite, *Lysmata vittata*

## Abstract

Peptide hormones commonly binding with G-protein coupled receptors (GPCRs) achieve their function in reproduction. The peppermint shrimp *Lysmata vittata* popular in marine ornamental trade and is known to display protandric simultaneous hermaphrodite (PSH). Knowledge on reproductive biology of this commercial species is critical for resources management and aquaculture. This study employed Illumina sequencing and bioinformatics analysis to identify peptides and their candidate GPCRs from male phase (MP) and euhermaphrodite phase (EP) of *L. vittata*. A total of 61 peptide and 40 peptide GPCR transcripts derive from 44 peptide families and 13 peptide GPCR families were identified, respectively. Among them, insulin-like androgenic gland hormone and crustacean female sex hormone have two unique mature peptides, respectively, and their transcripts showed higher expression levels in MP than EP, which suggest that these sex differentiation hormones might be involved in sexual characters than spermatogenesis or vitellogenesis. Overall, the first study on identification of peptides and their GPCRs in the genus *Lysmata* extends our knowledge of peptidergic signaling in PSH species, and provides an important basis for development of aquaculture strategies.

## Introduction

Peptide hormones play an important role in crustaceans reproduction. Crustacean hyperglycemic hormone superfamily (CHHs) are the typical crustacean peptide hormones. They are classified into type-I [CHH-type, comprised of crustacean hyperglycemic hormone (CHH) and ion transport peptide (ITP)] and type-II [MIH-type, comprised of molt-inhibiting hormone (MIH), mandibular organ-inhibiting hormone (MOIH), and vitellogenesis/gonad-inhibiting hormone (VIH/GIH)] peptides, involved in inhibiting ecdysteroid, methyl farnesoate, and vitellogenin synthesis (1). Insulin-like androgenic gland hormone (IAG) secreted by a crustacean male-specific androgenic gland (AG) is regarded as a peptidergic hormone and regulates male sex differentiation. IAG silencing in the giant prawn *Macrobrachium rosenbergii* (2) and red claw crayfish *Cherax quadricarinatus* (3) resulted in masculinization arrest and functional sex reversal, leading to the production of monosex populations (4). Moreover, IAG is not the sole sex differentiation hormone in crustaceans, and crustacean female sex hormone (CFSH), a specific hormone that plays critical role in female reproductive phenotypes was recently isolated from the eyestalk of female blue crab *Callinectes sapidus* (5). Silencing CFSH impairs the mating and maternal care structures of females, such as absent or misplaced gonopores, sharper abdomens, as well as shorter and fewer setae on pleopods (5). Recent studies have found that several peptide hormones are also involved in crustacean reproduction. This includes the pigment-dispersing hormone (PDH) (6), neuroparsin (7), red pigment concentrating hormone (RPCH) (8), neuropeptide F (NPF) (9), short neuropeptide F (sNPF) (10), and allatostatin (AST) (11).

The colorful *Lysmata* shrimp collected from their natural environments is popular in marine ornamental trade, which are collected from their natural environments (12). Considering its growing demand in marine ornamental industry, it is important to study its reproductive biology for natural resources conservation and development of breeding techniques. Different from the gonochoristic reproductive system in most crustaceans, protandric simultaneous hermaphrodite (PSH) has been confirmed in all known species in genus *Lysmata* (13, 14). In PSH, the shrimp first develops as male (male phase, MP), and later become simultaneous hermaphrodite (euhermaphrodite phase, EP) which simultaneously produces sperms and eggs (13–15).

The peppermint shrimp *Lysmata vittata* is a small red-striped species, found in the coast of China, Japan, Philippines, Indonesia, and Australia (16–19). This species was also reported to have invaded the Atlantic Ocean, New Zealand and Brazil (12, 20, 21). Based on its histological features, four gonadal development stages (Stage I to IV) were defined for *L. vittata*. Among the four stages, stage I to III were defined as the male phase, during which testicular part of the gonads gradually develops and matures but the ovarian part remains immature. Stage IV was identified as euhermaphrodite phase, where both the testicular part and the ovarian part of the gonad mature (15). To date, study on the reproductive biology of *L. vittata* has mainly focused on: (1) the reproductive cycle of laboratory-reared (22), (2) ontogenetic development of gonads, and (3) external sexual characteristics (15). However, the reproduction molecular mechanisms, especially the information about peptide hormones is still unclear. Using Illumina sequencing and bioinformatics analysis, this paper tries to identify the peptide repertoire and their GPCRs in *L. vittata*, highlighting two sex differentiation peptide hormones, IAG and CFSH. This is useful for understanding specific PSH reproductive regulatory mechanism, and for supporting aquaculture to meet the emerging demand.

## Materials and Methods

### Animals

*L. vittata* shrimps were cultured in the aquarium at Fisheries Research Institute of Fujian, Xiamen, China. Prior to dissections, the shrimps were anesthetized on ice for 5 min. Our study does not involve endangered or protected species.

### Illumina Sequencing

Total RNA from mixed tissues of MP carapace (mixture of 5 individuals, body weight 86–100 mg, stage I and stage II) and EP carapace (mixture of 3 individuals, body weight 260–300 mg, stage IV), was extracted using Trizol Reagent (Invitrogen), followed by Illumina sequencing. Briefly, mRNA with poly (A) was isolated from total RNA using Oligo (dT) beads (Invitrogen). The mRNA was broken into short fragments (about 200 bp) using fragmentation buffer. These fragments were used as templates to synthesize the first-strand cDNA with random hexamers, after which a second-strand cDNA was synthesized. Adaptors were ligated onto the second-strand cDNA following by Illumina Hiseq sequencing (HiSeq 4000 SBS Kit (300 cycles), Illumina). The raw reads were quality controlled using Trimmomatic to generate clean reads, before performing *de novo* assembly through Trinity (v2.5.1). All clean reads were aligned with Bowtie2 (v2.3.4), followed by joint abundance estimation and RSEM to calculate transcripts per million (TPM) values.

### Bioinformatics Analysis

Peptide and GPCR sequences were collected from the shrimp *de novo* assembly. To identify peptide, we used the well-established workflow (23). Signal peptide of the peptide precursors were predicted using SignalP 4.1 (http://www.cbs.dtu.dk/services/SignalP/). Prohormone cleavage sites prediction based on the standards were defined by Veenstra (24) and the peptide structures were predicted based on the established propeptide processing schemes (25–27). GPCRs identification was performed as our previous study (28). Deorphanized peptide GPCRs from insects and reported peptide GPCRs from crustaceans were used as reference sequences (28–37). A phylogenetic tree was built with related sequences of these GPCRs and *L. vittata* GPCRs transmembrane domains. Multiple sequence alignment was performed with ClustalX and the conserved sequence motifs were highlighted by LaTEX TexShade (38). The phylogenetic analysis was calculated using PhyML (SeaView software) (39) and the resultant phylogenetic tree was visualized with Figtree v1.4.3 and Photoshop CS 6.

## Results

The mRNA-sequencing and *de novo* assembly data are shown in Table S1. The assembled transcripts (*N* = 71,009) had a total size of 65,718,743 bp, an average size of 925.5 bp and N50 assembled transcripts with 1687 bp long. Using transcriptome mining, a total of 61 peptide and 40 peptide GPCR (34 belonging to A-family GPCRs (Lv-GPCR-A) and 6 belonging to B-family GPCRs [Lv-GPCR-B)] transcripts were predicted in *L. vittata*. These peptides included: adipokinetic hormone-corazonin-like peptide (ACP), agatoxin-like peptide, allatostatin-A (AST-A), AST-B, AST-C, AST-CC, AST-CCC, bursicon hormone, calcitonin, calcitonin-like diuretic hormone (DH31), CCHamide, CRF-like DH44, crustacean cardioactive peptide (CCAP), crustacean female sex hormone (CFSH), crustacean hyperglycemic hormone (CHH), molt-inhibiting hormone/gonad-inhibiting hormone (MIH/GIH), CHH-MIH-like peptide, ecdysis triggering hormone (ETH), eclosion hormone (EH), EFLamide, FLRFamide, glycoprotein-A2 (GPA2), glycoprotein-B5 (GPB5), Hyrg, insulin-like androgenic gland hormone (IAG), kinin, myosuppressin, natalisin, neuroparsin, neuropeptide F (NPF), orcokinin, pigment-dispersing hormone (PDH), proctolin, pyrokinin, red pigment-concentrating hormone (RPCH), RYamide, short neuropeptide F (sNPF), SIFamide, sulfakinin, tachykinin, terminal ampullae peptide (TAP), trissin and vasopressin. The peptide and GPCR transcripts source and their expression levels (TPM values) are summarized in Supplementary files (Supplementary File 1 and Table S1). Most of the peptide transcripts TPM values (51 out of 61 transcripts) in MP are higher than those in EP (Table S1). Although the expression levels of GPCRs are generally lower than peptides, their expression patterns are similar, i.e., GPCR transcripts TPM values (33 out of 40 transcripts) in MP are higher than those in EP (Table S1).

### Adipokinetic Hormone-Corazonin-Like Peptide (ACP)

Two transcripts putatively encoded complete ACP precursors of 104 and 99 amino acids (aa), respectively (Supplementary File 1). These precursors have the same mature peptide, pQITFSRSWVPQamide, a highly conserved decapod ACP peptide [e.g., (40–42)].

### Agatoxin-Like Peptide

One transcript was identified to encode agatoxin-like peptide precursor of 111aa (Supplementary File 1). From this precursor, a 21aa signal peptide and three distinct peptides were predicted, one of which, WRSCIPRGGSCTHRPKSCCNSSSCRCNLWGTNCRCQRMGLFQQLamide, shows 8 cysteine residues and amidated C-terminus associated with insect and decapod agatoxin-like peptides (43). Similarly, apart from toxic purposes, agatoxin-like peptide were identified in the neuroendocrine system of honey bee *Apis mellifera* and other insects (44).

### Allatostatin (AST)

**Figure 1:**
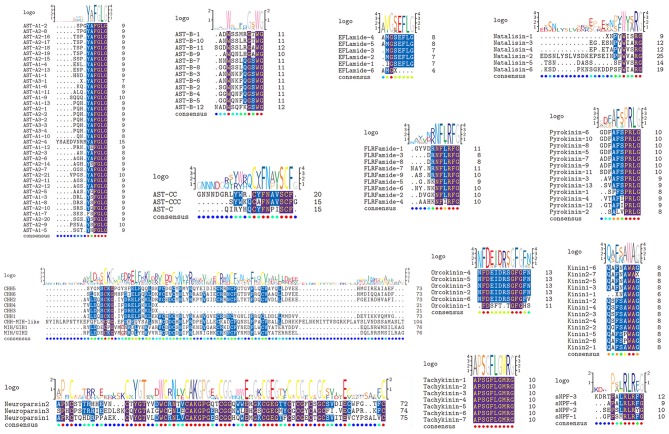
Identification and characterization of mature peptides in *Lysmata vittata*. Schematic showing the mature peptides of ASTs, CHHs, EFLamide, FLRFamide, Kinin, Natalisin, Neuroparsin, Orcokinin, Pyrokinin, sNPF, and Tachykinin identified in *Lysmata vittata*. Logo is shown above alignments, where the height of each letter is proportional to the observed frequency of the corresponding amino acid in the alignment column.

Three transcripts, a complete (AST-A2), a C-terminus (AST-A2) and a middle region (AST-A3) transcript with 334, 332 and 77aa, respectively that encode AST-A precursors were identified (Supplementary File 1). A total of 38 predicted peptides containing FGLamide were highly conserved motif derived from these precursors (Figure 1). Apart from SKSFSFGLamide, the rest of these peptides possess a conserved C-terminal motif YXFGLamide, e.g., SPGYAFGLamide, the signature of AST-A family (45). One transcript putatively encoded the complete AST-B precursor with 350aa. This precursor has 12 predicted mature peptides with a XWXXXXGXWamide conserved motif (Figure 1), e.g., ADWSSMRGTWGamide sequence, the signature of AST-B family (45). Three transcripts, two C-terminus partial regions (AST-C, AST-CC) and one full-length protein (AST-CCC), with 137, 192 and 108 aa, respectively that encode AST-C precursors were identified from the transcriptome assembly (Supplementary File 1). Each precursor possessed a predicted peptide with conserved motif XCXFNXXSCFX (Figure 1), i.e., pQIRYHQCYFNPISCF from AST-C, GNNNDGRLYWRCYFNAVSCF from AST-CC, and SYWKQCAFNAVSCFamide from AST-CCC (a disulfide bridge between cysteine residues in each peptide), previously reported decapod AST-C isoforms signature (46–49).

### Bursicon Hormone

The heterodimeric peptide bursicon hormone alpha and beta subunit sequences were identified from the transcriptome assembly, encoding bursicon hormone alpha and bursicon hormone beta precursor of 148 and 136aa, respectively (Supplementary File 1). Both of these precursors start with a predicted signal peptide, followed by adjacent mature peptide with 11 cysteine residues.

### Calcitonin

A single calcitonin transcript encodinng 164aa precursor was identified (Supplementary File 1). It comprised of 21aa signal peptide and three distinct peptides, one of which, TCYINAGLSHGCDYKDLVGAMAEKNYWDSLNSPamide (a disulfide bridge between two cysteine residues) is identical in structure to calcitonin from several decapod species, e.g., *M. rosenbergii*, the American lobster *Homarus americanus*, the crayfish *Procambarus clarkii* (43).

### Calcitonin-Like Diuretic Hormone (DH31)

The predicted DH31 precursor was composed of 142aa with 23aa signal peptide and three distinct peptides (Supplementary File 1), where one of the peptides, GLDLGLGRGFSGSQAAKHLMGLAAANFAGGPamide, possesses conserved motif XXDXGLXRGXSGXXXAKXXXXXXXANXXXGPamide, the signature of DH31 family. Similar to calcitonin, *L. vittata* DH31 is identical in structure to DH31 from several decapod species, e.g., *H. americanus, M. rosenbergii* (43, 46, 50).

### CCHamide

A single transcript encoding CCHamide precursor was identified (Supplementary File 1), starting with a 23aa signal peptide, followed by a C-terminal amidated peptide, i.e., VPKGGCLNYGHSCLGAHamide (a disulfide bridge between two cysteine residues), exhibiting conserved motif XCXXW/Y/FGXXCXGXHamide of CCHamide (51).

### CRF-Like DH44

A single transcript was identified to encode incomplete CRF-like DH44 precursor with 230aa (Supplementary File 1). This precursor has a 45aa mature peptide, i.e., NSGLSLSIDASMKVLREALYLEMARKKQRQQMLRARHNQALLTTIamide, is similar to the previously described *M. rosenbergii* DH44 isoform, SSGLSLSIDASMKVLREALYLEMARKKQRQQMQRARHNQELLTSIamide (43, 50).

### Crustacean Cardioactive Peptide (CCAP)

A single transcript was identified to encode CCAP precursor of 141aa (Supplementary File 1). A 30aa signal peptide and five distinct mature peptides were predicted from CCAP precursor, one of which, PFCNAFTGCamide (a disulfide bridge between two cysteine residues), is identical to previously described authentic CCAP, a conserved arthropod peptide [e.g., (25, 45)].

### Crustacean Female Sex Hormone (CFSH)

Two transcripts were identified to encode CFSH precursors representing the partial N-terminus (CFSH1a) and complete protein (CFSH1b) of 208 and 229aa, respectively. A 35aa signal peptide, a CFSH precursor-related peptide and a part of the 136aa mature peptide with 6 cysteines were predicted from CFSH1a. Similarly, the CFSH1b has a 33aa signal peptide, a CFSH precursor-related peptide and the 163aa mature peptide with 8 cysteines (Supplementary File 1). Both mature peptides (except two cysteine residues lacking in CFSH1a) showed similar cysteine residues with the other decapod CFSHs (43). The phylogenetic tree revealed that two *L. vittata* CFSHs clustered with previously described decapod CFSH1 isoforms (43), and were analogous to *M. rosenbergii* CFSH1a and CFSH1b respectively (Figure S1). Therefore, we arbitrarily named them as *L. vittata* CFSH1a and *L. vittata* CFSH1b in our study.

### Crustacean Hyperglycemic Hormone Superfamily (CHHs)

**Figure 2:**
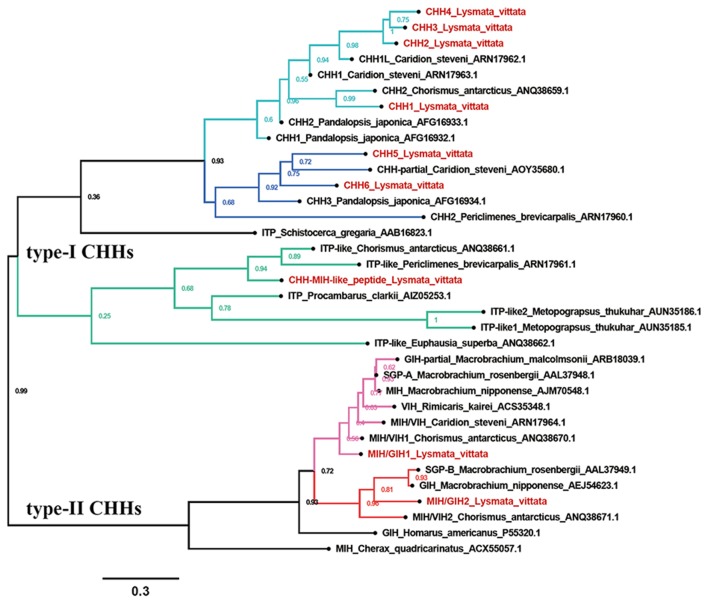
Phylogeny of CHHs. Cladogram of CHHs showing connections in the clustermap of CHHs.

Nine CHHs transcripts were identified from the transcriptome assembly. Phylogenetic analysis showed that the CHHs formed two major clades: type-I CHHs and type-II CHHs. Overall, type-I CHHs clade contained three subclades: the CHHs ortholog containing CHH1-4, the CHHs ortholog containing CHH5 and CHH6, and the CHH-MIH-like peptide ortholog. In type-II CHHs clade, MIH/GIH2, the oriental river prawn *Macrobrachium nipponense* GIH, *M. rosenbergii* SGP-B and the Antarctic shrimp *Chorismus antarcticus* MIH/VIH formed a subgroup, separate from the *M. nipponense* MIH ortholog containing MIH/GIH1 (Figure 2).

### Crustacean Hyperglycemic Hormone (CHH)

**Figure 3:**
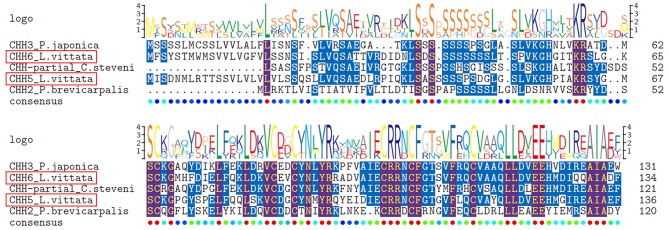
Comparative sequence alignment of CHHs with “AIAXX” in different Caridea. Comparative sequence alignment of CHHs with “AIAXX” in *Lysmata vittata* with *Pandalopsis japonica, Caridion steveni* and *Periclimenes brevicarpalis*. Sequence logo is shown above alignments.

Six transcripts were identified to encode four complete (CHH1, CHH2, CHH5, and CHH6), one N-terminus (CHH4) and one middle region (CHH3) CHH precursors. Altogether, these sequences have a CHH precursor-related peptide (CHH-PRP) between signal peptide and mature peptide. CHH1 precursor has a 26aa signal peptide, a 37aa CHH-PRP and a 72aa mature peptide with amidated C-terminus and 6 cysteines. CHH2 precursor has a 27aa signal peptide, a 43aa CHH-PRP and a 73aa mature peptide with 6 cysteines. CHH3 precursor has a partial signal peptide, a 44aa CHH-PRP and a part of mature peptide with 1 cysteine. CHH4 precursor has a 21aa signal peptide, a 34aa CHH-PRP and a partial mature peptide (20aa) with 1 cysteine. CHH1-4 were shown to be highly conserved sequences at the N-terminus of their mature peptides (Figure 1). CHH5 precursor has a 29aa signal peptide, a 32aa CHH-PRP and a 73aa mature peptide with 6 cysteines. CHH6 precursor has a 28aa signal peptide, a 31aa CHH-PRP and a 73aa mature peptide with 6 cysteines (Supplementary File 1 and Figure 1). Different from CHH1-4, CHH5, and CHH6 exhibited high sequence similarity with 3 Caridea CHHs, with a conserved C-terminus: AIAXX (Figure 3).

### Molt-Inhibiting Hormone/Gonad-Inhibiting Hormone (MIH/GIH)

Two transcripts were identified to encode MIH/GIH precursors with 110 and 112aa, respectively. They lack CHH-PRP and have an additional specific glycine in position 12 of the mature peptide (Gly_12_). The precursor MIH/GIH1 has a 32aa signal peptide and a 75aa mature peptide with amidated C-terminus. The precursor MIH/GIH2 has 34aa signal peptide and 75aa mature peptide with amidated C-terminus (Supplementary File 1 and Figure 1).

### CHH-MIH-Like Peptide

One transcript was identified to encode 133aa CHH-MIH-like peptide precursor with a 29aa signal peptide and a 104aa mature peptide with 6 cysteines (Supplementary File 1 and Figure 1). This peptide defies the rules of CHH superfamily, i.e., lacks the CHH-PRP and Gly_12_, and it is closer to the type-I CHHs than to type-II CHHs cluster (Figure 2). It exhibits similar characteristics of CHH-MIH-like peptide from several decapod species such as, *P. clarkii, E. sinensis* and the white shrimp *Litopenaeus vannamei* (43).

### Ecdysis Triggering Hormone (ETH)

One transcript was identified from the transcriptome assembly to encode ETH precursor of 135aa (Supplementary File 1). A 19aa signal peptide and two distinct mature peptides were predicted from the ETH precursor, one of which, i.e., DAGHFFAETPKHLPRIamide, is identical in structure to decapod ETH isoforms [e.g., (43)].

### Eclosion Hormone (EH)

One transcript was identified to encode EH precursor of 82aa, starting with a 26aa signal peptide, followed by a 52aa mature peptides with 6 cysteines, i.e., ASITSMCIRNCGQCKEMYGDYFHGQACAESCIMTQGVSIPDCNNPATFNRFL. This is identical in structure to EH from several decapod species, e.g., *M. rosenbergii, P. clarkii* (37, 43, 50).

### EFLamide

One transcript was identified to encode the N-terminus EFLamide precursor of 210aa, starting with a 21aa signal peptide (Supplementary File 1). Eleven peptides were predicted from this precursor, five possessing the conserved motif GSEFLamide (Figure 1), e.g., IGSEFLamide, AMGSEFLamide, the signature of EFLamide (or called GSEFLamide) family [e.g., (43, 52)]. One of these predicted peptide with incomplete sequence, AMG, was predicted as the N-terminus of EFLamide isoform.

### FLRFamide (FMRFamide)

One transcript was identified to encode FLRFamide precursor with 335aa, starting with a 19aa signal peptide (Supplementary File 1). Seventeen mature peptides were predicted from FLRFamide precursor, nine of which, are 7-10aa in length with conserved motif NFL/IRFamide (Figure 1), e.g., GYVDRNFLRFamide, and AAHKNFIRFamide, the signature of FLRFamide family [e.g., (45)].

### Glycoprotein-A2 (GPA2)

The predicted GPA2 precursor has 18aa signal peptide and part of mature peptide with 4 cysteines (Supplementary File 1), i.e., FQHAWQTPGCHKVGHTRKISIPECVEFDITTNACRGYCE, which shows highly conserved sequence like previously described decapod GPA2 isoforms, e.g., it is 92% identical in protein sequence to *C. quadricarinatus* GPA2 isoform (53).

### Glycoprotein-B5 (GPB5)

One transcript was identified to encode GPB5 precursor starting with a signal peptide with no N-terminus, followed by a 125aa C-terminal amidated mature peptide with 10 cysteines (Supplementary File 1). It shows a major sequence similarity to previously described decapod GPA5 isoforms, e.g., it is 87% identical/94% positives to GPA5 of *L. vannamei* (43).

### Hyrg

Two transcripts were identified to encode Hyrg precursors with 60aa and 63aa. Each precursor is composed of a signal peptide and a mature peptide. The peptides, i.e., YPEPAVIVDGRPNMIPDGYIQAPRFHYRGFQKPIPKYDWS from Hyrg1, LPEAAVIVEGRPNRAPDDGYVQAAPPRFHYRGFQKFVPKYDWS from Hyrg2, possess conserved motif RFHYRGF, the signature of decapod Hyrg isoforms (43).

### Insulin-Like Androgenic Gland Hormone (IAG)

**Figure 4:**
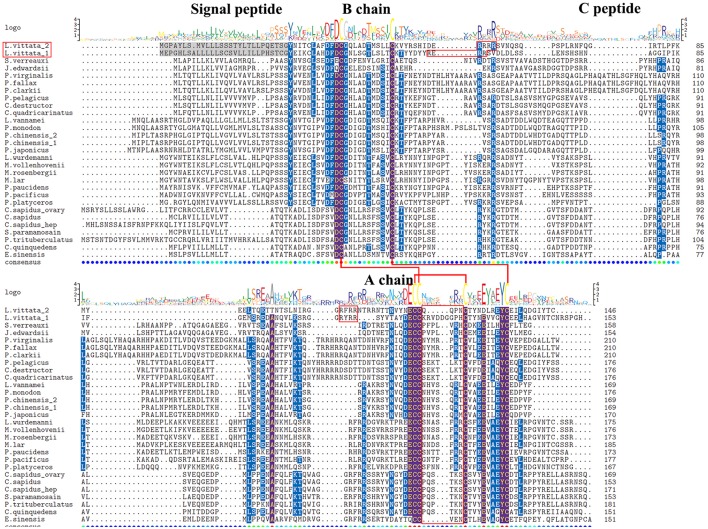
Comparative sequence alignment of IAG in different decapod. Comparative sequence alignment of IAG precursors in *Lysmata vittata* with *Sagmariasus verreauxi, Jasus edwardsii, Procambarus virginalis, Procambarus fallax, Procambarus clarkii, Portunus pelagicus, Cherax destructor, Cherax quadricarinatus, Litopenaeus vannamei, Penaeus monodon, Penaeus chinensis, Penaeus japonicus, Lysmata wurdemanni, Macrobrachium vollenhovenii, Macrobrachium rosenbergii, Macrobrachium lar, Palaemon paucidens, Palaemon pacificus, Pandalus platyceros, Callinectes sapidus, Scylla paramamosain, Portunus trituberculatus, Chaceon quinquedens*, and *Eriocheir sinensis*. Sequence logo is shown above alignments. Six conserved cysteines are highlighted in the sequence logo shown above alignments and the putative disulfide bridges are drawn with red line.

Two transcripts were identified to encode IAG precursors with 146aa and 153aa (Supplementary File 1). IAG1 precursor has a 28aa signal peptide, a 30aa B chain, a 42aa C peptide, and a 45aa A chain. IAG2 precursor has a 27aa signal peptide, a 32aa B chain, a 41aa C peptide, and a 38aa A chain. Both IAGs have 8 cysteine residues located at B chain and A chain, and exhibits similar characteristics as previously described IAG isoforms (4). Different from other crustaceans, they have 9 and 4aa residues between Cys4 and Cys5 in A chain of IAG1 and IAG2, respectively (Figure 4).

### Kinin

Two nucleotide sequences were identified from the transcriptome data putatively coding for two incomplete kinin precursors with 142 and 114aa (Supplementary File 1). Both have no signal peptide. Twelve peptides were predicted from kinin1 precursor, and the first peptide lacks N-terminus. Fourteen peptides were predicted from kinin2 precursor, and the last peptide has no glycine, which is represent C-terminus amidation. Apart from these incomplete peptides, sequences of 11 mature peptides have conserved motif: XFX-A/P-WAamide (Figure 1), e.g., QSFSAWAamide, and QAFSPWAamide, the signature of kinin family (e.g., 45) (Supplementary File 1 and Figure 1).

### Myosuppressin

The predicted 102aa myosuppressin precursor has a 31aa signal peptide (Supplementary File 1). Three distinct peptides were predicted from this precursor, one of which is identical in structure to conserved decapod myosuppressin family, i.e., pQDLDHVFLRFamide [e.g., (45)].

### Natalisin

The predicted incomplete natalisin precursor has 102aa (Supplementary File 1). Six predicted mature peptides were released from this precursor with conserved C-terminal WXXRamide (Figure 1), e.g., EDSNLYSLVSDKEPSEINPFWVARamide, EGESNPYWIARamide, the signature of natalisin family [also called WXXRamide, e.g., (43)].

### Neuroparsin

Three transcripts were predicted to encode neuroparsin precursors with 99-101aa. These precursors are composed of signal peptide and the mature peptide with 12 aligned cysteines (Supplementary File 1 and Figure 1). These mature peptides show highly conserved sequences as previously described neuroparsin isoforms, e.g., *L. vittata* neuroparsin1 is 63% identical/73% positive in protein sequence to *L. vannamei* neuroparsin [e.g., (43, 54)].

### Neuropeptide F (NPF)

Two NPF transcripts were identified to encode 112 (NPF1) and 127aa (NPF2) precursors with 31aa and 29aa signal peptide (Supplementary File 1). Four distinct peptides were predicted from NPF precursors, two of which possess the C-terminal motif RPRFamide, the hallmarks of NPF family members, i.e., ARTDNTAEVLQAMHEASLAGMLSSAEVPYPSRPNVFKSPVELRQYLDALNAYYAIAGRPRFamide, KPDPTQLAAMADALKYLQELDKYYSQVSRPSPRSAPGPASQIQALEKTLKFLQLQELGKLYSLRSRPRFamide (45).

### Orcokinin

The predicted orcokinin precursor is composed of 140aa, and starts with a 22aa signal peptide (Supplementary File 1). Eight mature peptides were predicted from orcokinin precursor, where five adjacent mature peptides separated by “KR” cleavage sites possess N-terminal motif NFDEIDRX (Figure 1), the signature of orcokinin family members; and one (named orcokinin-1), i.e., FDSFTTGFGHS, an identified decapod orcomyotropin isoform (45).

### Pigment-Dispersing Hormone (PDH)

Two transcripts were identified to encode PDH precursors with 83 and 79aa (Supplementary File 1). The precursor PDH1 has a 22aa signal peptide and mature octadecapeptide: NSELINSLLGLPKVMNDAamide, similarly, PDH2 has a 22aa signal peptide and the mature octadecapeptide: NSGMINSLLGIPKVMTDAamide. The two mature octadecapeptides exhibit highly conserved sequences as previously described decapod PDH isoforms (e.g., 43), e.g., *L. vittata* PDH1 is identical to the PDH predicted from *L. vannamei* PDH1 precursor (55).

### Proctolin

The predicted 108aa proctolin precursor has a 22aa signal peptide (Supplementary File 1). Three distinct peptides were predicted from proctolin precursor, one of which, i.e., RYLPT, is identical to the authentic proctolin [i.e., (25, 45)].

### Pyrokinin

One transcript was identified to encode 272aa pyrokinin precursor with a 18aa signal peptide (Supplementary File 1). Sixteen peptides were predicted from pyrokinin precursor, thirteen of which are 7-9aa in length with conserved motif PRLamide, e.g., SPFSPRLamide, GDFAFSPRLamide, the pyrokinin family signature [i.e., (45)].

### Red Pigment-Concentrating Hormone (RPCH)

One transcript was identified to encode incomplete RPCH precursor with 91aa, starting with a 21aa signal peptide (Supplementary File 1). Three distinct peptides were predicted from RPCH precursor, one of which, i.e., pQLNFSPGWamide, is identical to the authentic RPCH [i.e., (45)].

### RYamide

One transcript was identified to encode RYamide precursor representing a C-terminus region (Supplementary File 1). Two distinct peptides were predicted from this precursor, one of which is an incomplete peptide with conserved “RYamide” motif, i.e., SSPSQSELPEIKIRSSRFIGGSRYamide, the RYamide family signature [i.e., (45)].

### Short Neuropeptide F (sNPF)

The 173aa sNPF precursor was identified from the transcriptome data, and starts with a signal peptide (Supplementary File 1). Nine distinct peptides were predicted from this precursor, four of which are 8-11aa long with PXXRLRF/Yamide conserved motif, i.e., GPPSMRLRFamide, SEPSLRLRYamide, KDRTPALRLRFamide, APALRLRFamide, the sNPF family signature [i.e., (45)].

### SIFamide

One transcript was identified to encode 76aa SIFamide precursor, starting with a 27aa signal peptide (Supplementary File 1). Two distinct peptides were predicted from SIFamide precursor, one of which, GYRKPPFNGSIFamide, identical to Gly^1^-SIFamide isoform [i.e., (45)].

### Sulfakinin

One transcript was identified to encode 122aa sulfakinin precursor with a 21aa signal peptide (Supplementary File 1). Five distinct peptides were predicted from this precursor, where two adjacent mature peptides, pQFDEY_(SO3H)_GHMRFamide and AGGDYDDY_(SO3H)_GHLRFamide separated by carboxy-peptidase cleavage sites, possess conserved motif Y_(SO3H)_GHM/LRFamide, the signature of sulfakinin family [i.e., (45)].

### Tachykinin

The putative tachykinin precursor is comprised of 217aa, starting with a 26aa signal peptide. Twelve predicted peptides were released by two dibasic cleavage sites (RK, KK, RR) (Supplementary File 1). Seven peptides have the same sequence: APSGFLGMRamide (Figure 1), a broadly conserved decapod tachykinin isoform [i.e., (45)].

### Terminal Ampullae Peptide (TAP)

One transcript encoding TAP precursor was found in transcriptome data. This precursor composed of a 18aa signal peptide and 70aa mature peptide with 8 cysteine residues (Supplementary File 1). This peptide has 77% identical/92% positive amino acid sequence compared to the TAP predicted from *M. rosenbergii* TAP precursor (56).

### Trissin

The predicted 200aa trissin precursor has no signal peptide (Supplementary File 1). Two distinct peptides were predicted from trissin precursor, one of which is a partial C-terminus peptide, i.e., +EVSCGSCGLECQKACGTRNFRACCFNFQ. It has 89% identical/89% positive amino acid sequence compared to the trissin predicted from C. quadricarinatus trissin precursor (53).

### Vasopressin

One transcript was found from transcriptome data to encode 148aa full-length vasopressin-neurophysin precursor starting with a 19aa signal peptide (Supplementary File 1). Three distinct peptides were predicted from vasotocin-neurophysin precursor, one of which, CFITNCPPGamide (with a disulfide bridge between two cysteine residues), is structurally identical to vasopressin family (i.e., 42,43). One has a 100aa peptide with fourteen cysteine residues, and exhibits highly conserved sequence to previously described decapod neurophysin isoforms (23).

### Peptide GPCRs

**Figure 5:**
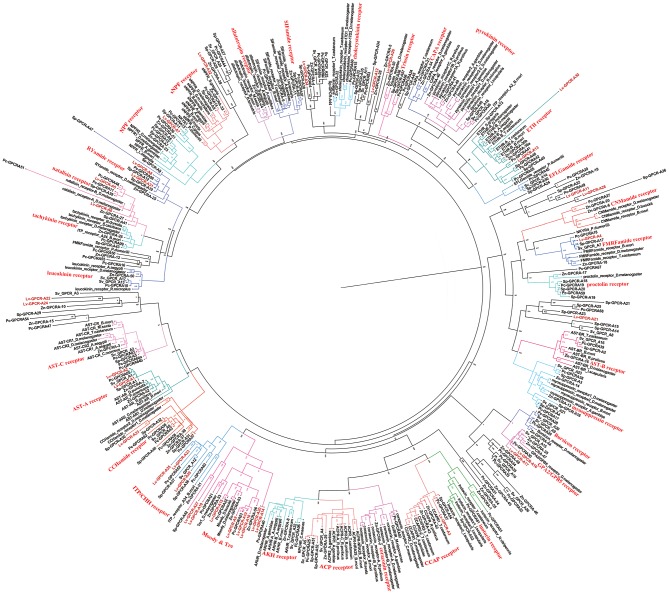
Phylogeny of the A-family peptide GPCRs. Cladogram of peptide GPCRs showing connections in the clustermap of A-family peptide receptors. Lv-GPCR, *Lysmata vittata* GPCR; Pc-GPCR, *Procambarus clarkii* GPCR; Sv-GPCR, *Sagmariasus verreauxi* GPCR; Sp-GPCR, *Scylla paramamosain* GPCR; Zn-GPCR, *Zootermopsis nevadensis* GPCR.

**Figure 6:**
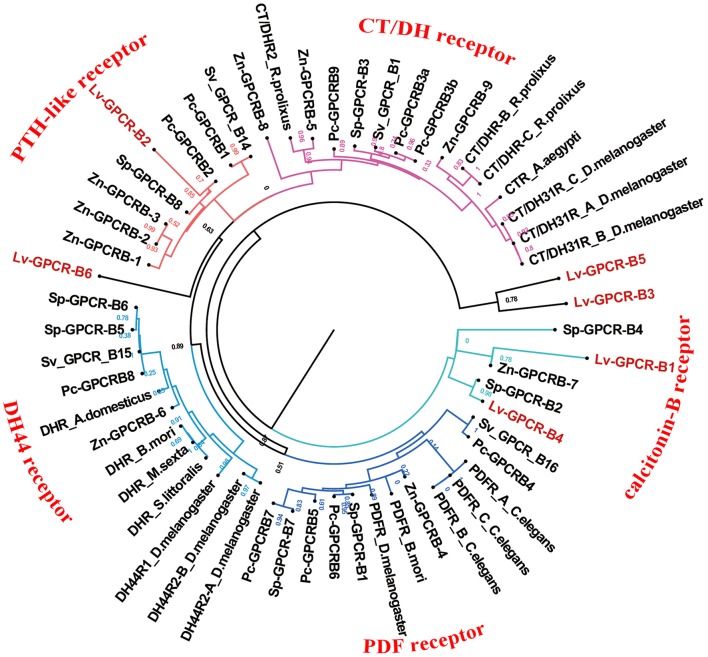
Phylogeny of the B-family peptide GPCRs. Cladogram of peptide GPCRs depicting connections in the clustermap of B-family peptide receptors. Lv-GPCR, *Lysmata vittata* GPCR; Pc-GPCR, *Procambarus clarkii* GPCR; Sv-GPCR, *Sagmariasus verreauxi* GPCR; Sp-GPCR, *Scylla paramamosain* GPCR; Zn-GPCR, *Zootermopsis nevadensis* GPCR.

A total of 40 candidate peptide GPCR transcripts were predicted from *L. vittata*. To identify these GPCRs orthologs, hundreds of known peptide GPCRs from arthropod were collected for building phylogenetic tree. Phylogenetic analysis showed that 28 of these were clustered with known peptide receptor orthologs (Figures 5, 6). Lv-GPCR-A1 and Lv-GPCR-A2 were clustered with the AST-A receptor ortholog. Lv-GPCR-A3 was clustered with CCAP receptor ortholog. Lv-GPCR-A4 was clustered with the FMRFamide receptor ortholog. Lv-GPCR-A5 and Lv-GPCR-A6 were clustered with the natalisin receptor ortholog. Lv-GPCR-A7 was clustered with the NPF receptor ortholog. Lv-GPCR-A8 and Lv-GPCR-A31 were clustered with the RYamide receptor ortholog. Lv-GPCR-A9, Lv-GPCR-A10, Lv-GPCR-A14, Lv-GPCR-A15, Lv-GPCR-A18, Lv-GPCR-A19, Lv-GPCR-A20, Lv-GPCR-A27 were clustered with the Moody &Tre ortholog. Lv-GPCR-A13 and Lv-GPCR-A30 were clustered with the ETH receptor ortholog. Lv-GPCR-A16 and Lv-GPCR-A17 were clustered with the GPA2/GPB5 receptor ortholog. Lv-GPCR-A23, Lv-GPCR-A25, Lv-GPCR-A29, and several putative CHH receptors were clustered with *Bombyx mori* BNGR-A34, which has been defined as ITP receptor (57). Lv-GPCR-A33 was clustered with the CCHamide receptor ortholog. Lv-GPCR-B1 and Lv-GPCR-B4 were clustered with the calcitonin-B receptor ortholog. Lv-GPCR-B2 was clustered with the parathyroid hormone receptor (PTH)-like receptor ortholog.

## Discussion

The RNA-seq and bioinformatics analysis is highly effective for identifying peptides and their GPCRs (23, 26, 28, 37, 43, 58, 59). In this study, 61 transcripts derived from 44 peptide families and 40 transcripts derive from 13 peptide GPCRs were identified. This is the first study on genus *Lysmata* to characterize its peptide repertoire and their GPCRs.

### Comparison of Peptide Sequences

Considering that the *L. vittata* peptide transcripts were computationally-generated from short reads and the mature peptide structures were predicted based on a bioinformatics workflow, the peptide repertoire from one Caridean shrimp, *M. rosenbergii* reported before was chosen for sequence comparison to provide increased confidence of the *L. vittata* peptide sequences/structures reported in present study (43, 50). Overall, sequence alignments of the predicted peptide amino acid sequences in *L. vittata* and full-length *M. rosenbergii* peptide precursors show that all peptides especially the mature peptides are conserved in the two species (Supplementary File 2). For example, the predicted mature peptides of calcitonin (TCYINAGLSHGCDYKDLVGAMAEKNYWDSLNSPamide), DH31 (GLDLGLGRGFSGSQAAKHLMGLAAANFAGGPamide), EH (ASITSMCIRNCGQCKEMYGDYFHGQACAESCIMTQGVSIPDCNNPATFNRFL), and a large number of relative short mature peptides (e.g., PQHYAFGLamide from AST-A, ADWSSMRGTWamide from AST-B, QIRYHQCYNPISCF from AST-C, DAGHFFAETPKHLPRIamide from ETH) from *L. vittata* are identical in amino acid sequences/structures to the corresponding mature peptides reported in *M. rosenbergii*. This suggests the putative peptide sequences in our study are mostly accurate.

In addition to the reported peptides data from *M. rosenbergii*, the peptide repertoire from another *Lysmata* species, a marine shrimp *Lysmata wurdemanni*, was chosen for sequence comparison. The *L. wurdemanni* peptide sequences from the only public *Lysmata* transcriptome data, i.e., the *L. wurdemanni* embryo and adult (brain and muscle) Sequence Read Archive (SRA) (SRR8715485, SRR8715486), was downloaded for peptide mining based on our bioinformatics workflow. A total of 78 transcripts derived from 45 peptide families were predicted from *L. wurdemanni*, mainly significant amino acid similar to those from *L. vittata* (Supplementary File 2). A large number of peptide families were identified from the two *Lysmata* shrimps, but a number of established peptide groups were not identified in each shrimp. No sequences encoding CNMamide, corazonin, elevenin, HanSolin, HIGSLYRamide, or RFLamide proteins were found in the *L. vittata* assembly. Similarly, no sequences encoding Bursicon, ETH, HanSolin, HIGSLYRamide, Hyrg, IAG, or RFLamide proteins were found in the *L. wurdemanni* assembly. Given the significant peptide sequence resemblance between the two *Lysmata* shrimps, we hypothesize that CNMamide, corazonin, and elevenin are likely to be found in *L. vittata*. On the other hand, Bursicon, ETH and IAG are likely to be found in *L. wurdemanni*. The hypothesis was verified when *L. wurdemanni* IAG was cloned from the testicular part of *L. wurdemanni* (60). In contrast, HanSolin, HIGSLYRamide and RFLamide families are likely to be absent in *L. vittata*, as these peptide families have not been identified from the current Caridean shrimps peptide data (43, 50, 61). Of which, HanSolin and RFLamide are recently-identified peptides from the stick insect, *Carausius morosus* (62), and subsequently were found in most Coleoptera species (63), and HIGSLYRamide have been identified only from brachyuran [e.g., (23, 40, 41)]. Therefore, additional transcriptome, peptidome, and/or a genome data can be created to clarify the presence/absence of these peptide families in *L. vittata*.

### Peptides/GPCRs Expression

Given that the experimental design of each development stage single libraries comprised of pooled RNA samples, we did not investigate expression changes statistically. However, it is possible that the expression values presented provides valuable preliminary data to identify candidates for prospective study on PSH species peptides. Similar to expression patterns of previously reported peptides/GPCRs (23, 64–66), stage-specific expression of peptides/peptide GPCRs existed in *L. vittata*, i.e., TPM values of peptides/peptide GPCR transcripts in MP are higher than in EP. In *L. vittata* MP, higher expression levels of the peptidergic signaling promotes the growth of body size and development of testicular part of the gonad. For instance, NPFs, sNPF and IAGs have higher expression in MP, where NPF and sNPF were proved as the feeding behavior controllers in arthropod (67), and NPF has been shown to increase food intake in penaeid shrimp (68). Similar functions of NPF and sNPF are proposed in *L. vittata*. IAG is regarded as the regulator of male sex differentiation in crustaceans (2–4), and high IAG expression in MP suggest that IAG might be involved in promoting masculinization and developing testicular part of the gonad in *L. vittata*. In contrast, a number of peptides show different expression patterns, e.g., TPM values of two PDH transcripts in MP are lower than those in EP. PDH regulates pigment distribution controlling circadian rhythm (69), and also shows different expression levels during the mud crab *Scylla paramamosain* vitellogenesis (6), but the reproductive function of PDH is yet to be proved in crustaceans. TAP shows high expression in both MP and EP, i.e., TPM value of MP and EP is 24.3 and 29.9, respectively. In *M. rosenbergii*, TAP was distributed on terminal ampullae and sperms, as it participates in sperm proteolytic activity and plays a key role in sperm maturation (56). We hypothesized that TAP play similar functional roles in *L. vittata* sperm, in gonad of both MP and EP (15). Moreover, TPM values of neuroparsins and RPCH are higher than 15 in MP. They play a role in regulation in crustacean ovarian development (7, 8). These results imply that transcriptomes of different *L. vittata* gonadal stages should be analyzed to assess reproductive differences in peptidergic signaling in PSH species.

### Key Peptides

Multiple members of the CHH superfamily contain 6 aligned cysteine residues, and nine transcripts of these peptides were found in this study. Given that CHH1-4 some regions in this gene have the same sequences, we speculated they are from different alternative splicing of one gDNA, and this phenomenon is common in type-I CHHs (1, 70). The C-terminal motif “AIAXX” of CHH5 and CHH6, members of type-I CHHs, seems to be more common in Caridean shrimps than other crustaceans. In type-II CHHs, MIH, and GIH show high similarity in sequences, with MIH participating in molting and GIH regulating reproduction process. These genes have been subdivided based on primary structures and motifs (71). However, this rule does not apply for type-II CHHs in Caridea, making it difficult to subdivide MIH and GIH based on primary structures in this species. Therefore, we named two *L. vittata* type-II CHHs as “MIH/GIH”. These *L. vittata* MIH/GIHs were clustered into two subgroups, in which MIH/GIH2 was grouped with *M. nipponense* GIH. Type-II CHH was reported to inhibit ovary development (72). This suggests that MIH/GIH2 could be GIH. MIH/GIH (MIH/GIH1) was grouped with the predicted *M. nipponense* MIH, implying that may be MIH. CHH-MIH-like peptides were clustered with some predicted shrimp ITPs, but it is not appropriate to name these peptides as “ITP,” because they neither have the typical CHH-PRP of ITP, nor are they similar to *L. vannamei* ITP, which modulate osmoregulation in shrimp (73).

Two unique mature peptides of IAGs were found in a species for the first time in this study. In the Chinese shrimp *Fenneropenaeus chinensis*, two IAG isoforms were identified from one gene (74). Three *C. sapidus* IAG genes were identified from AG, hepatopancreas and ovary. However, mature peptides of these genes are identical (75–77). Only one IAG was cloned from *L. wurdemanni*, and this IAG showed higher sequence similarity with those of the genus *Macrobrachium* than with the two *L. vittata* IAGs (60). In this study, expression levels of two IAGs in MP shrimp were higher than in EP shrimp, of which, the TPM value of IAG1 was 76 in MP shrimp, but not expressed in EP shrimp. In *L. vittata*, it was reported that the male external sexual character had disappeared whereas testicular part had some degree of degeneration in EP shrimp (15), of which were ascribed to the down-regulation of IAGs. In *M. rosenbergii*, silencing of IAG not only arrested the degeneration of male secondary sexual characteristics and testis, but also prevented testicular spermatogenesis (2). *M. rosenbergii* IAG dsRNA injections canceled spermatozoa in the sperm duct and testis (2). In *L. vittata* EP, both ovarian and testicular parts were mature, testicular part being filled with many spermatozoa (15). It seems that spermatogenesis was unaffected following down-regulation of IAGs in *L. vittata*.

In many species, including in *L. vittata*, CFSH has one to three paralog genes (43). In this study, two CFSH transcripts were almost not expressed in EP, which is the ovarian mature stage. In female crabs with CFSH knock-down, the brooding and mating systems were abnormal at puberty (5). Analysis of expression pattern of *L. vittata* CFSH revealed that it might be involved in the development of female phenotypes at puberty, rather than the vitellogenesis and ovarian maturation.

Two sexual systems, two sex differentiation hormones, and two unigenes of these hormones exist in an individual, implying that a complicated sexual regulatory network exists in *L. vittata*. RNAi should be performed to reveal the discover bisexual mechanism regulated by sex differentiation hormones in *L. vittata*. This species is expected to be an ideal model for RNAi experiments because it is small, has a transparent body and short reproductive cycle (22).

## Summary

Total RNA was extracted from *L. vittata* mixed tissues and used to mine peptides. More than 60 peptide transcripts were identified. However, this method has the following limitation, i.e., the expression levels of some tissue specific (e.g., eyestalk) peptides may be too low to assemble long enough transcript encoding complete precursor. Here, 15 peptide transcripts encoded incomplete precursors. Some of them showed low expression levels, such as CFSH1a, CHH3, GPA2, and RYamide. Furthermore, the peptides contained several similar peptide paracopies making it difficult to assemble the complete CDSs encoding such precursors (43), as in the cases of AST-A, kinin, and natalisin in our study. Notably, 28 predicted Lv-GPCRs were grouped with known peptide GPCRs, including: AST-A receptor, CCAP receptor, FMRFamide receptor, natalisin receptor, NPF receptor, RYamide receptor, Moody & Tre, ETH receptor, GPA2/GPB5 receptor, CHH receptors, CCHamide receptor, calcitonin-B receptor, and PTH-like receptor. Together with the identified peptides in *L. vittata*, we speculate that AST-A, FLRFamide, natalisin, NPF, RYamide, ETH, GPA2/GPB5, CHHs, CCHamide, and calcitonin ligand-receptor pairs are expressed in *L. vittata*. In conclusion, complete peptide/GPCR genes remain to be cloned and confirmed through PCR experiments. Moreover, *in vitro* ligand-receptor binding tests are required to determine Lv-GPCRs. Nevertheless, identification of peptides and the associated GPCRs in *L. vittata* extends our knowledge on peptidergic signaling in PSH species, and provides experimental basis for further studies on the of function peptides in reproduction. This will promote aquaculture development of this shrimp.

## Data Availability Statement

The datasets generated for this study can be found in the SRA accession: PRJNA561673.

## Ethics Statement

The study was approved by Xiamen University animal care committee.

## Author Contributions

CB: conceptualization, data curation, software, writing-original draft. FL: Sample collection, data curation. YY: project administration, editing. QL: sample collection. HY: conceptualization, editing, supervision.

## Conflict of Interest

The authors declare that the research was conducted in the absence of any commercial or financial relationships that could be construed as a potential conflict of interest.

## Supplementary Material

Supplementary File 1 Peptide precursors and their expression levels in male phase (MP) and euhermaphrodite phase (EP) of *L. vittata*.

Supplementary File 2 Comparative sequence alignment of peptide precursor in *L. vittata* with *M. rosenbergii* and *L. wurdemanni*.
