# Supplementary material for: Identification of Peptides and Their GPCRs in the Peppermint Shrimp Lysmata vittata, a Protandric Simultaneous Hermaphrodite Species
Source: Front Endocrinol (Lausanne). 2020 Apr 30;11:226. doi: 10.3389/fendo.2020.00226 (PMC7212414; doi:10.3389/fendo.2020.00226)
Supplement: Supplementary File 1 — Peptide precursors and their expression levels in male phase (MP) and euhermaphrodite phase (EP) of L. vittata. [file Data_Sheet_1.PDF]

Supplementary file1

Identification of peptides and their GPCRs in the peppermint shrimp *Lysmata vittata*, a protandric simultaneous hermaphrodite species

Chenchang Bao[1], Fang Liu[2], Yanan Yang[1], Qi Lin[3], Haihui Ye[2]*

[1]School of Marine Science, Ningbo University, Ningbo, China
[2]College of Ocean and Earth Sciences, Xiamen University, Xiamen, China
[3]Fisheries Research Institute of Fujian, Xiamen, China

∗Correspondence: haihuiye@xmu.edu.cn

Adipokinetic hormone-corazonin-like peptide1 (ACP1)

MFRWQIIMAVVCLSLAPALSQITFSRSWVPQGKRSAGTVVPGSPAVAAAGEVDGGLGDAFCLEARLMALSEVGAHI
AELMDETSKAEASLALRLKHSLMARRSK*

Adipokinetic hormone-corazonin-like peptide2 (ACP2)

MHRLTVLLAVACLVFGPAMAQITFSRSWVPQGKRSAQAGSLLSSGDIADTCQEAKLTVLTQVANYVTRLMEETSD
MGSDEASLAYHLRQAQIARRRRMA*

Agatoxin-like peptide

MGSKVLVMVLAFFLVLSIAMAQPLIEEDHEDEVGQPDADYTADLLERLLARTSQKRDDMGGPIKRWRSCIPRGGSCTHR
PKSCCNSSSCRCNLWGTNCRCQRMGLFQQLGK*

A-type allatostatin1 (AST-A1)

MVVRHGGIRAYSIAAFLLLLGGCVRTQEDYYDSDVEELLEGNDLEFQPQPNYGWEYGKRHNDYAFGLGKRSPGY
AFGLGKRDRLYSFGLGKRENLYAFGLGKKSGTYNFGLGKRSAREVVPGDFDDEQPMEFPSSSPSSSPSSPTPSSSSPS
SSSIRTKREAESNEDVSEEKRKQYAFGLGKRGGEDDQKRSKSFSFGLGKRQDSDEEKRDRSYSFGLGKRDPDMDK
KSQQQYAFGLGKREPGTEGDTQEADLEKRRQNYAFGLGKRGEDFIDKRALQYAFGLGKREPSLEKRPRNYEFGLG
KRGDNPDMDKRPQHYAFGLGKRGEEDTEKRAQ*

A-type allatostatin2 (AST-A2)

+RPQHYAFGLGKRDSDIDLIKKPQHYAFGLGKRDFEDDLEKRANYAFGLGKRYSAEDVNRMYAFGLGKRSAEYDLIGE
DDLVDDDDEFDDDDDSDIDDLDEEDLVEYQEQLKRAASYGFGLGKRAGHYAFGLGKRGGSYAFGLGKRTPGYAFGLG
KRPSNAYSFGLGKRSRSYQFGLGKRAGSYAFGLGKRAGSYAFGLGKRENPYAFGLGKKAGHYSFGLGKRSSPYAFGLG
KRTSPYAFGLGKRTSPYAFGLGKRTSPYAFGLGKRTSPYAFGLGKRDDSSSSSSGLGRRSGSYSFGLGKRVPGSYAFGLGK
RETNDDDDQPQEPEPTGAS*

A-type allatostatin3 (AST-A3)

+YAFGLGKRDSDLEKRPQHYAFGLGKRDFNSYLDKRPQHYAFGLGKRESNLDLGKRPQHYAFGLGKRESDSDLEK
RPQ+

B-type allatostatin (AST-B)

MMQQHALRNASLLALMVIAVTQLTTALDPVPETPHTEVKRADWSSMRGTWGKRSDGDAVALEEPDDKRSGWNKFQG
SWGKRSDEMTDAELQMAEDKRANWNKFQGSWGKRDGDFDGTDDKRGGWQNFQGSWGKRTADLMAADDKRGGW
QNFQGSWGKRDDDLMDAEEKRANWNKFQGSWGKRNNWSSLQGSWGKRDIPAEILEELEKRGGWSSLQGSWGKRAW
QNLHGAWGKRNNPAESTDDVVDDEDEQEQEEEALQRALLSPVALARLMNASPQKRGWTLWGKRPQYPARVAPRSAN
WSSLRGTWGKRSGDWSSLRGAWGKKNADWSQFKGSWGKRALGDEDAASQVA*

C-type allatostatin (AST-C)

+FSSSSSSSPTSDHSPSPSINTHTQRIQKRTVGKEPSPEELAVLKDLILSRVASELSENLREQQPLAKRVKEESERQKEA
EEEAKEAEEKEAMLAEAKAKRMFGGSPLSGLPGELPTMKRQIRYHQCYFNPISCFRRK*

C-type allatostatin (AST-CC)

+PPANMNGRAGGGSTTSWAAGRSNVPQHGRGSRPCCVSNSKSGPSSSSFSTSQSRSIPLLLLLLLGILLPFVMPAAAAAVP
HVAPQRPMYIEAIRPVMTNSAMPPSAPQQVVLQQIPDNVPPPRKRAAIVLDKLMFALQKALDDTPNATPPGPQQDFPRN
RAFAAGPMDLQRRGNNNDGRLYWRCYFNAVSCF*

C-type allatostatin (AST-CCC)

MVARSSVAFVLVALMAVLAMTSVEAKAIPDREPQGYAQGQQLMDLQLPYGNHLVDDDGSLDTALINYLFAKVMVDRL
RNNVDVKDLQRKRSYWKQCAFNAVSCFGKRK*

Bursicon hormone alpha subunit

MSSRRMISIMGVALTLVGVLMSDIAFADECSLTPVIHILSYPGCNSKPIPSFACQGRCTSYVQVSGSKIWQTERSCMC
CQESGEREATVTLNCPKARVGDPKRRKILTRAPVDCMCRPCTDVEEGTVMAQEIANFIADDPMAHVPFLK*

Bursicon hormone beta subunit

MWSWWTWIVVFVIGVCQMTHAKGYRSECETLPSTIHISKEEFDEAGRLIRTCEEDLAVNKCEGSCLSKVQPSVNTP
SGFLKDCRCCRETHLRSREVILTHCYDVDGNRLVGGKGQLSLKMSEPADCQCAKCGDSTR*

Calcitonin

MRHGCWLACISMMAVVATVLSAHVQPIPESELMEIPERLRELLLVRKLISSLNAAEPLPELQARPGQTIRKRTCYINAGLS
HGCDYKDLVGAMAEKNYWDSLNSPGRRRRSLENSYSSSSSPSSSSSSSSSSLYPSSSSSSSSSPSSLGDYQASVSLEASGET
Q*

Calcitonin-like diuretic hormone (DH31)

MNNSALVFISLVAAFFFVSSVNSAALNRETRAVVEIDDPDYVLDLLTRLGHSIIRANELEKFVRSSGSAKRGLDLGL
GRGFSGSQAAKHLMGLAAANFAGGPGRRRRSSDDNGLQNPHLEEDNYTQDHHDAVATNAAAGSTR*

CCHamide

MSSLKTYSFLLLIFPLVIVCSPVTSARRVPKGGCLNYGHSCLGAHGKRASPSAISPSMLGVLLEALSSRPGELAVLSHSRG
DAAVGTNTLHRAVSHHNRYPSARIVSSPTAQEDEEAMARAAMPDTFRSLLASRLNGRDVRSRDGVDEESEADVVYPMN
VEDYADDRIRRSAVEKVPVSKGGQKEISDEWEQEKEGGNNHREENDNRNLRYETWLRR*

CRF-like DH44

+QQQQQQQPPLSLLEDLPSQDFTQDDIQQLLARHPDNAPAASIPSLNSYDGSEGLPYVVYRLQEGLAGAGLPSSGLASDVT
NLDNPDWMTIDPRYYLLSQYLDHPEEATDSDSGSNSMTPVRKIRDSSNPSSVSSSSNSNFDNSNSKAKRTWPHGFSRRRN

SGLSLSIDASMKVLREALYLEMARKKQRQQMLRARHNQALLTTIGKRDVQRQLQQQELEDDQQEHLRAERI*

Crustacean cardioactive peptide (CCAP)

MSHHQSISGRTVSLLAAALLLLVVVLQATASPVAKRDIGGLLDGKDKRPFCNAFTGCGKKRSDASVEALASGAEL
DDLAKHVLTEAKLWEQLQNKMEVMRSLAARMDDHPLYRRKRSPAPESRQQQITASSQQQQQKTENQ*

Crustacean female sex hormone 1a (CFSH1a)

MAPHRPPSSTSRFCSSLTLVGAVVLALAQQQGVKSDLDLPAPYPKPFFLREWEEDDLRDFKWSDALRNSLKRNKDGDD
ELGNDPLQYFSEEQVNAAMKAEYKVVPHPVVYTSQILREGVNCSSIRMNLHRNHVKPELQLRPDWIHKSDFIGDCPAH
YVTKELPPMYSPAVILEAVCTCGGSQCSRSGHQCVPVSRHVPVWVRRGPNFH+

Crustacean female sex hormone 1b (CFSH1b)

MEQHQSHSKSPLRLCLMASFCLAAFLLQEPSEATLNLPSYQLGSGGWDENIPKHIKWRAEAIRILKQDELSTDKLQ
YFSEDQVNEASKLEYKVVPEPIMYTSQIIHKGVNCSSIRTGLHKNHIRPELQLHPEWIHTSELIGSCPTHYVTKELPP
MYSPSVVVEAVCTCSGSKCSREGHQCLPVSRYMPVWVRQGPNFHVLDVEEITVACACVRRPSASGNFIYASAVHS*

Crustacean hyperglycemic hormone1 (CHH1)

MVPNSLMWSAVFLVGLAFSWTTSASARSADGLARIERLLASSASESSEPSTALAVQEEDQAVSKRAILDQSCKGIYD
RELFRKLEKVCEDCYNLYRKAHVGVECRSNCYGNLIFRQCLDDLLMMDVVDEYIKKVQMVGK*

Crustacean hyperglycemic hormone2 (CHH2)

MICNGTMWSTLMVLVLVLSSNSNPAMARSAEGLARIEKLLSSSSSASPSVPSQPSSPLTAALTQRGHSLPKRAVLDQ
SCKGIYDRELFKKLDRVCEDCYNLYRKPYVGIDCRRNCFLTKTFDQCVVDLLLDEKEFGEIRDHVAFI*

Crustacean hyperglycemic hormone3 (CHH3)

+MARSAEGLARIEKLLSSSSSSASPSVPSQPSSPLTMDFTQGDHSLSKRAVLDHSCKGIFDRELFKKLD+

Crustacean hyperglycemic hormone4 (CHH4)

MWSTLMVVILVLGSNNNSAMARSAEGLARIEKLLSSSPSSVSPLVPSPLTEHSLRKRTVLDQSCKGIFDRELFKKLD
+

Crustacean hyperglycemic hormone5 (CHH5)

MISDNMLRTTSSVLVLVLVLSSQSLLVQSAEDLRPIQKLSASSSSSPSDGLGSLVKGHPIAKRSYGMSCKGPGYSPEL
FQQLSKVCDGCYNMYRQYEIDIECRRNCFGTGVFLQCVAYQLLDVEEHMGIREAIAEF*

Crustacean hyperglycemic hormone6 (CHH6)

MFSYSTMWMSVVLVGFVLSSNSISLVQSATTVRDIDNLSDSSSSSSSLTSPVKGHGITKRSLGKSCKGMHFDIELFQKLD
KVCEVCYNLYRRADVAIECRRNCFGTSVFRQCVAAQLLDVEEHMDIQQAIADF*

Molt-inhibiting hormone/gonad-inhibiting hormone1 (MIH/GIH1)

MVFSTTPNFSVQRAVATAILIVSLLLVSGTSARYLDDECPGVMGNRDLYEKVARVCDDCSNIFRKNDVGARCRKDC
FYNEDFLWCVYATERYDDVEQLNRWMSILKAGRK*

Molt-inhibiting hormone/gonad-inhibiting hormone2 (MIH/GIH2)

MPAQLRQRVSLRSLTSVLVTLAVFGLLVADETSARFLDDECKGVMGNRDLYEKVVRICDDCDNIFRLNNIGHKCRK
NCFYNMDFLWCVYATERREELDQLNRSMSILRAGRK*

CHH-MIH-like peptide

MSQRSCCQSSYSQFILVLGLLLLTQNAAANYIRLRPNTYKEFQFLKCQGEFDKEQYSALNRLCDDCHNLFRDPEVL
LECKADCFQNSLFPACVSALLLDHKKPELNKMIYTVSGRKPLAYSLVNDSSAMASLT*

Ecdysis triggering hormone (ETH)

MSWMVAVAVLATMVSLAHSDAGHFFAETPKHLPRIGRRGDLPPLVNLLNEEEPRGSSGGGAAGSSMTEALAKVDGDSD
GCVSLSELLRIPIIRIAILLQNPALITPHVDAEANESSDVYASDRRPEPRLLRYLKK*

Eclosion hormone (EH)

MSFKAQVRFVAVSVVCLIVLASLTEAASITSMCIRNCGQCKEMYGDYFHGQACAESCIMTQGVSIPDCNNPATFNRFLKR
FI*

EFLamide

MIAGWQCVLPSLILCCTLCSTHPTLRQDESKAVVEKRSSGYPYDPVLHLFLVAMSNPKTNQQSPQLLNRGVRRIGSEFLG
KRSVENVEDHKITPRCDDCDSEDADDLKKEQLSYTGQYDYDDERKTTEASENSDQNTYDAPVKRNVRGFYGGPNRDG
LKNFFSLLMSKKMGSEFLGKRMGSEFLGKRAMGSEFLGKRAMGSEFLGKRAMG+

FLRFamide

MMIIAAWISLGALSCCTYALAPPVVSGLEPPTSDSGNSENLSVPDKRILKYLLPSSQSWGESGANAPVPTGQEGSKRGYV
DRNFLRFGRSEGEKRDVGKNFLRFGRGGGEDYEDDEYLSPALTDVDKRDRNFLRFGRPDLEEFGMETSPLAFGQGLKE
EDLVENEKRAAHKNFIRFGRGNKNFLRFGRNYNKNFLRFGRSVDSQTFCEDCEDSNINKHSTTSSTSSSSSSSNLSAGHGI
ESNNKHLNEAIPSEVSDSSAASEERDTRRSKRSAPMYYDYAVVPSHSPASWAREFQPAEEEEVDDTELVDLQDVSKRNA
YNRNFLRFGRDRNFLRFGKREPSDSSSVMVMAPAQYPRIIRASNRNFLRFG*

Glycoprotein-A2 (GPA2)

MVKVWIVLVAYLVASSAAFQHAWQTPGCHKVGHTRKISIPECVEFDITTNACRGYCE+

Glycoprotein-B5 (GPB5)

+VTLGAAWLLVLVLLMPVAAIDPISTLECHRRQYTYKVHKSDDEGRMCWDYINVMSCWGRCDSNEIADWKFPYKRSH
HPVCMHETTQLTEVTLRHCDEDASPGTELYTFHEATRCSCSVCKSSEASCEGIRYRGARRAPRAEIPRG*

Hyrg1

MNLLGFIVVIMAAIFGVSQAYPEPAVIVDGRPNMIPDGYIQAPRFHYRGFQKPIPKYDWS*

Hyrg2

MNVLGFIVVILAAIFGISQALPEAAVIVEGRPNRAPDDGYVQAAPPRFHYRGFQKFVPKYDWS*

Insulin-like androgenic gland hormone1 (IAG1)

MEPGHLSALLLLLSCSVLLILLPHSTCGYEIKCLAHDFDCGNLSGTLSLTCKTYYDYYRERRSVDLDLSSLENSHSH
NAGGIPIKIFGEMSREDANQVLKSRGGRYRRSYVTAYDECCKRVDDGGPHCTYNEVVGYCEELHAGVNTCNRSPG
H*

Insulin-like androgenic gland hormone2 (IAG2)

MGPAYLSMVLLLSSSTYLTLLPQETSGYNITCLAFDFDCGQLADTMSLLCKVYRSHIDERRRRSVNQSQPSPLRNFQGIRT LPFKMYEELTQETTNTSLNIRGGRFRRNTRRNTTNVYNECCQQPNCTYNDLREYCEILQDGIYTC*

Kinin1

+NAWAGKRADDLDKRQSFSAWAGKRESDPEKRQAFNAWAGKRGTEATKRQSFSAWAGKRNDDFEKRQAFSPWAGKR DLENQHAFSTSSATQTRLNEKRQAFNAWAGKRYDENFNNADNHDETLLTLHNLNKQIYNVPLRKDSL+

Kinin2 (maybe artefact)

+NYDKRQAFSAWAGKRNNDFDKRQGFSAWAGKRDDDLDKRQSFSAWAGKRDDDLTKRQSFSAWAGKRDDDLDKRQ AFNAWAGKRDGDLDKRQAFSPWAGKRNDDLDKRQAFNAWA+

Myosuppressin

MVFGSSFSAWCSLFVVSVAVAMAVFAGVGEAMPPPICTDPKLPLSPYAQKLCTALTNIAEFSRAMEEYLDAKVIKNSMPV NEPEVKRQDLDHVFLRFGRSQQ*

Natalisin (WXXXRamide)

+NPYWISRGKKEDSNLYSLVSDKEPSEINPFWVARGKREGESNPYWIARGKKEPETAPYWVSRGKRTSNDASSSFWVSR GRKSDPKNSGKDDPSFWIARGRRN+

Neuroparsin1

MKSCIVYIMLSVFCLLLLLQNSEGAPRCTQHDRPPAEKCVYGTVLDWCRNLVCAKGPGESCGGHQWEMGKCGEGTFC SCGECTGCSVITEVCYPSALVC*

Neuroparsin2

MEKMLSTRTLLVVNILILVLIALISESCGAPNCSTTRHHEVNCTYGTYVDWCRNTVCAKGPGQTCGGQWWERGKCGEG TYCTCGFCSGCSVDIECWFGTFC*

Neuroparsin3

MNQSRSVLFMVFVAVFLLLLTQFIKASPHCPSTRRIEEDLSKCQYGTAIGWCGNLECAKGPGERCGGNWLEHGSCGEGM YCGCGYCAGCFIVECAPRKFC*

Neuropeptide F1 (NPF1)

MRHTAALATSKSMVIVAVAVVASLLMSSASSARTDNTAEVLQAMHEASLAGMLSSAEVPYPSRPNVFKSPVELRQYLDA LNAYYAIAGRPRFGKRGGSTPLRSTSQDDLFDY*

Neuropeptide F2 (NPF2)

MFTRAGQVWAALLVGVVVVSVMQMGGVEGKPDPTQLAAMADALKYLQELDKYYSQVSRPSPRSAPGPASQIQALEK TLKFLQLQELGKLYSLRSRPRFGKRSEYAVPPGDALMEASERLLETLARR*

Orcokinin

MTVQAFSALLLTCALFFVVASANPVKTATNGATAAQHDAGYTDNAPVKRFDSFTTGFGHSKRNFDEIDRSGFGFNKRNF DEIDRSGFGFNKRNFDEIDRSGFGFNKRNFDEIDRSGFGFNKRNFDEIDRSGFGFVRRSAE*

Pigment-dispersing hormone1 (PDH1)

MQSGLVAALVLMVAVSTMMTSAQEELKYPERQVVAELAAQILRLARGGPWGTVAAAAGPHKRNSELINSLLGLPKVM NDAGRR*

Pigment-dispersing hormone2 (PDH2)

MQSKIVVVVVALMVAVAFSLAAAQQYDLLTTEKQLVSELAAQILRVTHLPWTDASAQKRNSGMINSLLGIPKVMTDAGR K*

Proctolin

MARTVLVVLMAALVLVATVSEARYLPTRGDDSRLEEIRELLREILERTADGNSSSGSMNSRTAAAGFGYDKRYIYKRSVA GDNMAQAAVAAPALNGGERMPQLFNLAQ*

Pyrokinin

MLLLLITSSSSSSSSSSSQPEVNSNNDVDVGSSWWWSKAAIRRSPFSPRLGKRDEAHYGLVYDDDDDEEEDDFDTFDDD DDLNDIFEDSAAQSYSDETEVVSPQRLAVRSALVPRLGKKAVAFSPRLGKRVTAFIPRLGKRGDFAFSPRLGKRGDFAFSP RLGKKADFAFSPRLGKRGDFAFSPRLGKKADFAFSPRLGKRGDFAFSPRLGKKSDFAFSPRLGKRGTAFIPRLGKRGTGA STSPSEDSWRTNANDVSQQLQQRSVAFSPRLG*

Red pigment-concentrating hormone (RPCH)

MVRSGISLVLAVLVLVSCVSAQLNFSPGWGKRATVTGGAGSEGGAQIHSASGLALPAAASTVARGTAGDNCATIPISTVM HIYRLIKREAS+

RYamide

+SSPSQSELPEIKIRSSRFIGGSRYGKRSGTAMVPDVSSLVPSVSEDDSEAPATLLVGDSVVCLLIDLPDIYKCLKKTANEES TN*

Short neuropeptide F (sNPF)

MGVGVLKWCAAALFCCLILAQVASSMPAEPDYDALNDVYDWLSSHGVERRGPPSMRLRFGKRGSGIRSWQQVSQRSE PSLRLRYGKRTVTDEAEPLLDHELVRKDRTPALRLRFGKRTSDYGQDEPFGDTDFVRQDRAPALRLRFGKRDVATYGQE ETGTGVAAATATASQEQ*

SIFamide

MSVQTRLVVAVVVVLVVLAVFTDHASAGYRKPPFNGSIFGKRSGGDALYEPGKALASACQVAVEACAAWFPGSEKK*

Sulfakinin

MQSVMRTTSLTCAALVALVAAAVLVSGEVAAASKPSLALARRLAPAIRHKLEGGHFSPALMEEIVADFEDPELMDFYDAE KRQFDEYGHMRFGKRAGGDYDDYGHLRFGRSLGQNRQLPKRH*

Tachykinin

MMRAGICLVLMGVLMMLGVVASVVDAQEPSEREERRAPSGFLGMRGKKNDYLLQEDDYLMNDPIAARIEAAKSLPIRG KKAPSGFLGMRGKKSDEEVYGNDLADYELEALLKRAPSGFLGMRGKKAPSGFLGMRGKKAPSGFLGMRGKKMYDD DVEMDALIEALSAMAEGQHQKRAPSGFLGMRGKKTIYDDQTDESDDLTAGGVDKRAPSGFLGMRG*

Terminal ampullae peptide (TAP)

MKWFALFLLMAMVCVSSQEDIYSKSEAEQIEILGPLLKKLLMPRPRPVYQIHQLLRVHVEKCCNAQPVMDCCTPA
MCCQLSLDSCCKK*

Trissin

+EVSCGSCGLECQKACGTRNFRACCFNFQRRRRSQAPSSSHSLKGLEDPEAVDIHLVLKSPVTAVSRSHESQLPNIIRFISR
LADDSFRSPVQLKDLQLYHPAIPSFLQSSSEDEEEEEVEEEVELEERDNGLTPRSQGVAEGRSLPFGEAGAGSGVEADTLN
RLVEMAYRHPSSPSSSSSLLHRQIQQAHYPPSPSKIRK*

Vasopressin

MQLSVVLVVISLIMGYGNACFITNCPPGGKRSMPSSHIGHTRTCTSCGPGLQGRCMGPEICCGEGIGCFLGTREAQICRTE
NLIPVTCNNSDLKTCGAARSGRCASEGLCCTEAKCEFDIRCIQDGGSQIGRRIPFSSSTDTEDQWNL*
