# Supplementary material for: Identification of Peptides and Their GPCRs in the Peppermint Shrimp Lysmata vittata, a Protandric Simultaneous Hermaphrodite Species
Source: Front Endocrinol (Lausanne). 2020 Apr 30;11:226. doi: 10.3389/fendo.2020.00226 (PMC7212414; doi:10.3389/fendo.2020.00226)

ACP

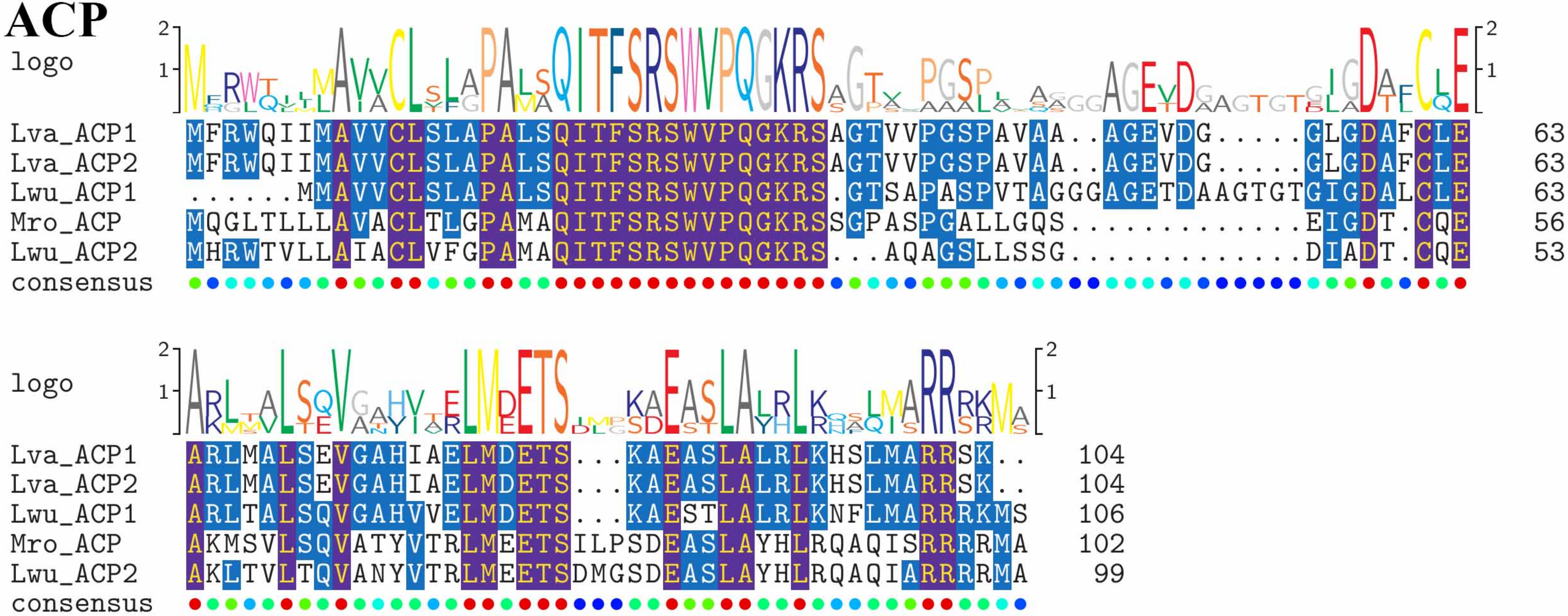
logo
Lva_ACP1 MFRWQIIMAVVCLSLAPALSQITFSRSWVPQGKRSAGTVVPGSPAVAA..AGEVDG.....GLGDAFCLE 63
Lva_ACP2 MFRWQIIMAVVCLSLAPALSQITFSRSWVPQGKRSAGTVVPGSPAVAA..AGEVDG.....GLGDAFCLE 63
Lwu_ACP1 .......MMAVVCLSLAPALSQITFSRSWVPQGKRS.GTSAPASPVTAGGGAGETDAAGTGTGIGDALCLE 63
Mro_ACP MQGLTLLLAVACLTLGPAMAQITFSRSWVPQGKRSSGPASPGALLGQS.............EIGDT.CQE 56
Lwu_ACP2 MHRWTVLLAIACLVFGPAMAQITFSRSWVPQGKRS...AQAGSLLSSG.............DIADT.CQE 53
consensus
logo
Lva_ACP1 ARLMALSEVGAHIAELMDETS...KAEASLALRLKHSLMARRSK.. 104
Lva_ACP2 ARLMALSEVGAHIAELMDETS...KAEASLALRLKHSLMARRSK.. 104
Lwu_ACP1 ARLTALSQVGAHVVELMDETS...KAESTLALRLKNFLMARRRKMS 106
Mro_ACP AKMSVLSQVATYVTRLMEETSILPSDEASLAYHLRQAQISRRRRMA 102
Lwu_ACP2 AKLTVLTQVANYVTRLMEETSDMGSDEASLAYHLRQAQIARRRRMA 99
consensus

Agatoxin-like peptide

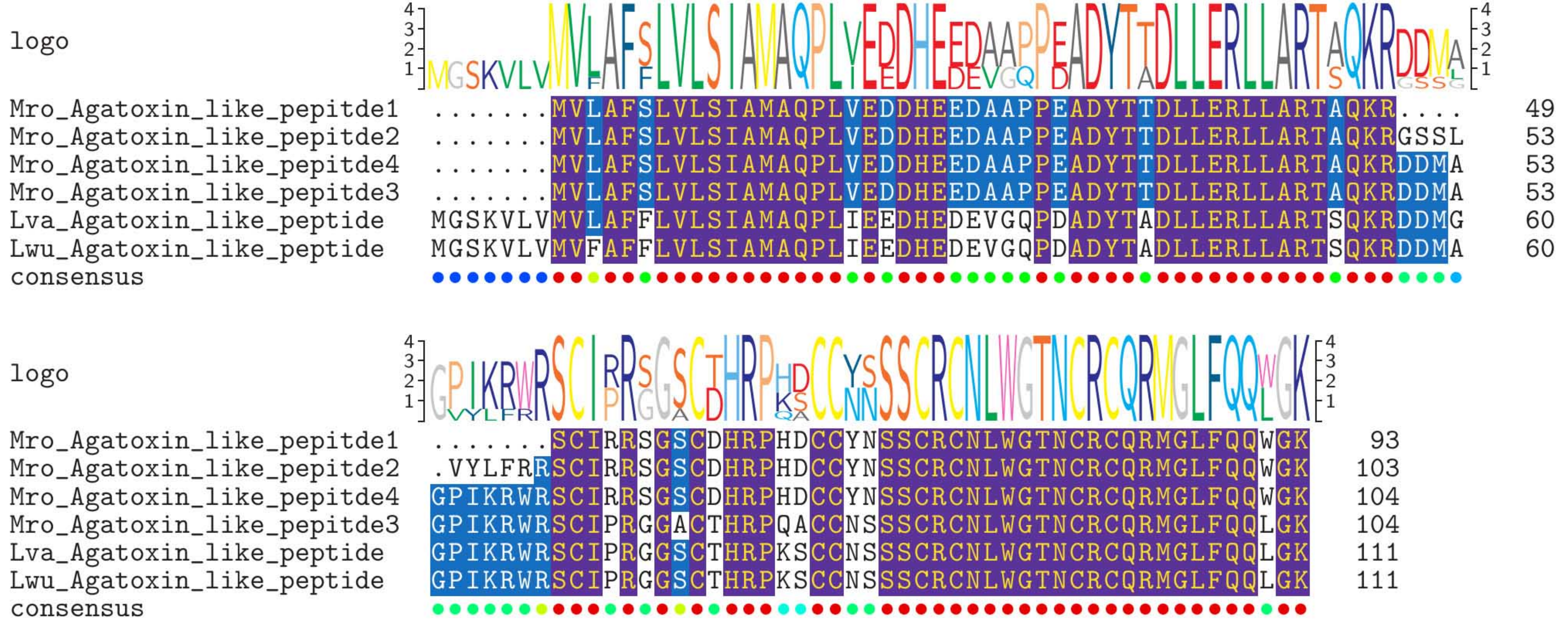

AST-A

```
logo
Lva_AST_A1  .........................................MVVRHGGIRAYS.IAAFLLLLGGCVRTQE   28
Lwu_AST_A1  .........................................MLVRHGGIRAYS.IAAFLLLLAACVKTQE   28
Lwu_AST-A3  ......................................................................    0
Mro_AST_A   .........................................MVVRYGGFRAYSVIAAFVLLLGGCVRSQE   29
Lva_AST_A3  ......................................................................    0
Lva_AST_A2  RPQHYAFGLGKRDSDIDLIKKPQHYAFGLGKRDFEDDLEKRANYAFGLGKRYSAEDVNRMYAFGLGKRSA   70
Lwu_AST-A2  ......................................................................    0
Lwu_AST-A4  .............SDIDLEKKPQHYAFGLGKRDFEDDIEKRAHYAFGLGKRYSADDANHMYAFGLGKRSG   57
consensus

logo
Lva_AST_A1  DYYDSDVEELLEGNDLEFQPQPNYGWEYGKRHNDYAFGLGKRSPGYAFGLGKRDRLYSFGLGKRENLYAF   98
Lwu_AST_A1  DYYDDDVEELFEGNDLQFQPQPNYGWEYGKRHNDYVFGLGKRSPGYAFGLGKRDRLYSFGLGKRENLYAF   98
Lwu_AST-A3  ......................................................................    0
Mro_AST_A   DYYDNDIEEIYEGNDAQYEPQSNYGWDYGKRHNDYVFGLGKRSPGYSFGLGKRDRTYSFGLGKREGLYAF   99
Lva_AST_A3  ......................................................................    0
Lva_AST_A2  EYDLIGEDDLVDDDDEFDDDDDSDIDDLDEEDLVEYQEQLKRAASYGFGLGKRAGHYAFGLGKRGGSYAF  140
Lwu_AST-A2  ......................................................................    0
Lwu_AST-A4  EYELITEDDLEDDEDDIDGDEDSDIDDLDEEDLVEYQEQLKRAASYGFGLGKRAGHYAFGLGKRGGSYAF  127
consensus

logo
Lva_AST_A1  GLGKKSGTYNFGLGKRSAREVVPGDFDDEQPMEFPSSSPS..SSPSSPTPSSSSPSSSSIRTKREAES..  164
Lwu_AST_A1  GLGKKSGTYNFGLGKRSAREVVPGDFDNEQSMQFSSSSPS..SSPSSPTPSSSSPPSSSVRTKREADS..  164
Lwu_AST-A3  ......................................................................    0
Mro_AST_A   GLGKKSGTYNFGLGKRSAREAIPEDTNSSQPAESSAIRTKREAVPDQEIDEEKRGQYAFGLGKREDEEKR  169
Lva_AST_A3  ......................................................................    0
Lva_AST_A2  GLGKRTPGYAFGLGKRPSNAYSFGLGKRSRSYQFGLGKRAGSYAFGLGKRAGSYAFGLGKRENPYAFGLG  210
Lwu_AST-A2  ......................................................................    0
Lwu_AST-A4  GLGKRAPGYAFGLGKRPSDAYSFGLGKRPSSYQFGLGKRTG.............................  168
consensus

logo
Lva_AST_A1  ..............................NEDVSEEKRKQYAFGLGKRGGEDDQ...............  189
Lwu_AST_A1  ..............................NEDVSEEKRKQYAFGLGKRGGEDDE...............  189
Lwu_AST-A3  ......................................................................    0
Mro_AST_A   SKAFSFGLGKRVPDDEKRDRSYSFGLGKRESESDIDKKSQQYAFGLGKRDFDQDGDVQNLDSSKAEADVE  239
Lva_AST_A3  ......................................................................    0
Lva_AST_A2  KKA.........................GHYSFGLGKRSSPYAFGLG.......................  232
Lwu_AST-A2  ........................................PYAFGLG.......................    7
Lwu_AST-A4  ........................................SYAFGLG.......................  175
consensus

logo
Lva_AST_A1  ..................KRSKSFSFGLGKRQDSDEEKRDRSYSFGLGKRDPDMDK..............  227
Lwu_AST_A1  ..................KRSKSFSFGLGKRQDSEEEKRDRSYSFGLGKRDPDVDK..............  227
Lwu_AST-A3  ......................................................................    0
Mro_AST_A   KRPRHYAFGLGKRDDYFVKRPSSYAFGLGKREDFNLDKRPKNYAFGLGKRDSDIQTRPGQYAFGLGKRNS  309
Lva_AST_A3  ..........................................YAFGLGKRDSDLEK..............   14
Lva_AST_A2  ..................KRTSPYAFGLGKRTS........PYAFGLGKRTS..................  258
Lwu_AST-A2  ..................KRTSPYAFGLGKRTS........PYAFGLGKRTS..................   33
Lwu_AST-A4  ..................KRAGSYAFGLGKRES........PYAFGLGKKAG..................  201
consensus

logo
Lva_AST_A1  .....KSQQQYAFGLGKREPGTEGDTQ...........EADLEKRRQNYAFGLGKRGEDF.IDKRALQYA  280
Lwu_AST-A1  .....KSKQQYAFGLGKREPGIESDTQ...........EADLEKRRQNYAFGLGKRGEDV.IDKRALQYA  280
Lwu_AST-A3  ........QHYAFGLGKRES..................DFDLEKR.........................   19
Mro_AST_A   DLDLSKRPQHYAFGLGKRGTDLDKRPQQYAFGLGKRESDTDLQKRPQHYAFGLGKRDSDLDLNKRPQHYA  379
Lva_AST_A3  ......RPQHYAFGLGKRDFN..................SYLDKR.........................   35
Lva_AST_A2  .........PYAFGLGKRTSP................YAFGLGKRDD.......................  280
Lwu_AST-A2  .........PYAFGLGKRTSP................YAFGLGKRDD.......................   55
Lwu_AST-A4  .........HYSFGLGKRSSP................YAFGLGKR.........................  221
consensus

logo
Lva_AST_A1  FGLGKREP..SLEKRPRNYEFGLGKRGD..NPDMDKRPQ.HYAFGLGKRG.EEDTEKRAQ..........  334
Lwu_AST-A1  FGLGKREP..SMLKRPRSYAFGLGKRGN..DLDMEKKPE.HYAFGLGKRGGEEDAEKRAQHYAFGLGKRG  345
Lwu_AST-A3  ...............PQHYAFGLGKRDS..DIDLEKKPQ.HYAFGLGKRDFEDDIEKRAHY.........   62
Mro_AST_A   FGLGKRESDFDLSKRPQHYAFGLGKRES..DLDLSKRPQ.HYAFGLGKRDLDEELEKRAQQYAFGLGKRS  446
Lva_AST_A3  ...............PQHYAFGLGKRES..NLDLGKRPQ.HYAFGLGKRESDSDLEKRPQ..........   77
Lva_AST_A2  ...............SSSSSSGLGRRSGSYSFGLGKRVPGSYAFGLGKRETNDDDDQPQEPEPTGAS...  332
Lwu_AST-A2  ...............SSSSSSGLGRRSGSYSFGLGKRVPGSYAFGLGKRETNDDDDQPQEPEPTGAS...  107
Lwu_AST-A4  ...............TSPYAFGLGKRTSPYAFGLGKRTS.PYAFGLGKRTS.........PYAFGL....  262
consensus

logo
Lva_AST_A1  ......................................................................  334
Lwu_AST-A1  ........DEDVEKRE......................................................  353
Lwu_AST-A3  ......................................................................   62
Mro_AST_A   GEYDSIFDDEDDEEYEIYDDEEGEENDTIDNEDDDTLSEYQEELKRASSYSFGLGKRAGQYAFGLGKRGG  516
Lva_AST_A3  ......................................................................   77
Lva_AST_A2  ......................................................................  332
Lwu_AST-A2  ......................................................................  107
Lwu_AST-A4  ......................................................................  262
consensus

logo
Lva_AST_A1  ......................................................................  334
Lwu_AST-A1  ......................................................................  353
Lwu_AST-A3  ......................................................................   62
Mro_AST_A   PYAFGLGKRSPYSFGLGKRPDAYSFGLGKRSRTYQFGLGKRAGPYTFGLGKRENQYAFGLGKKAGHYSFG  586
Lva_AST_A3  ......................................................................   77
Lva_AST_A2  ......................................................................  332
Lwu_AST-A2  ......................................................................  107
Lwu_AST-A4  ......................................................................  262
consensus

logo
Lva_AST_A1  ............................  334
Lwu_AST-A1  ............................  353
Lwu_AST-A3  ............................   62
Mro_AST_A   LGKRSSPYAFGLGKKSRPYAFGLGKRSS  614
Lva_AST_A3  ............................   77
Lva_AST_A2  ............................  332
Lwu_AST-A2  ............................  107
Lwu_AST-A4  ............................  262
consensus
```

AST-B

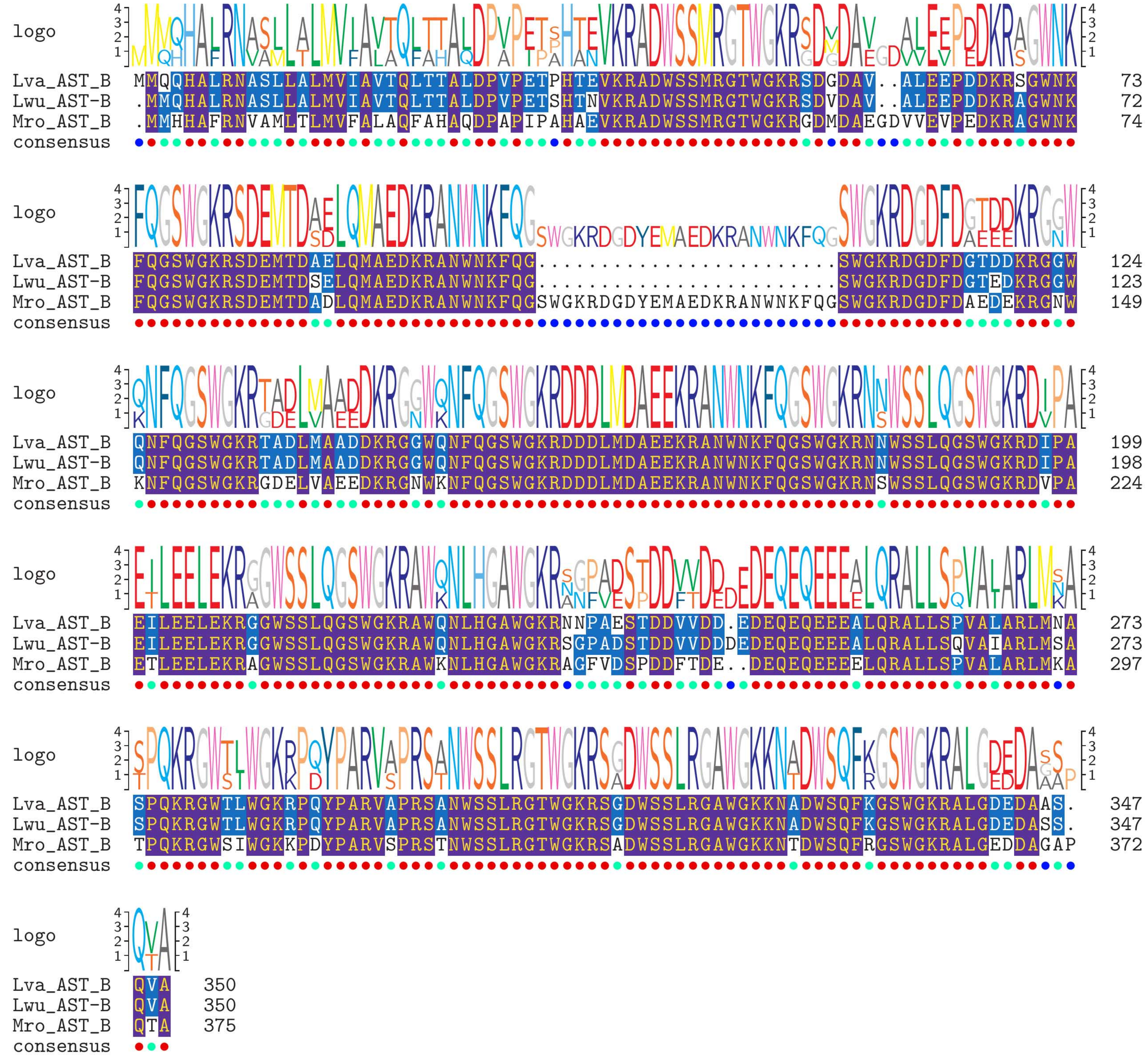
logo
Lva_AST_B MMQQHALRNASLLALMVIAVTQLTTALDPVPETPHTEVKRADWSSMRGTWGKRSDGDAV..ALEEPDDKRSGWNK 73
Lwu_AST-B .MMQHALRNASLLALMVIAVTQLTTALDPVPETSHTNVKRADWSSMRGTWGKRSDVDAV..ALEEPDDKRAGWNK 72
Mro_AST_B .MMHHAFRNVAMLTLMVFALAQFAHAQDPAPIPAHAEVKRADWSSMRGTWGKRGDMDAEGDVVEVPEDKRAGWNK 74
consensus
logo
Lva_AST_B FQGSWGKRSDEMTDAELQMAEDKRANWNKFQG........................SWGKRDGDFDGTDDKRGGW 124
Lwu_AST-B FQGSWGKRSDEMTDSELQMAEDKRANWNKFQG........................SWGKRDGDFDGTEDKRGGW 123
Mro_AST_B FQGSWGKRSDEMTDADLQMAEDKRANWNKFQGSWGKRDGDYEMAEDKRANWNKFQGSWGKRDGDFDAEDEKRGNW 149
consensus
logo
Lva_AST_B QNFQGSWGKRTADLMAADDKRGGWQNFQGSWGKRDDDLMDAEEKRANWNKFQGSWGKRNNWSSLQGSWGKRDIPA 199
Lwu_AST-B QNFQGSWGKRTADLMAADDKRGGWQNFQGSWGKRDDDLMDAEEKRANWNKFQGSWGKRNNWSSLQGSWGKRDIPA 198
Mro_AST_B KNFQGSWGKRGDELVAEEDKRGNWKNFQGSWGKRDDDLMDAEEKRANWNKFQGSWGKRNSWSSLQGSWGKRDVPA 224
consensus
logo
Lva_AST_B EILEELEKRGGWSSLQGSWGKRAWQNLHGAWGKRNNPAESTDDVVDD.EDEQEQEEEALQRALLSPVALARLMNA 273
Lwu_AST-B EILEELEKRGGWSSLQGSWGKRAWQNLHGAWGKRSGPADSTDDVVDDDEDEQEQEEEALQRALLSQVAIARLMSA 273
Mro_AST_B ETLEELEKRAGWSSLQGSWGKRAWKNLHGAWGKRAGFVDSPDDFTDE..DEQEQEEEELQRALLSPVALARLMKA 297
consensus
logo
Lva_AST_B SPQKRGWTLWGKRPQYPARVAPRSANWSSLRGTWGKRSGDWSSLRGAWGKKNADWSQFKGSWGKRALGDEDAAS. 347
Lwu_AST-B SPQKRGWTLWGKRPQYPARVAPRSANWSSLRGTWGKRSGDWSSLRGAWGKKNADWSQFKGSWGKRALGDEDASS. 347
Mro_AST_B TPQKRGWSIWGKKPDYPARVSPRSTNWSSLRGTWGKRSADWSSLRGAWGKKNTDWSQFRGSWGKRALGEDDAGAP 372
consensus
logo
Lva_AST_B QVA 350
Lwu_AST-B QVA 350
Mro_AST_B QTA 375
consensus

AST-C

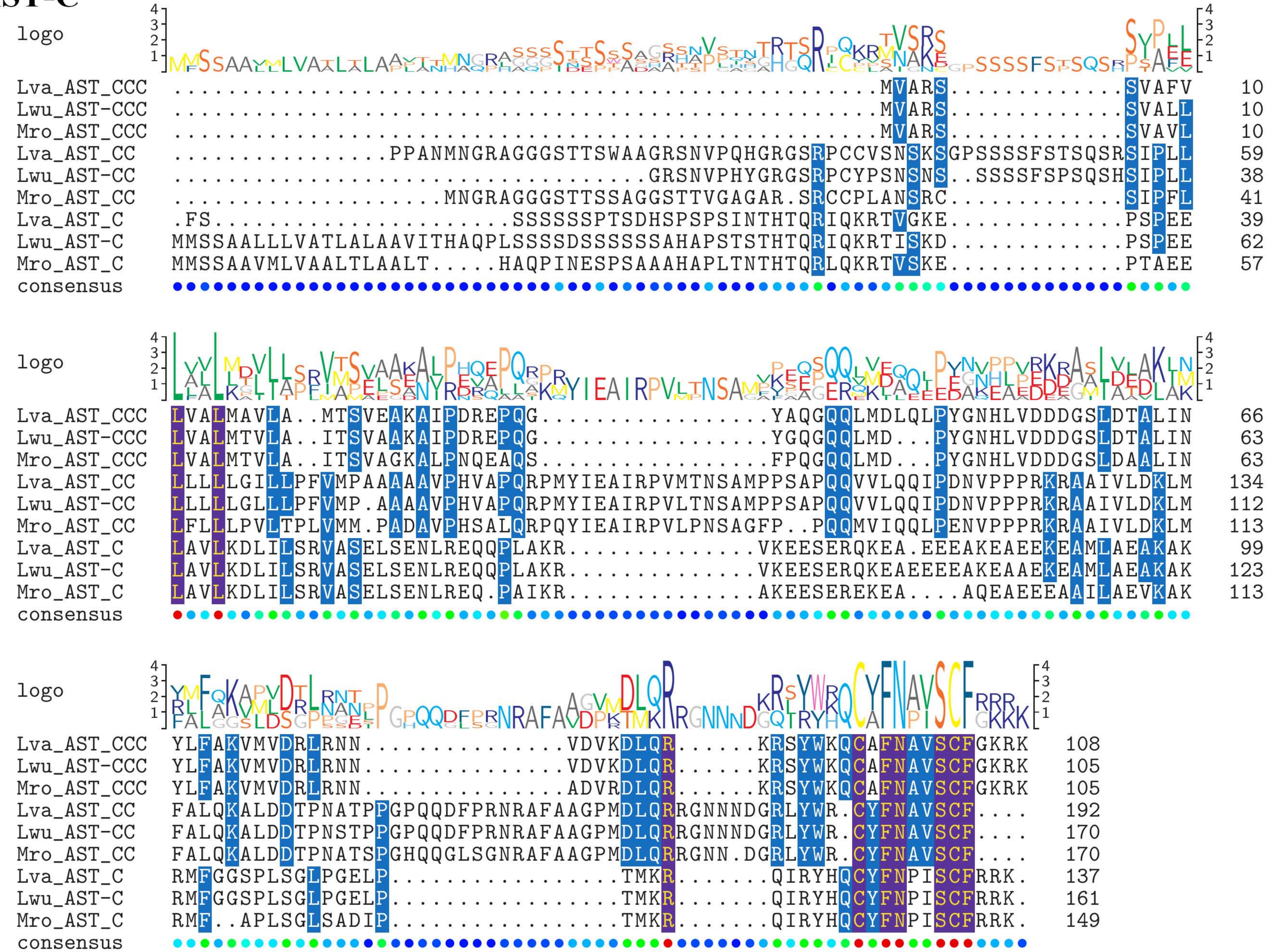
logo
Lva_AST_CCC ....MVARS.............SVAFV 10
Lwu_AST-CCC ....MVARS.............SVALL 10
Mro_AST_CCC ....MVARS.............SVAVL 10
Lva_AST_CC ................PPANMNGRAGGGSTTSWAAGRSNVPQHGRGSRPCCVSNSKSGPSSSSFSTSQSRSIPLL 59
Lwu_AST-CC ...................................GRSNVPHYGRGSRPCYPSNSNS..SSSSFSPSQSHSIPLL 38
Mro_AST_CC ....................MNGRAGGGSTTSSAGGSTTVGAGAR.SRCCPLANSRC.............SIPFL 41
Lva_AST_C .FS......................SSSSSSPTSDHSPSPSINTHTQRIQKRTVGKE.............PSPEE 39
Lwu_AST-C MMSSAALLLVATLALAAVITHAQPLSSSSDSSSSSSAHAPSTSTHTQRIQKRTISKD.............PSPEE 62
Mro_AST_C MMSSAAVMLVAALTLAALT.....HAQPINESPSAAAHAPLTNTHTQRLQKRTVSKE.............PTAEE 57
consensus
logo
Lva_AST_CCC LVALMAVLA..MTSVEAKAIPDREPQG.................YAQGQQLMDLQLPYGNHLVDDDGSLDTALIN 66
Lwu_AST-CCC LVALMTVLA..ITSVAAKAIPDREPQG.................YGQGQQLMD...PYGNHLVDDDGSLDTALIN 63
Mro_AST_CCC LVALMTVLA..ITSVAGKALPNQEAQS.................FPQGQQLMD...PYGNHLVDDDGSLDAALIN 63
Lva_AST_CC LLLLLGILLPFVMPAAAAAVPHVAPQRPMYIEAIRPVMTNSAMPPSAPQQVVLQQIPDNVPPPRKRAAIVLDKLM 134
Lwu_AST-CC LLLLLGLLLPFVMP.AAAAVPHVAPQRPMYIEAIRPVLTNSAMPPSAPQQVVLQQIPDNVPPPRKRAAIVLDKLM 112
Mro_AST_CC LFLLLPVLTPLVMM.PADAVPHSALQRPQYIEAIRPVLPNSAGFP..PQQMVIQQLPENVPPPRKRAAIVLDKLM 113
Lva_AST_C LAVLKDLILSRVASELSENLREQQPLAKR..............VKEESERQKEA.EEEAKEAEEKEAMLAEAKAK 99
Lwu_AST-C LAVLKDLILSRVASELSENLREQQPLAKR..............VKEESERQKEAEEEEAKEAAEKEAMLAEAKAK 123
Mro_AST_C LAVLKDLILSRVASELSENLREQ.PAIKR..............AKEESEREKEA....AQEAEEEAAILAEVKAK 113
consensus
logo
Lva_AST_CCC YLFAKVMVDRLRNN...............VDVKDLQR......KRSYWKQCAFNAVSCFGKRK 108
Lwu_AST-CCC YLFAKVMVDRLRNN...............VDVKDLQR......KRSYWKQCAFNAVSCFGKRK 105
Mro_AST_CCC YLFAKVMVDRLRNN...............ADVRDLQR......KRSYWKQCAFNAVSCFGKRK 105
Lva_AST_CC FALQKALDDTPNATPPGPQQDFPRNRAFAAGPMDLQRRGNNNDGRLYWR.CYFNAVSCF.... 192
Lwu_AST-CC FALQKALDDTPNSTPPGPQQDFPRNRAFAAGPMDLQRRGNNNDGRLYWR.CYFNAVSCF.... 170
Mro_AST_CC FALQKALDDTPNATSPGHQQGLSGNRAFAAGPMDLQRRGNN.DGRLYWR.CYFNAVSCF.... 170
Lva_AST_C RMFGGSPLSGLPGELP.................TMKR.......QIRYHQCYFNPISCFRRK. 137
Lwu_AST-C RMFGGSPLSGLPGELP.................TMKR.......QIRYHQCYFNPISCFRRK. 161
Mro_AST_C RMF..APLSGLSADIP.................TMKR.......QIRYHQCYFNPISCFRRK. 149
consensus

Bursicon

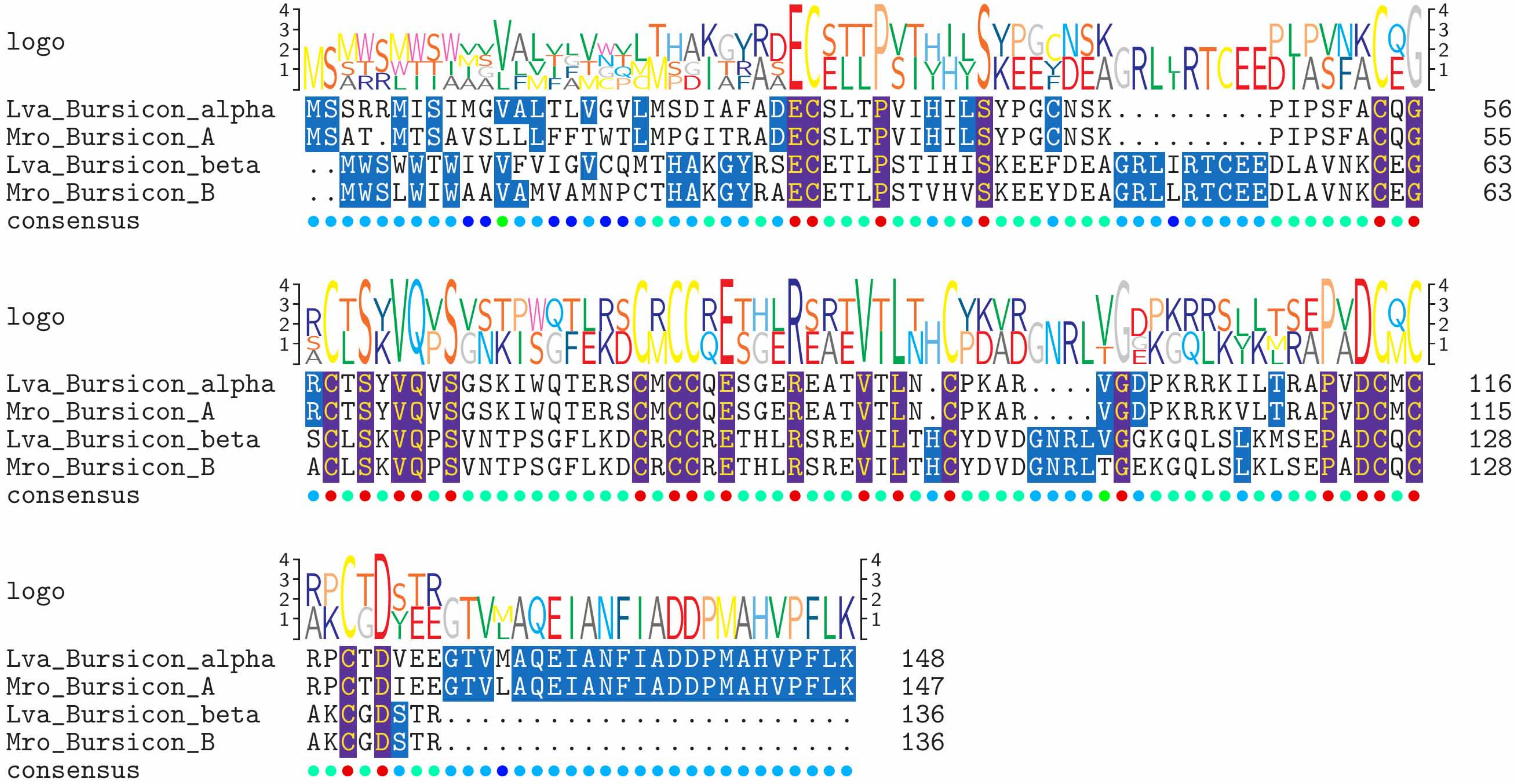
logo
Lva_Bursicon_alpha MSSRRMISIMGVALTLVGVLMSDIAFADECSLTPVIHILSYPGCNSK.........PIPSFACQG 56
Mro_Bursicon_A MSAT.MTSAVSLLLFFTWTLMPGITRADECSLTPVIHILSYPGCNSK.........PIPSFACQG 55
Lva_Bursicon_beta ..MWSWWTWIVVFVIGVCQMTHAKGYRSECETLPSTIHISKEEFDEAGRLIRTCEEDLAVNKCEG 63
Mro_Bursicon_B ..MWSLWIWAAVAMVAMNPCTHAKGYRAECETLPSTVHVSKEEYDEAGRLLRTCEEDLAVNKCEG 63
consensus
logo
Lva_Bursicon_alpha RCTSYVQVSGSKIWQTERSCMCCQESGEREATVTLN.CPKAR....VGDPKRRKILTRAPVDCMC 116
Mro_Bursicon_A RCTSYVQVSGSKIWQTERSCMCCQESGEREATVTLN.CPKAR....VGDPKRRKVLTRAPVDCMC 115
Lva_Bursicon_beta SCLSKVQPSVNTPSGFLKDCRCCRETHLRSREVILTHCYDVDGNRLVGGKGQLSLKMSEPADCQC 128
Mro_Bursicon_B ACLSKVQPSVNTPSGFLKDCRCCRETHLRSREVILTHCYDVDGNRLTGEKGQLSLKLSEPADCQC 128
consensus
logo
Lva_Bursicon_alpha RPCTDVEEGTVMAQEIANFIADDPMAHVPFLK 148
Mro_Bursicon_A RPCTDIEEGTVLAQEIANFIADDPMAHVPFLK 147
Lva_Bursicon_beta AKCGDSTR........................ 136
Mro_Bursicon_B AKCGDSTR........................ 136
consensus

Calcitonin

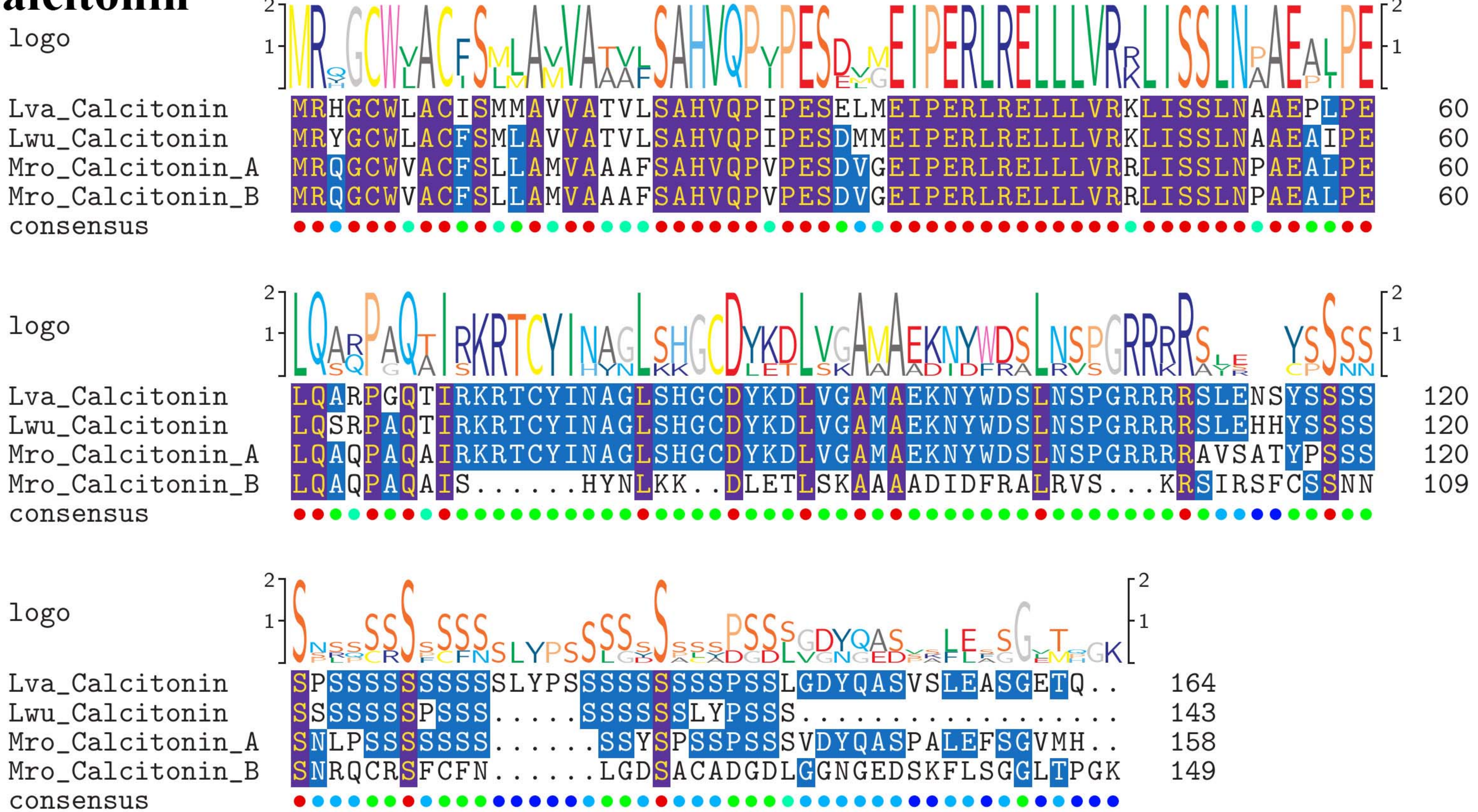

logo
Lva_Calcitonin MRHGCWLACISMMAVVATVLSAHVQPIPESELMEIPERLRELLLVRKLISSLNAAEPLPE 60
Lwu_Calcitonin MRYGCWLACFSMLAVVATVLSAHVQPIPESDMMEIPERLRELLLVRKLISSLNAAEAIPE 60
Mro_Calcitonin_A MRQGCWVACFSLLAMVAAAFSAHVQPVPESDVGEIPERLRELLLVRRLISSLNPAEALPE 60
Mro_Calcitonin_B MRQGCWVACFSLLAMVAAAFSAHVQPVPESDVGEIPERLRELLLVRRLISSLNPAEALPE 60
consensus
logo
Lva_Calcitonin LQARPGQTIRKRTCYINAGLSHGCDYKDLVGAMAEKNYWDSLNSPGRRRRSLENSYSSSS 120
Lwu_Calcitonin LQSRPAQTIRKRTCYINAGLSHGCDYKDLVGAMAEKNYWDSLNSPGRRRRSLEHHYSSSS 120
Mro_Calcitonin_A LQAQPAQAIRKRTCYINAGLSHGCDYKDLVGAMAEKNYWDSLNSPGRRRRAVSATYPSSS 120
Mro_Calcitonin_B LQAQPAQAIS......HYNLKK..DLETLSKAAAADIDFRALRVS...KRSIRSFCSSNN 109
consensus
logo
Lva_Calcitonin SPSSSSSSSSSLYPSSSSSSSSPSSLGDYQASVSLEASGETQ.. 164
Lwu_Calcitonin SSSSSSPSSS.....SSSSSSLYPSSS................. 143
Mro_Calcitonin_A SNLPSSSSSSS......SSYSPSSPSSSVDYQASPALEFSGVMH.. 158
Mro_Calcitonin_B SNRQCRSFCFN......LGDSACADGDLGGNGEDSKFLSGGLTPGK 149
consensus

CCAP

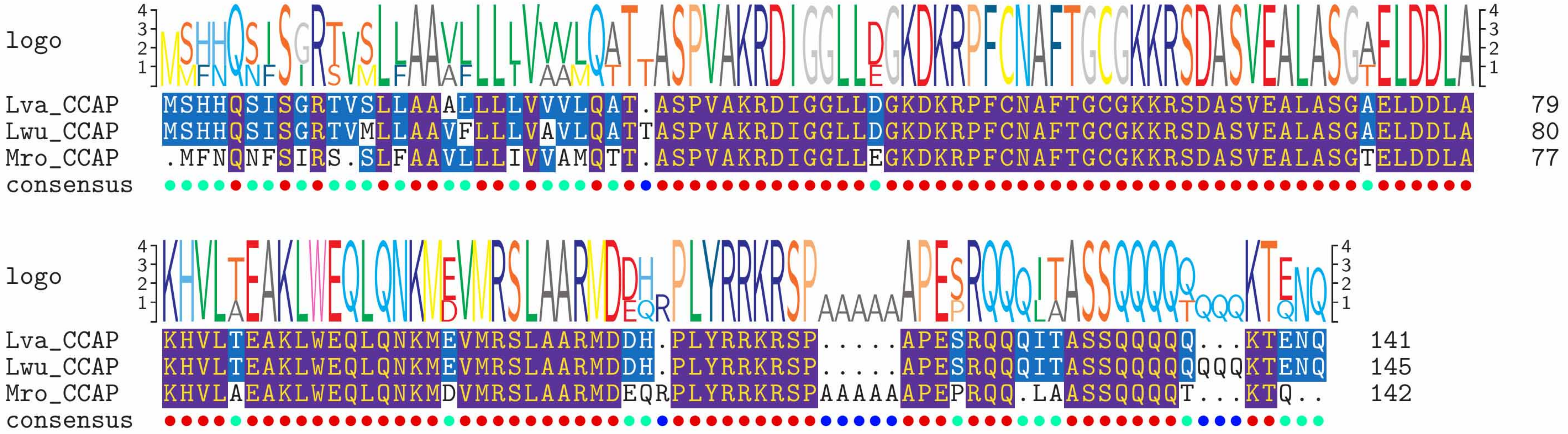
logo
Lva_CCAP MSHHQSISGRTVSLLAAALLLLVVVLQAT.ASPVAKRDIGGLLDGKDKRPFCNAFTGCGKKRSDASVEALASGAELDDLA 79
Lwu_CCAP MSHHQSISGRTVMLLAAVFLLLVAVLQATTASPVAKRDIGGLLDGKDKRPFCNAFTGCGKKRSDASVEALASGAELDDLA 80
Mro_CCAP .MFNQNFSIRS.SLFAAVLLLIVVAMQTT.ASPVAKRDIGGLLEGKDKRPFCNAFTGCGKKRSDASVEALASGTELDDLA 77
consensus
logo
Lva_CCAP KHVLTEAKLWEQLQNKMEVMRSLAARMDDH.PLYRRKRSP.....APESRQQQITASSQQQQQ...KTENQ 141
Lwu_CCAP KHVLTEAKLWEQLQNKMEVMRSLAARMDDH.PLYRRKRSP.....APESRQQQITASSQQQQQQQQKTENQ 145
Mro_CCAP KHVLAEAKLWEQLQNKMDVMRSLAARMDEQRPLYRRKRSPAAAAAAPEPRQQ.LAASSQQQQT...KTQ.. 142
consensus

CCHamide

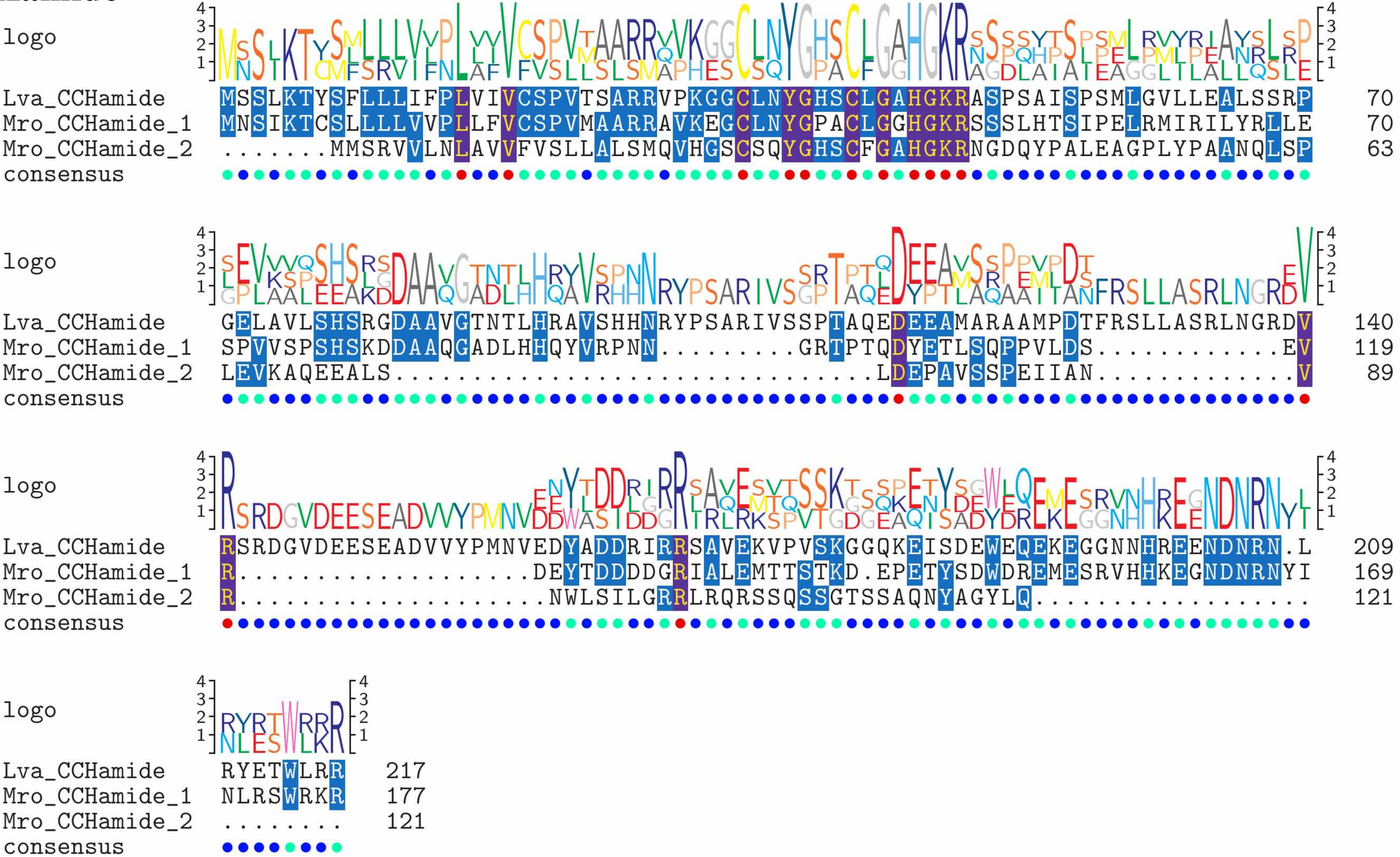
logo
Lva_CCHamide MSSLKTYSFLLLIFPLVIVCSPVTSARRVPKGGCLNYGHSCLGAHGKRASPSAISPSMLGVLLEALSSRP 70
Mro_CCHamide_1 MNSIKTCSLLLLVVPLLFVCSPVMAARRAVKEGCLNYGPACLGGHGKRSSSLHTSIPELRMIRILYRLLE 70
Mro_CCHamide_2 .......MMSRVVLNLAVVFVSLLALSMQVHGSCSQYGHSCFGAHGKRNGDQYPALEAGPLYPAANQLSP 63
consensus
logo
Lva_CCHamide GELAVLSHSRGDAAVGTNTLHRAVSHHNRYPSARIVSSPTAQEDEEAMARAAMPDTFRSLLASRLNGRDV 140
Mro_CCHamide_1 SPVVSPSHSKDDAAQGADLHHQYVRPNN.........GRTPTQDYETLSQPPVLDS............EV 119
Mro_CCHamide_2 LEVKAQEEALS...............................LDEPAVSSPEIIAN.............V 89
consensus
logo
Lva_CCHamide RSRDGVDEESEADVVYPMNVEDYADDRIRRSAVEKVPVSKGGQKEISDEWEQEKEGGNNHREENDNRN.L 209
Mro_CCHamide_1 R...................DEYTDDDDGRIALEMTTSTKD.EPETYSDWDREMESRVHHKEGNDNRNYI 169
Mro_CCHamide_2 R....................NWLSILGRRLRQRSSQSSGTSSAQNYAGYLQ.................. 121
consensus
logo
Lva_CCHamide RYETWLRR 217
Mro_CCHamide_1 NLRSWRKR 177
Mro_CCHamide_2 121
consensus

CFSH

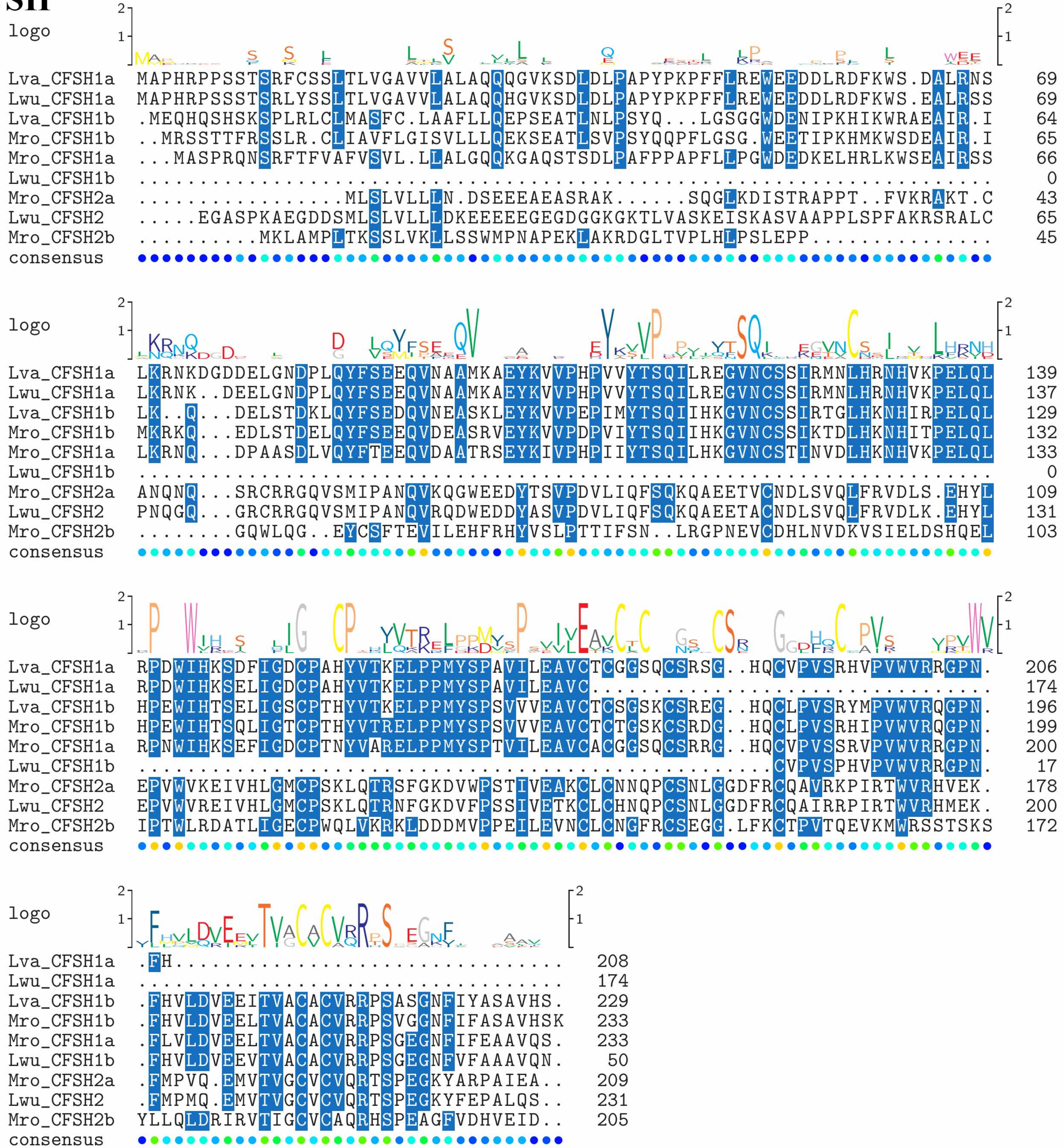
logo
Lva_CFSH1a MAPHRPPSSTSRFCSSLTLVGAVVLALAQQQGVKSDLDLPAPYPKPFFLREWEEDDLRDFKWS.DALRNS 69
Lwu_CFSH1a MAPHRPSSSTSRLYSSLTLVGAVVLALAQQHGVKSDLDLPAPYPKPFFLREWEEDDLRDFKWS.EALRSS 69
Lva_CFSH1b .MEQHQSHSKSPLRLCLMASFC.LAAFLLQEPSEATLNLPSYQ...LGSGGWDENIPKHIKWRAEAIR.I 64
Mro_CFSH1b ..MRSSTTFRSSLR.CLIAVFLGISVLLLQEKSEATLSVPSYQQPFLGSG.WEETIPKHMKWSDEAIR.I 65
Mro_CFSH1a ...MASPRQNSRFTFVAFVSVL.LLALGQQKGAQSTSDLPAFPPAPFLLPGWDEDKELHRLKWSEAIRSS 66
Lwu_CFSH1b 0
Mro_CFSH2a .................MLSLVLLLN.DSEEEAEASRAK......SQGLKDISTRAPPT..FVKRAKT.C 43
Lwu_CFSH2 .....EGASPKAEGDDSMLSLVLLLDKEEEEEGEGDGGKGKTLVASKEISKASVAAPPLSPFAKRSRALC 65
Mro_CFSH2b ..........MKLAMPLTKSSLVKLLSSWMPNAPEKLAKRDGLTVPLHLPSLEPP............... 45
consensus
logo
Lva_CFSH1a LKRNKDGDDELGNDPLQYFSEEQVNAAMKAEYKVVPHPVVYTSQILREGVNCSSIRMNLHRNHVKPELQL 139
Lwu_CFSH1a LKRNK..DEELGNDPLQYFSEEQVNAAMKAEYKVVPHPVVYTSQILREGVNCSSIRMNLHRNHVKPELQL 137
Lva_CFSH1b LK..Q...DELSTDKLQYFSEDQVNEASKLEYKVVPEPIMYTSQIIHKGVNCSSIRTGLHKNHIRPELQL 129
Mro_CFSH1b MKRKQ...EDLSTDELQYFSEEQVDEASRVEYKVVPDPVIYTSQIIHKGVNCSSIKTDLHKNHITPELQL 132
Mro_CFSH1a LKRNQ...DPAASDLVQYFTEEQVDAATRSEYKIVPHPIIYTSQILHKGVNCSTINVDLHKNHVKPELQL 133
Lwu_CFSH1b 0
Mro_CFSH2a ANQNQ...SRCRRGQVSMIPANQVKQGWEEDYTSVPDVLIQFSQKQAEETVCNDLSVQLFRVDLS.EHYL 109
Lwu_CFSH2 PNQGQ...GRCRRGQVSMIPANQVRQDWEDDYASVPDVLIQFSQKQAEETACNDLSVQLFRVDLK.EHYL 131
Mro_CFSH2b ........GQWLQG..EYCSFTEVILEHFRHYVSLPTTIFSN..LRGPNEVCDHLNVDKVSIELDSHQEL 103
consensus
logo
Lva_CFSH1a RPDWIHKSDFIGDCPAHYVTKELPPMYSPAVILEAVCTCGGSQCSRSG..HQCVPVSRHVPVWVRRGPN. 206
Lwu_CFSH1a RPDWIHKSELIGDCPAHYVTKELPPMYSPAVILEAVC................................. 174
Lva_CFSH1b HPEWIHTSELIGSCPTHYVTKELPPMYSPSVVVEAVCTCSGSKCSREG..HQCLPVSRYMPVWVRQGPN. 196
Mro_CFSH1b HPEWIHTSQLIGTCPTHYVTRELPPMYSPSVVVEAVCTCTGSKCSRDG..HQCLPVSRHIPVWVRQGPN. 199
Mro_CFSH1a RPNWIHKSEFIGDCPTNYVARELPPMYSPTVILEAVCACGGSQCSRRG..HQCVPVSSRVPVWVRRGPN. 200
Lwu_CFSH1b ....CVPVSPHVPVWVRRGPN. 17
Mro_CFSH2a EPVWVKEIVHLGMCPSKLQTRSFGKDVWPSTIVEAKCLCNNQPCSNLGGDFRCQAVRKPIRTWVRHVEK. 178
Lwu_CFSH2 EPVWVREIVHLGMCPSKLQTRNFGKDVFPSSIVETKCLCHNQPCSNLGGDFRCQAIRRPIRTWVRHMEK. 200
Mro_CFSH2b IPTWLRDATLIGECPWQLVKRKLDDDMVPPEILEVNCLCNGFRCSEGG.LFKCTPVTQEVKMWRSSTSKS 172
consensus
logo
Lva_CFSH1a .FH................................ 208
Lwu_CFSH1a 174
Lva_CFSH1b .FHVLDVEEITVACACVRRPSASGNFIYASAVHS. 229
Mro_CFSH1b .FHVLDVEELTVACACVRRPSVGGNFIFASAVHSK 233
Mro_CFSH1a .FLVLDVEELTVACACVRRPSGEGNFIFEAAVQS. 233
Lwu_CFSH1b .FHVLDVEEVTVACACVRRPSGEGNFVFAAAVQN. 50
Mro_CFSH2a .FMPVQ.EMVTVGCVCVQRTSPEGKYARPAIEA.. 209
Lwu_CFSH2 .FMPMQ.EMVTVGCVCVQRTSPEGKYFEPALQS.. 231
Mro_CFSH2b YLLQLDRIRVTIGCVCAQRHSPEAGFVDHVEID.. 205
consensus

CHH superfamily

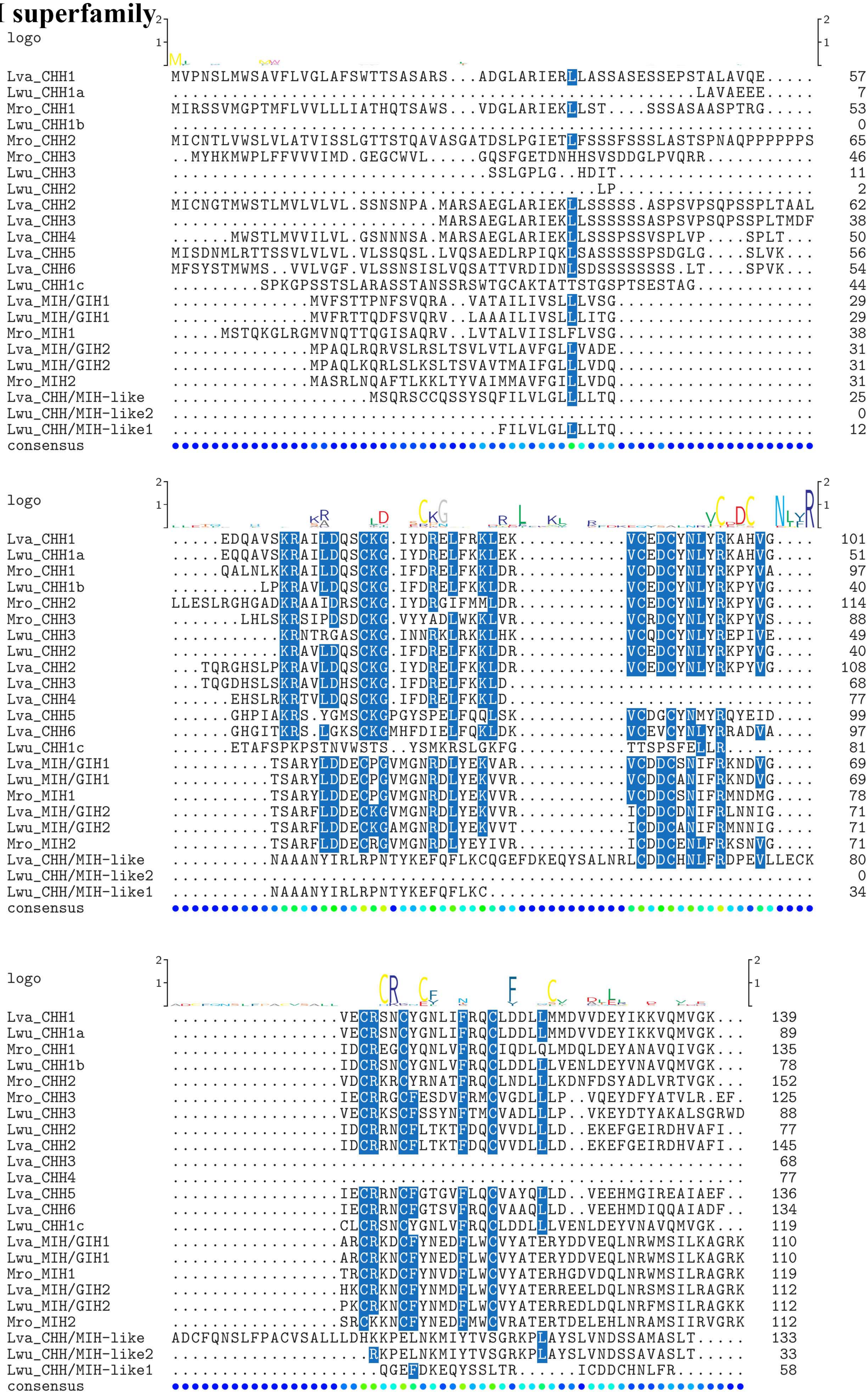

CRF-like DH44

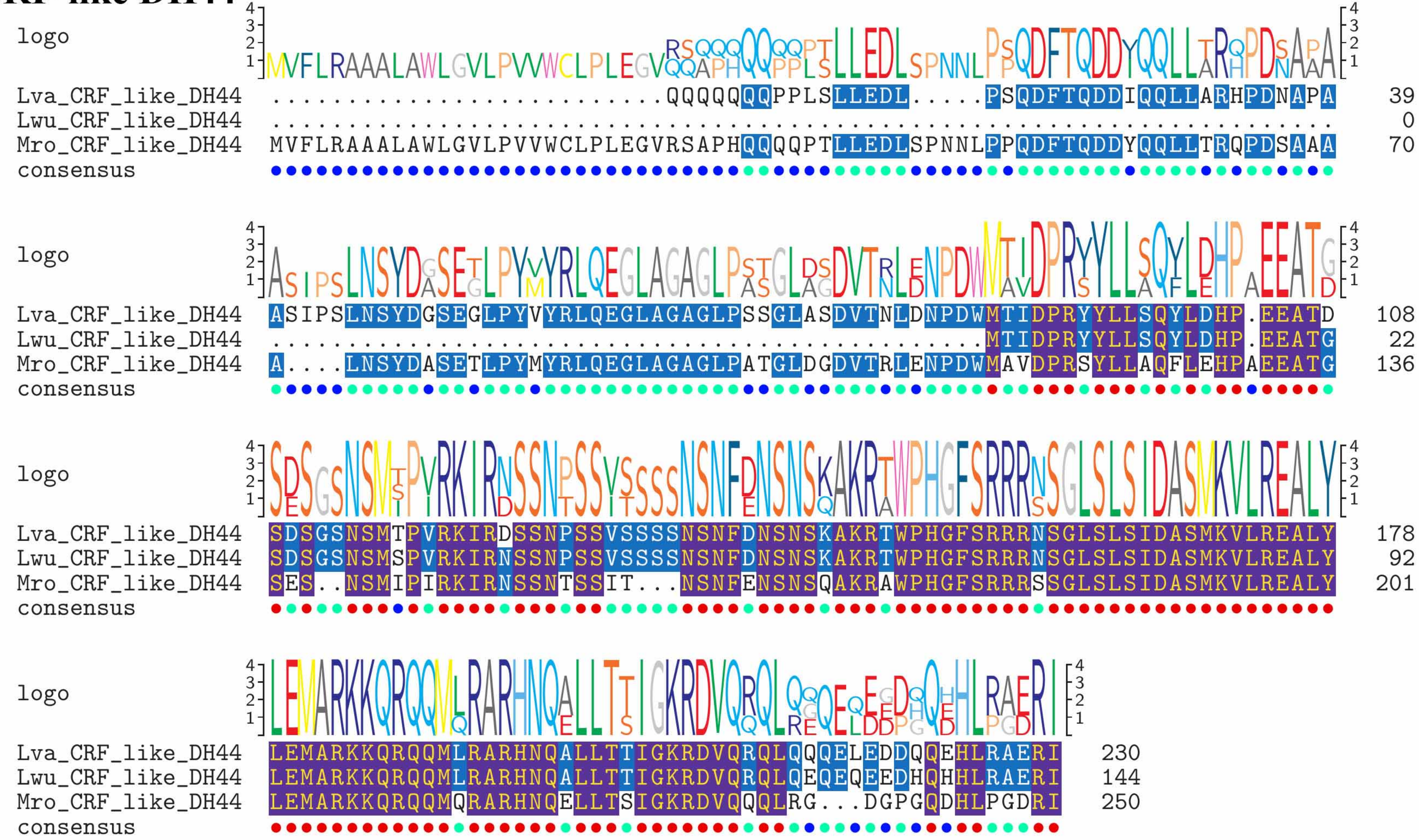

DH31

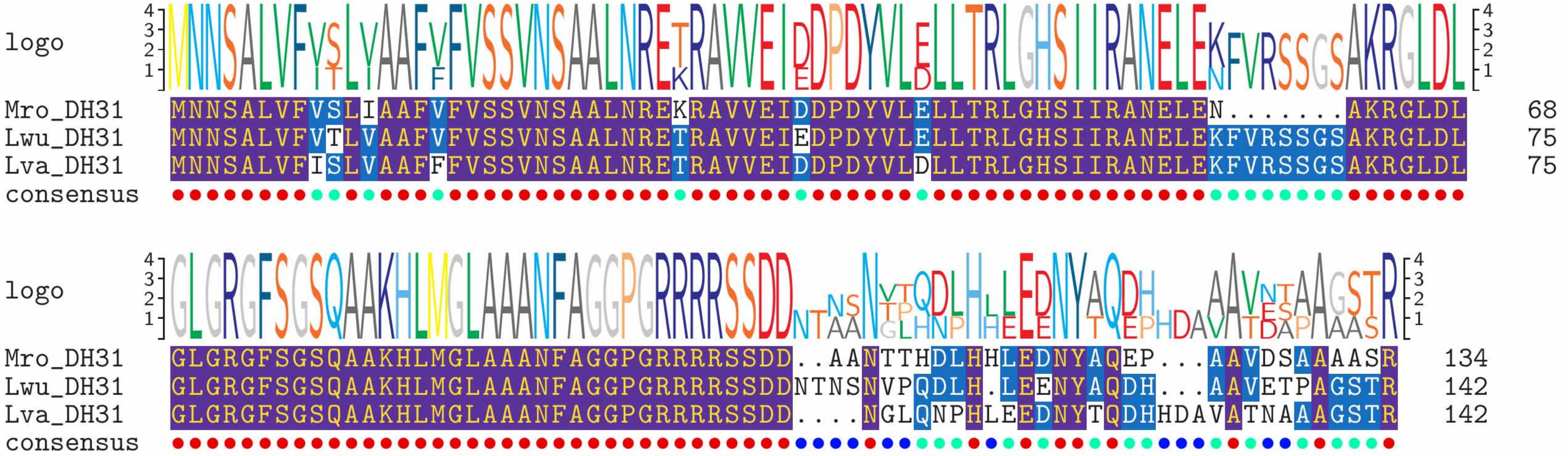
logo
Mro_DH31 MNNSALVFVSLIAAFVFVSSVNSAALNREKRAVVEIDDPDYVLELLTRLGHSIIRANELEN........AKRGLDL 68
Lwu_DH31 MNNSALVFVTLVAAFVFVSSVNSAALNRETRAVVEIEDPDYVLELLTRLGHSIIRANELEKFVRSSGSAKRGLDL 75
Lva_DH31 MNNSALVFISLVAAFFFVSSVNSAALNRETRAVVEIDDPDYVLDLLTRLGHSIIRANELEKFVRSSGSAKRGLDL 75
consensus
logo
Mro_DH31 GLGRGFSGSQAAKHLMGLAAANFAGGPGRRRRSSDD..AANTTHDLHHLEDNYAQEP...AAVDSAAAASR 134
Lwu_DH31 GLGRGFSGSQAAKHLMGLAAANFAGGPGRRRRSSDDNTNSNVPQDLH.LEENYAQDH...AAVETPAGSTR 142
Lva_DH31 GLGRGFSGSQAAKHLMGLAAANFAGGPGRRRRSSDD....NGLQNPHLEEDNYTQDHHDAVATNAAAGSTR 142
consensus

EFLamide

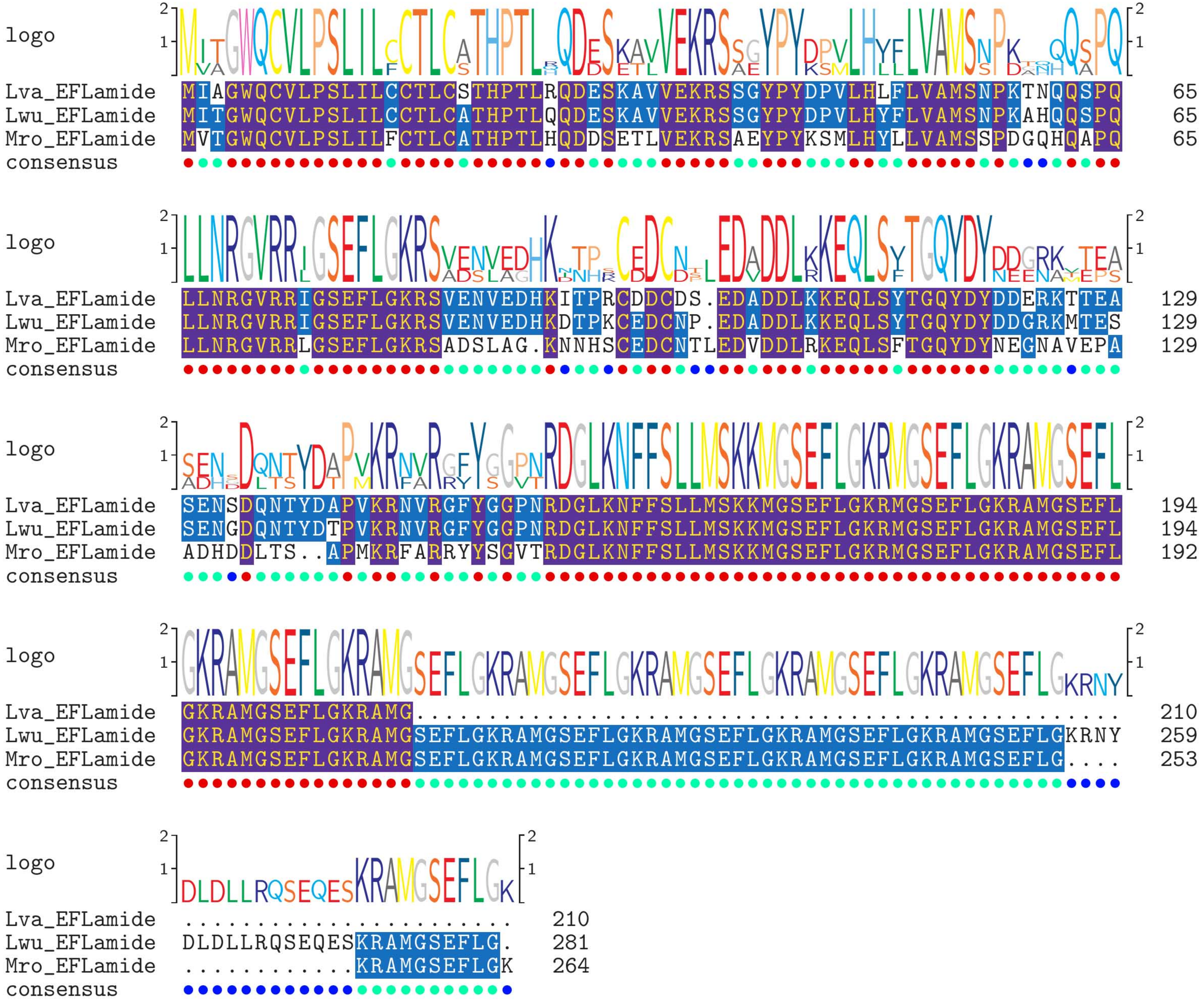

logo
Lva_EFLamide MIAGWQCVLPSLILCCTLCSTHPTLRQDESKAVVEKRSSGYPYDPVLHLFLVAMSNPKTNQQSPQ 65
Lwu_EFLamide MITGWQCVLPSLILCCTLCATHPTLQQDESKAVVEKRSSGYPYDPVLHYFLVAMSNPKAHQQSPQ 65
Mro_EFLamide MVTGWQCVLPSLILFCTLCATHPTLHQDDSETLVEKRSAEYPYKSMLHYLLVAMSSPDGQHQAPQ 65
consensus
logo
Lva_EFLamide LLNRGVRRIGSEFLGKRSVENVEDHKITPRCDDCDS.EDADDLKKEQLSYTGQYDYDDERKTTEA 129
Lwu_EFLamide LLNRGVRRIGSEFLGKRSVENVEDHKDTPKCEDCNP.EDADDLKKEQLSYTGQYDYDDGRKMTES 129
Mro_EFLamide LLNRGVRRLGSEFLGKRSADSLAG.KNNHSCEDCNTLEDVDDLRKEQLSFTGQYDYNEGNAVEPA 129
consensus
logo
Lva_EFLamide SENSDQNTYDAPVKRNVRGFYGGPNRDGLKNFFSLLMSKKMGSEFLGKRMGSEFLGKRAMGSEFL 194
Lwu_EFLamide SENGDQNTYDTPVKRNVRGFYGGPNRDGLKNFFSLLMSKKMGSEFLGKRMGSEFLGKRAMGSEFL 194
Mro_EFLamide ADHDDLTS..APMKRFARRYYSGVTRDGLKNFFSLLMSKKMGSEFLGKRMGSEFLGKRAMGSEFL 192
consensus
logo
Lva_EFLamide GKRAMGSEFLGKRAMG... 210
Lwu_EFLamide GKRAMGSEFLGKRAMGSEFLGKRAMGSEFLGKRAMGSEFLGKRAMGSEFLGKRAMGSEFLGKRNY 259
Mro_EFLamide GKRAMGSEFLGKRAMGSEFLGKRAMGSEFLGKRAMGSEFLGKRAMGSEFLGKRAMGSEFLG.... 253
consensus
logo
Lva_EFLamide 210
Lwu_EFLamide DLDLLRQSEQESKRAMGSEFLG. 281
Mro_EFLamide ....KRAMGSEFLGK 264
consensus

EH

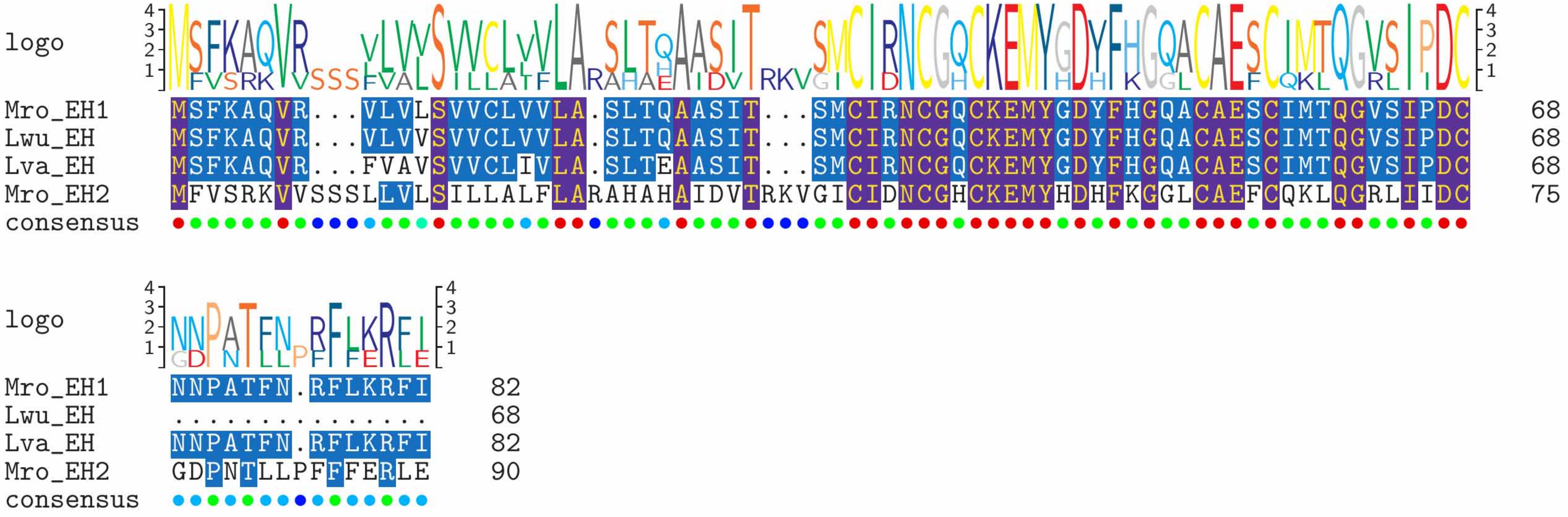
logo
Mro_EH1 MSFKAQVR...VLVLSVVCLVVLA.SLTQAASIT...SMCIRNCGQCKEMYGDYFHGQACAESCIMTQGVSIPDC 68
Lwu_EH MSFKAQVR...VLVVSVVCLVVLA.SLTQAASIT...SMCIRNCGQCKEMYGDYFHGQACAESCIMTQGVSIPDC 68
Lva_EH MSFKAQVR...FVAVSVVCLIVLA.SLTEAASIT...SMCIRNCGQCKEMYGDYFHGQACAESCIMTQGVSIPDC 68
Mro_EH2 MFVSRKVVSSSLVLSILLALFLARAHAHAIDVTRKVGICIDNCGHCKEMYHDHFKGGLCAEFCQKLQGRLIIDC 75
consensus
logo
Mro_EH1 NNPATFN.RFLKRFI 82
Lwu_EH 68
Lva_EH NNPATFN.RFLKRFI 82
Mro_EH2 GDPNTLLPFFFERLE 90
consensus

ETH

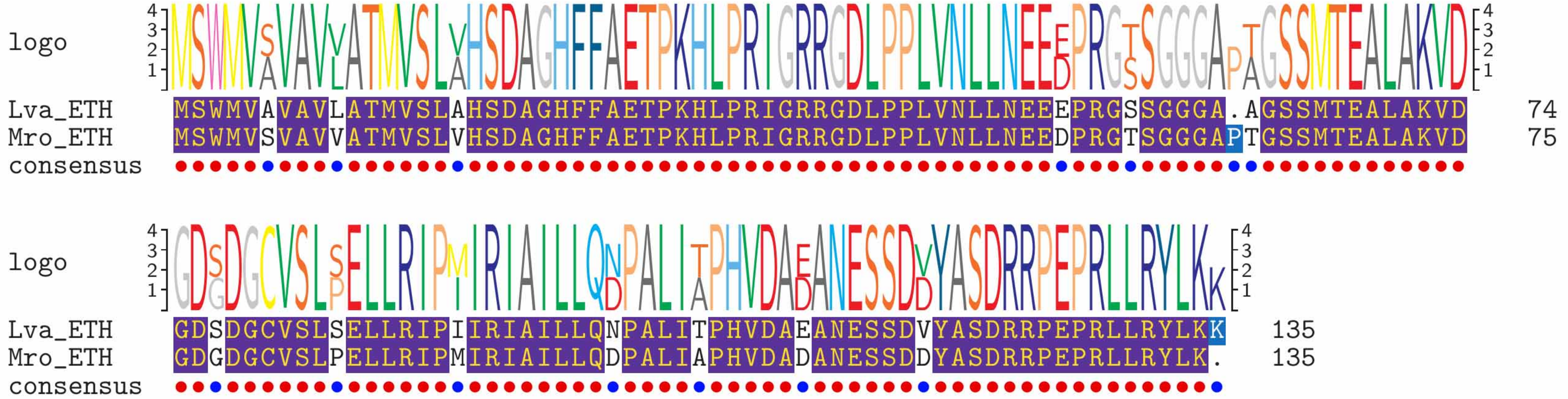
logo
Lva_ETH MSWMVAVAVLATMVSLAHSDAGHFFAETPKHLPRIGRRGDLPPLVNLLNEEEPRGSSGGGA.AGSSMTEALAKVD 74
Mro_ETH MSWMVSVAVVATMVSLVHSDAGHFFAETPKHLPRIGRRGDLPPLVNLLNEEDPRGTSGGGAPTGSSMTEALAKVD 75
consensus
logo
Lva_ETH GDSDGCVSLSELLRIPIIRIAILLQNPALITPHVDAEANESSDVYASDRRPEPRLLRYLKK 135
Mro_ETH GDGDGCVSLPELLRIPMIRIAILLQDPALIAPHVDADANESSDDYASDRRPEPRLLRYLK. 135
consensus

FLRFamide

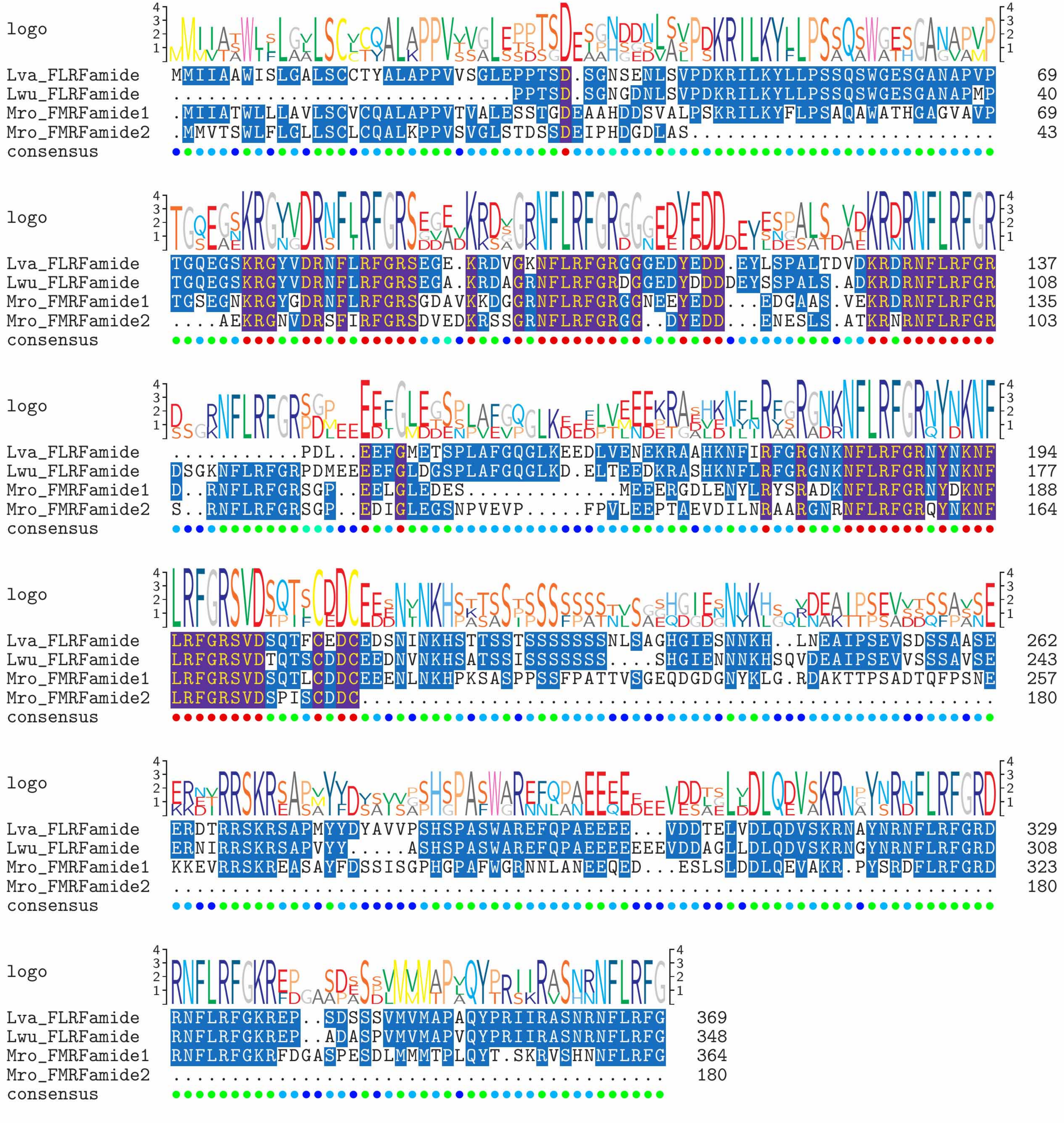
logo
Lva_FLRFamide MMIIAWISLGALSCCTYALAPPVVSGLEPPTSD.SGNSENLSVPDKRILKYLLPSSQSWGESGANAPVP 69
Lwu_FLRFamide ....PPTSD.SGNGDNLSVPDKRILKYLLPSSQSWGESGANAPMP 40
Mro_FMRFamide1 .MIIATWLLLAVLSCVCQALAPPVTVALESSTGDEAAHDDSVALPSKRILKYFLPSAQAWATHGAGVAVP 69
Mro_FMRFamide2 .MMVTSWLFLGLLSCLCQALKPPVSVGLSTDSSDEIPHDGDLAS.......................... 43
consensus
logo
Lva_FLRFamide TGQEGSKRGYVDRNFLRFGRSEGE.KRDVGKNFLRFGRGGGEDYEDD.EYLSPALTDVDKRDRNFLRFGR 137
Lwu_FLRFamide TGQEGSKRGYVDRNFLRFGRSEGA.KRDAGRNFLRFGRDGGEDYDDDDEYSSPALS.ADKRDRNFLRFGR 108
Mro_FMRFamide1 TGSEGNKRGYGDRNFLRFGRSGDAVKKDGGRNFLRFGRGGNEEYEDD...EDGAAS.VEKRDRNFLRFGR 135
Mro_FMRFamide2 ....AEKRGNVDRSFIRFGRSDVEDKRSSGRNFLRFGRGG..DYEDD...ENESLS.ATKRNRNFLRFGR 103
consensus
logo
Lva_FLRFamide ....PDL..EEFGMETSPLAFGQGLKEEDLVENEKRAAHKNFIRFGRGNKNFLRFGRNYNKNF 194
Lwu_FLRFamide DSGKNFLRFGRPDMEEEEFGLDGSPLAFGQGLKD.ELTEEDKRASHKNFLRFGRGNKNFLRFGRNYNKNF 177
Mro_FMRFamide1 D..RNFLRFGRSGP..EELGLEDES.............MEEERGDLENYLRYSRADKNFLRFGRNYDKNF 188
Mro_FMRFamide2 S..RNFLRFGRSGP..EDIGLEGSNPVEVP.....FPVLEEPTAEVDILNRAARGNRNFLRFGRQYNKNF 164
consensus
logo
Lva_FLRFamide LRFGRSVDSQTFCEDCEDSNINKHSTTSSTSSSSSSSNLSAGHGIESNNKH..LNEAIPSEVSDSSAASE 262
Lwu_FLRFamide LRFGRSVDTQTSCDDCEEDNVNKHSATSSISSSSSSS....SHGIENNNKHSQVDEAIPSEVVSSSAVSE 243
Mro_FMRFamide1 LRFGRSVDSQTLCDDCEEENLNKHPKSASPPSSFPATTVSGEQDGDGNYKLG.RDAKTTPSADTQFPSNE 257
Mro_FMRFamide2 LRFGRSVDSPISCDDC.. 180
consensus
logo
Lva_FLRFamide ERDTRRSKRSAPMYYDYAVVPSHSPASWAREFQPAEEEE...VDDTELVDLQDVSKRNAYNRNFLRFGRD 329
Lwu_FLRFamide ERNIRRSKRSAPVYY.....ASHSPASWAREFQPAEEEEEEEVDDAGLLDLQDVSKRNGYNRNFLRFGRD 308
Mro_FMRFamide1 KKEVRRSKREASAYFDSSISGPHGPAFWGRNNLANEEQED...ESLSLDDLQEVAKR.PYSRDFLRFGRD 323
Mro_FMRFamide2 180
consensus
logo
Lva_FLRFamide RNFLRFGKREP..SDSSSVMVMAPAQYPRIIRASNRNFLRFG 369
Lwu_FLRFamide RNFLRFGKREP..ADASPVMVMAPVQYPRIIRASNRNFLRFG 348
Mro_FMRFamide1 RNFLRFGKRFDGASPESDLMMMTPLQYT.SKRVSHNNFLRFG 364
Mro_FMRFamide2 180
consensus

GPA2

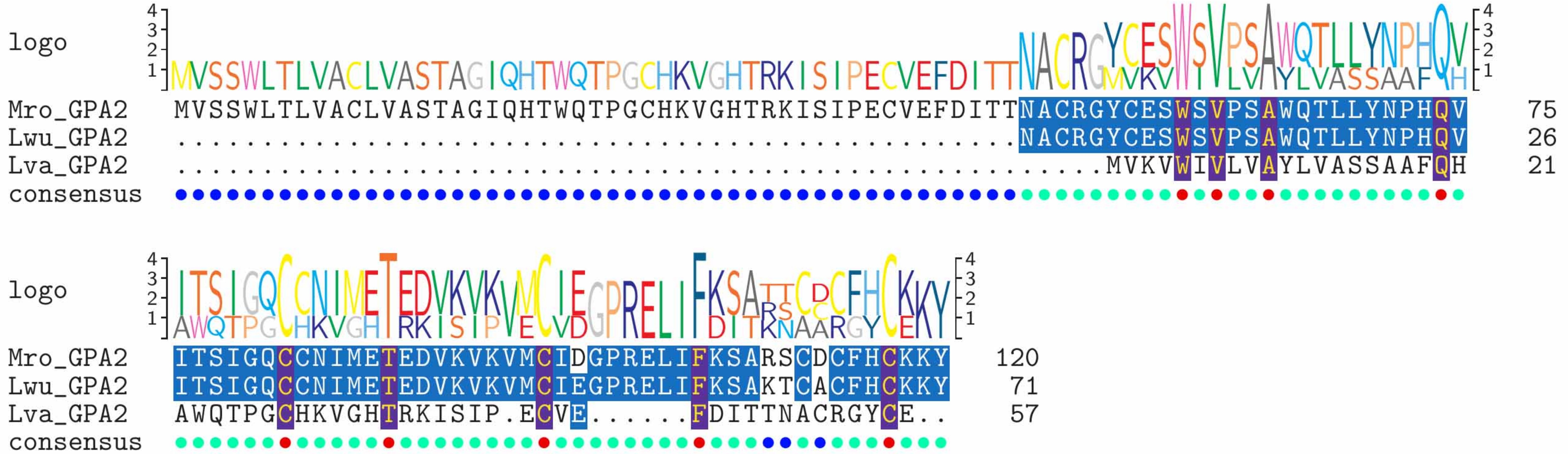
logo
Mro_GPA2 MVSSWLTLVACLVASTAGIQHTWQTPGCHKVGHTRKISIPECVEFDITTNACRGYCESWSVPSAWQTLLYNPHQV 75
Lwu_GPA2 ....NACRGYCESWSVPSAWQTLLYNPHQV 26
Lva_GPA2 ....MVKVWIVLVAYLVASSAAFQH 21
consensus
logo
Mro_GPA2 ITSIGQCCNIMETEDVKVKVMCIDGPRELIFKSARSCDCFHCKKY 120
Lwu_GPA2 ITSIGQCCNIMETEDVKVKVMCIEGPRELIFKSAKTCACFHCKKY 71
Lva_GPA2 AWQTPGCHKVGHTRKISIP.ECVE......FDITTNACRGYCE.. 57
consensus

GPB5

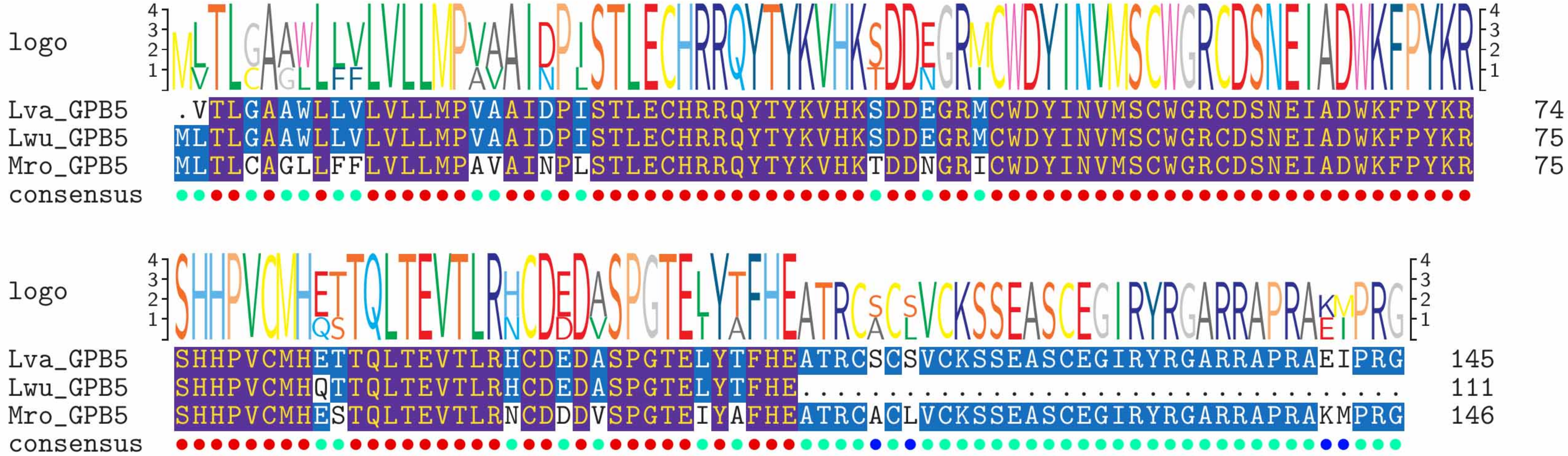
logo
Lva_GPB5 .VTLGAAWLLVLVLLMPVAAIDPISTLECHRRQYTYKVHKSDDEGRMCWDYINVMSCWGRCDSNEIADWKFPYKR 74
Lwu_GPB5 MLTLGAAWLLVLVLLMPVAAIDPISTLECHRRQYTYKVHKSDDEGRMCWDYINVMSCWGRCDSNEIADWKFPYKR 75
Mro_GPB5 MLTLCAGLLFFLVLLMPAVAINPLSTLECHRRQYTYKVHKTDDNGRICWDYINVMSCWGRCDSNEIADWKFPYKR 75
consensus
logo
Lva_GPB5 SHHPVCMHETTQLTEVTLRHCDEDASPGTELYTFHEATRCSCSVCKSSEASCEGIRYRGARRAPRAEIPRG 145
Lwu_GPB5 SHHPVCMHQTTQLTEVTLRHCDEDASPGTELYTFHE................................... 111
Mro_GPB5 SHHPVCMHESTQLTEVTLRNCDDDVSPGTEIYAFHEATRCACLVCKSSEASCEGIRYRGARRAPRAKMPRG 146
consensus

Hyrg

logo

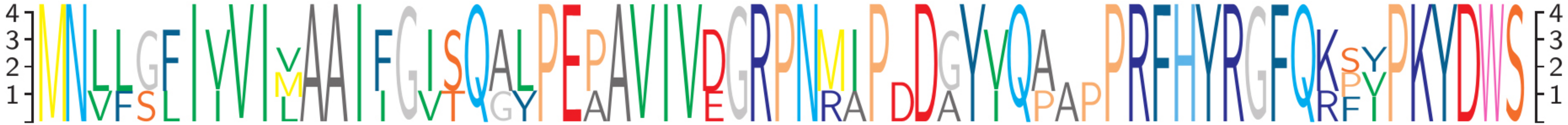

```
Lva_Hyrg1  MNLLGFIVVIMAAIFGVSQAYPEPAVIVDGRPNMIP.DGYIQA..PRFHYRGFQKPIPKYDWS  60
Lva_Hyrg2  MNVLGFIVVILAAIFGISQALPEAAVIVEGRPNRAPDDGYVQAAPPRFHYRGFQKFVPKYDWS  63
Mro_Hyrg   MNLFSLIIVIVAAIIGITQGLPEPAVIVDGRPNMIP.DAYVQP..PRFHYRGFQRSYPKYDWS  60
consensus
```

IAG

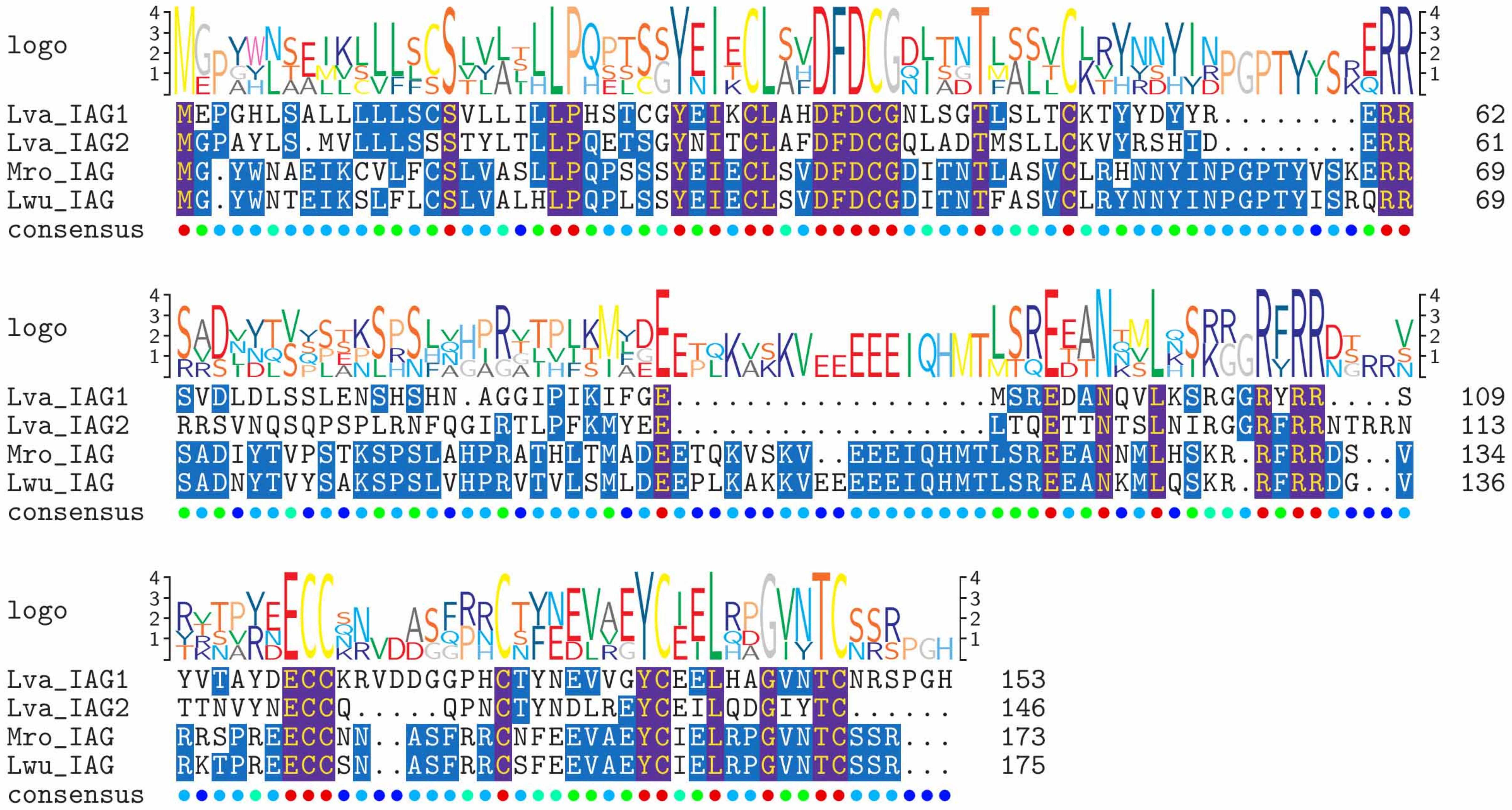
logo
Lva_IAG1 MEPGHLSALLLLLSCSVLLILLPHSTCGYEIKCLAHDFDCGNLSGTLSLTCKTYYDYYR........ERR 62
Lva_IAG2 MGPAYLS.MVLLLSSSTYLTLLPQETSGYNITCLAFDFDCGQLADTMSLLCKVYRSHID........ERR 61
Mro_IAG MG.YWNAEIKCVLFCSLVASLLPQPSSSYEIECLSVDFDCGDITNTLASVCLRHNNYINPGPTYVSKERR 69
Lwu_IAG MG.YWNTEIKSLFLCSLVALHLPQPLSSYEIECLSVDFDCGDITNTFASVCLRYNNYINPGPTYISRQRR 69
consensus
logo
Lva_IAG1 SVDLDLSSLENSHSHN.AGGIPIKIFGE..................MSREDANQVLKSRGGRYRR....S 109
Lva_IAG2 RRSVNQSQPSPLRNFQGIRTLPFKMYEE..................LTQETTNTSLNIRGGRFRRNTRRN 113
Mro_IAG SADIYTVPSTKSPSLAHPRATHLTMADEETQKVSKV..EEEIQHMTLSREEANNMLHSKR.RFRRDS..V 134
Lwu_IAG SADNYTVYSAKSPSLVHPRVTVLSMLDEEPLKAKKVEEEEEIQHMTLSREEANKMLQSKR.RFRRDG..V 136
consensus
logo
Lva_IAG1 YVTAYDECCKRVDDGGPHCTYNEVVGYCEELHAGVNTCNRSPGH 153
Lva_IAG2 TTNVYNECCQ.....QPNCTYNDLREYCEILQDGIYTC...... 146
Mro_IAG RRSPREECCNN..ASFRRCNFEEVAEYCIELRPGVNTCSSR... 173
Lwu_IAG RKTPREECCSN..ASFRRCSFEEVAEYCIELRPGVNTCSSR... 175
consensus

Kinin

```
Mro_Leucokinin MVIQRWRARSWAKLLTAGLMLCFAWAEEEDPTSYLALRYPSSLPSVEIGPEAFEDLPLILRDDLTKRNAF  70
Lwu_Kinin3     ......................................................................   0
Lva_Kinin2     ......................................................................   0
Lwu_Kinin1     ......................................................................   0
Lwu_Kinin5     ......................................................................   0
Lwu_Kinin4     ......................................................................   0
Lwu_Kinin6     ......................................................................   0
Lva_Kinin1     ......................................................................   0
Lwu_Kinin2     ......................................................................   0

Mro_Leucokinin SSWGGKRSSIKTTNGLPVALRRAYLALFRGSKPKIPTPDIELKRASFSPWGGKRSDSTLARYNTMGGSFR 140
Lwu_Kinin3     ......................................................................   0
Lva_Kinin2     ......................................................................   0
Lwu_Kinin1     ......................................................................   0
Lwu_Kinin5     ......................................................................   0
Lwu_Kinin4     ......................................................................   0
Lwu_Kinin6     ...................................PTPDIELKRAAFSPWGGKRSDPTLSRTNINGVSFR  35
Lva_Kinin1     ......................................................................   0
Lwu_Kinin2     ......................................................................   0

Mro_Leucokinin PWGGKRSPSYPPDTEDFETLESILPYLSIVPNEKMKDIAEKISKTPMFWDENQILSESDPSVYYRADVEP 210
Lwu_Kinin3     ......................................................................   0
Lva_Kinin2     ......................................................................   0
Lwu_Kinin1     ......................................................................   0
Lwu_Kinin5     ......................................................................   0
Lwu_Kinin4     ......................................................................   0
Lwu_Kinin6     PWGGKRSQLYPEELEESENAEKLLPYLSLIQTVGTPDSDE.ISQASRYWDENQFLSNNGPFNYYPADSNL 104
Lva_Kinin1     ......................................................................   0
Lwu_Kinin2     ......................................................................   0

Mro_Leucokinin PSAHHSLRYKRETSLINPEVKEHSTNESVTTENEASEVKLPEEKRTKFSPWVGKGPDINLIREVMRRWAV 280
Lwu_Kinin3     ......................................................................   0
Lva_Kinin2     ......................................................................   0
Lwu_Kinin1     ......................................................................   0
Lwu_Kinin5     ......................................................................   0
Lwu_Kinin4     ......................................................................   0
Lwu_Kinin6     PVSSPTPRRFKRDS........................................................ 118
Lva_Kinin1     ......................................................NAWAGKRADDLDKRQS  16
Lwu_Kinin2     ......................................................................   0

Mro_Leucokinin RTPKVLERRGFSAWAGKRSIPQEQSELEGKRAFSAWAGKXXXXXXXXXQAFSAWAGKRADDLDKRQAFSAW 350
Lwu_Kinin3     ......................................................GKRDDDFDKRQAFSAW  16
Lva_Kinin2     ...........................................................NYDKRQAFSAW  11
Lwu_Kinin1     ...................................................AWAGKRGDDLDKRQAFSAW  19
Lwu_Kinin5     ......................................................................   0
Lwu_Kinin4     ....................................................................AW   2
Lwu_Kinin6     ..................................................ATFSTYETKDNAKEETEATD 138
Lva_Kinin1     FSAWAGKRESDPEKRQAFNAWAGKRGTEATKRQSFSAWAGKRNDDFEKRQAFSPWAGKRDLENQHAFSTS  86
Lwu_Kinin2     ......................................................................   0

Mro_Leucokinin AGKRADDLNKRQAFSAWAGKRAGDLEKRQPFSAWAGKRADDTDKRPAFSAWAGKRAED............ 408
Lwu_Kinin3     AGKRDDYLNKRQSFSAWAGKRDNELDKGQSFSPVAGERDDTTDKRHSFSAWAGKRDDD............  74
Lva_Kinin2     AGKRNNDFDKRQGFSAWAGKRDDDLDKRQSFSAWAGKRDDDLTKRQSFSAWAGKRDDD............  69
Lwu_Kinin1     AGKRGDDLDKRQAFSAWAGKRGNNLDKRQAFSAWAGKRGDDLDKRQAFSAWAGKRGDD............  77
Lwu_Kinin5     ...LNEILKKRAAFNAWAGKRDSVSDKRQAFSPWAGKR.DSSDKRQSFSAWAGKRSAD............  54
Lwu_Kinin4     AGKRDENFDKRQAFSPWAGKRD..LETQHTFSSSSDTQTRQNEKRQAFSAWAGKRGDD............  58
Lwu_Kinin6     LQDVKPQEEKRTKFSAWAGKRPDINLLREAVRRFVRNPPKGLKRR.GFSAWAGKRSVHPEYSGLQDEYSG 207
Lva_Kinin1     SATQTRLNEKRQAFNAWAGKRYD.....ENFNN.ADNHDETLLTLHNLNKQIYNVPLR............ 138
Lwu_Kinin2     .......NEKRQAFSAWAGKRHD.....ENFND.AEKQDETLLSLHNFNKQIYDVPLR............  45

Mro_Leucokinin FDKRQAFSAWGGKR.......SEDLDKRQAFSAWAGKRSNDFEKRQAFSAWAGKRDYTLETEDKDFDKRP 471
Lwu_Kinin3     PDKRQAFSAWAGKR.......EDDLNKRQAFSPWAGKRDD.............................. 107
Lva_Kinin2     LDKRQAFNAWAGKR.......DGDLDKRQAFSPWAGKRND.............................. 102
Lwu_Kinin1     FDKRQAFNAWAGKR.......GDDLDKRQAFSAWAGKRSA.............................. 110
Lwu_Kinin5     LDKRQAF...............................................................  61
Lwu_Kinin4     LDKRQSFSAWAGKR.......DSDPEKRQAFNAWAGKRGS..............................  91
Lwu_Kinin6     LQDKRAFSAWAGKRSLLEPTETSYSGKNDSFSQIQEESSSN............................. 248
Lva_Kinin1     .KDSL................................................................. 142
Lwu_Kinin2     .KDHSPEGHW...............SRNQATSNT....................................  63

Mro_Leucokinin AFSAWAGKRSSDQDKRQAFSAWAGKRSDDF.DKRQSFSAWAGKRGLENQYNINS...LEEQIPTDEKRQA 537
Lwu_Kinin3     ...........DLDKRQAFSAWAGKRGDDL.DKRQAFSAWAGKRG......................... 140
Lva_Kinin2     ...........DLDKRQAFNAWA............................................... 114
Lwu_Kinin1     ...........DLDKRQAFSAWAGKRSDDQ.EKRQSFSAWAGKRDNNHDKRQAFSAWAGKRSNDFDKRHS 168
Lwu_Kinin5     ......................................................................  61
Lwu_Kinin4     ...........EATKRQSFSAWAGKR............................................ 106
Lwu_Kinin6     ........AHNEDQKRASFSAWAGKRDQNVEDDEHTLDNFKPTLDTN............NSNEILKKSAA 298
Lva_Kinin1     ...................................................................... 142
Lwu_Kinin2     ......................................................................  63

Mro_Leucokinin FSAWAGKRSGENFNNDNNSEVTVTLQNTNR 567
Lwu_Kinin3     .............................. 140
Lva_Kinin2     .............................. 114
Lwu_Kinin1     FSAWAGKRDDDFDKRQAFSAWAGKR..... 193
Lwu_Kinin5     ..............................  61
Lwu_Kinin4     .............................. 106
Lwu_Kinin6     FNAWAGKRDSVSDKRQAF............ 316
Lva_Kinin1     .............................. 142
Lwu_Kinin2     ..............................  63
```

Myosuppressin

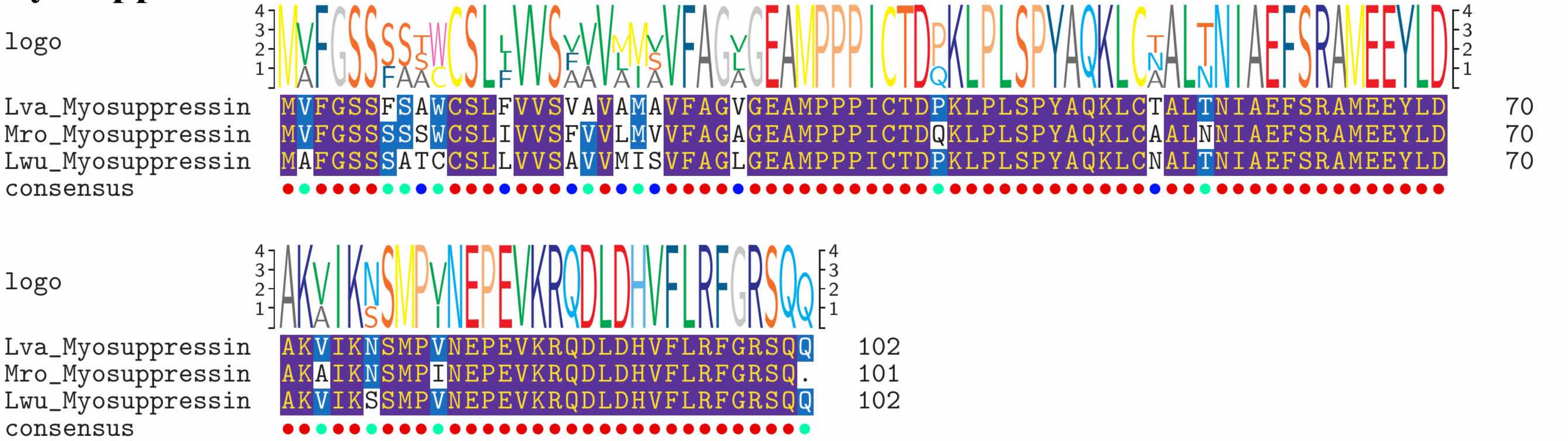

Natalisin

```
Mro_Natalisin    MSIRLWNVCGCLLLSVVTISANTQQDKQHHPKDYVREVSLRSDPFPGGDLSVLHLSKDATEANRPARSSSSSSSA   75
Lwu_Natalisin3   ...........................................................................    0
Lwu_Natalisin2   ...........................................................................    0
Lva_Natalisin    ...........................................................................    0
Lwu_Natalisin1   ...........................................................................    0

Mro_Natalisin    SSSLGIPSLSSGRNEDSFWAMRGKRPQDGGAPLGAVTRDQEMKHQRQRQNQSHLSASTANTQTDSPSAENIYAED  150
Lwu_Natalisin3   ...........................DG.....IAEKDPEGEEEEEKE...................EDIWLPE   24
Lwu_Natalisin2   ...........................................................................    0
Lva_Natalisin    ...........................................................................    0
Lwu_Natalisin1   ..................WVARGKKEDDDKTNFWVARGKKEDDDKTNFWVARGKKDDGETP..............   43

Mro_Natalisin    KPDEEKDNENGAETGESFEDEEEDEADVWFTPDDEDEDADEDEDGDLDLSEGS...WVPRDKKGISDYTPFYWGS  222
Lwu_Natalisin3   GKDE............................DVEDADAYDEYYQDVTVSKRSPPYWISRGKKSISDYIPFYWGS   71
Lwu_Natalisin2   ...........................................................................    0
Lva_Natalisin    ...........................................................................    0
Lwu_Natalisin1   ...........................FWVARGKKDTDVTPFWVTRGKKDNDQTPYWIARGKKDN.DETPYWIAR   90

Mro_Natalisin    NQNVWGKAPLSGAPIPAVLADTPRTSKTRNSNTISGWESNQSFWGKRNDEGPFWVSRGKRDHSGPFWVMRGKKGE  297
Lwu_Natalisin3   NQNVWGKAPLRGKPMQPALREVSRSTKIPRSIFSSGWESNQSFWGKR.DGGPFWLSRGRRDNTEPFWISRGKKED  145
Lwu_Natalisin2   ...........................................................................    0
Lva_Natalisin    ...........................................................................    0
Lwu_Natalisin1   GKKDNDQTPYWIARGKKDTDKTP.YWVARGKKENDQTPFWVSRGKKDNDQTPFWVARGKKEDSNPYWISRGKKED  164

Mro_Natalisin    .DYSGPYWIARGKRPLA.ADGYWGSGPFALGKRIAGAEKLEGFTDLQGNGMMGGERDDKGPFWVSRGKKE..TQD  368
Lwu_Natalisin3   PEVVGPYWIARGKRPLDNEDSSQASDPLQTGKTDQDKDDSARFSGSTG...IQKKRADNGPFWISRGKKSPSKEP  217
Lwu_Natalisin2   ...........................................................QPFWVARGKKE.....   11
Lva_Natalisin    ....NPYWISRGK........KEDSN................LYSLVS....DKEPSEINPFWVARGKRE.....   38
Lwu_Natalisin1   ...TNPYWISRGK........KEDSNPYWISR..GKKEDSHPLHSLVS....DKELSETNPFWVARGKKD.....  217

Mro_Natalisin    MDPASPFWVSRGKRT.ENNTFWVTRGKKD.GITHP.FWVNRGKKELEQNN.....PFWIARGKKAETESTWQLQD  435
Lwu_Natalisin3   EQDKSPYWVARGRRG.DNTTFWVARGKKDNSKSSS.FWVARGKKELEEKS.....PFWIARGKKAGDQPFWVARG  285
Lwu_Natalisin2   DDDKTNFWVARGKKD.DATPYWVARGKKEDGDKTN.FWVARGKKEDDDKT.....NFWVARGKKDDATPYWVARG   79
Lva_Natalisin    .GESNPYWIARGKKEPETAPYWVSRGKRTSNDASSSFWVSRGRKSDPKNSGKDDPSFWIARGRRN..........  102
Lwu_Natalisin1   .GESNPYWIARGKKEPETAPYWVSRGRRTGSDAS..FWVSRGRKSDPKDSSKDD.AFWIARGRRNFWTARGKRPN  288

Mro_Natalisin    KKAS.DATPFWVARGKKRRSFT............  456
Lwu_Natalisin3   KKEDDDKTNFWVARGKKDDATPYWV.........  310
Lwu_Natalisin2   KKEDGDKTNFWV......................   91
Lva_Natalisin    ..................................  102
Lwu_Natalisin1   SIHEKDLLMTYVMKALLEQQQQQQQQRQPSTTGD  322
```

Neuroparsin

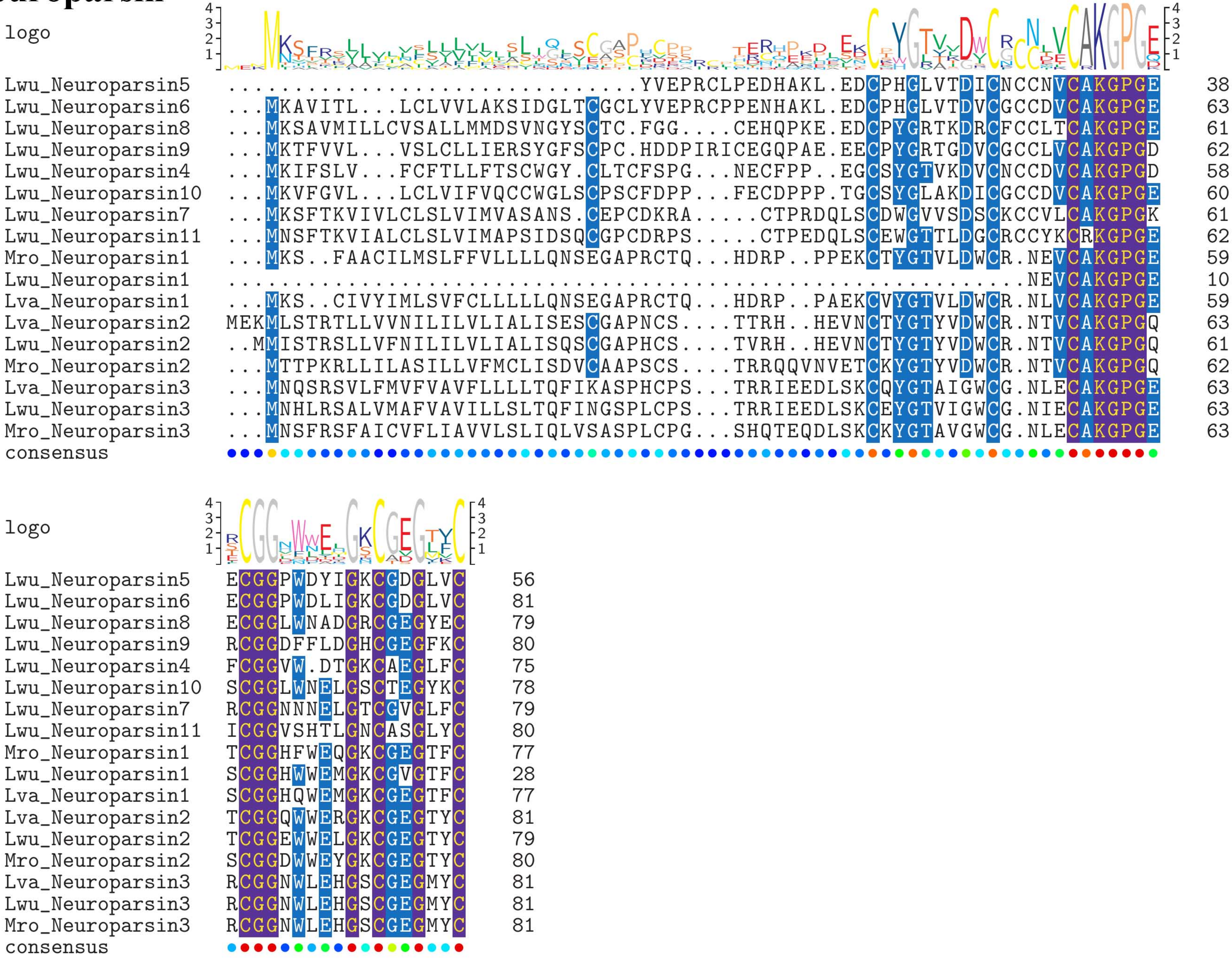

NPF

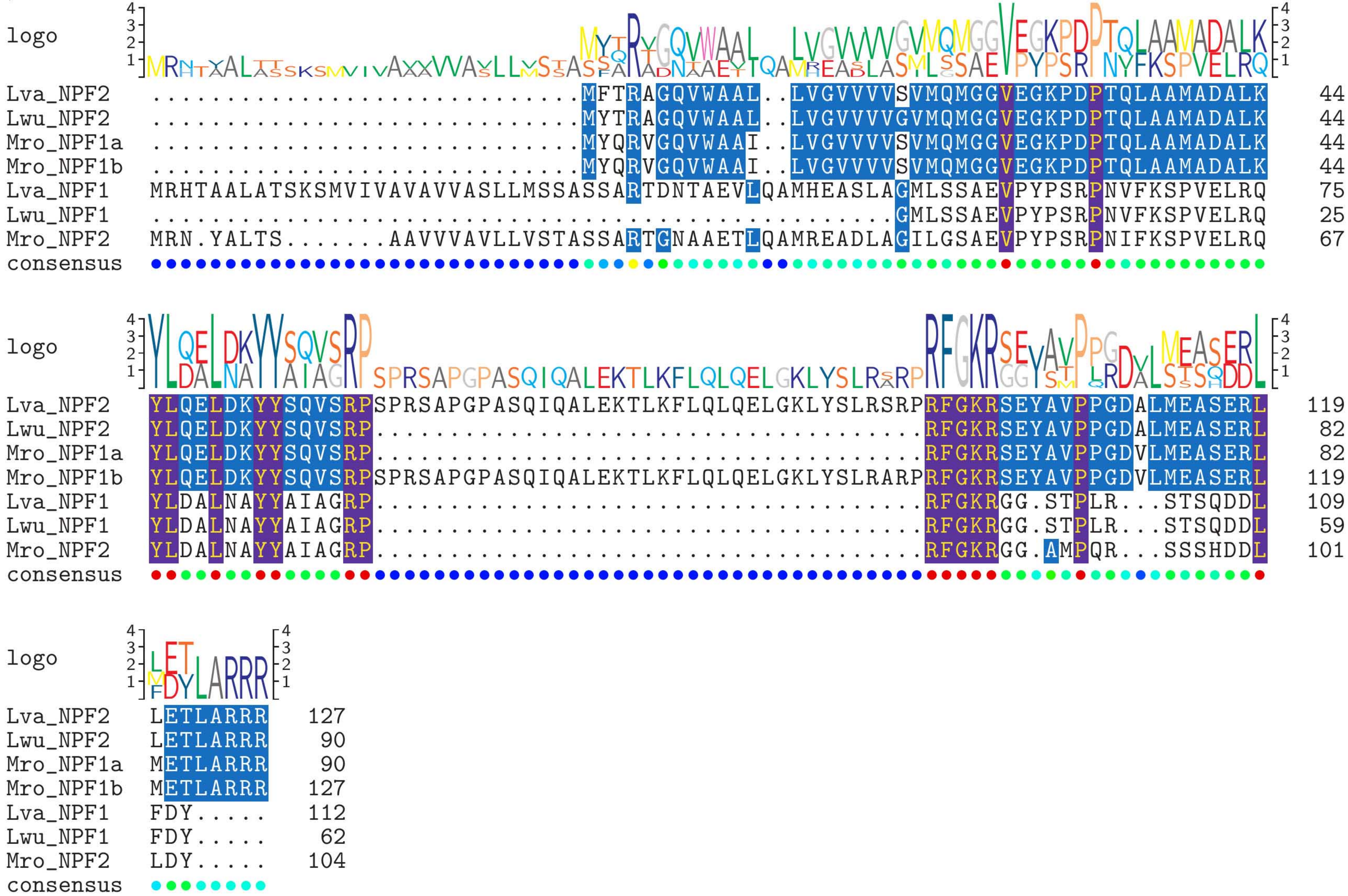
logo
Lva_NPF2 .............................MFTRAGQVWAAL..LVGVVVVSVMQMGGVEGKPDPTQLAAMADALK 44
Lwu_NPF2 .............................MYTRAGQVWAAL..LVGVVVVGVMQMGGVEGKPDPTQLAAMADALK 44
Mro_NPF1a .............................MYQRVGQVWAAI..LVGVVVVSVMQMGGVEGKPDPTQLAAMADALK 44
Mro_NPF1b .............................MYQRVGQVWAAI..LVGVVVVSVMQMGGVEGKPDPTQLAAMADALK 44
Lva_NPF1 MRHTAALATSKSMVIVAVAVVASLLMSSASSARTDNTAEVLQAMHEASLAGMLSSAEVPYPSRPNVFKSPVELRQ 75
Lwu_NPF1 ....GMLSSAEVPYPSRPNVFKSPVELRQ 25
Mro_NPF2 MRN.YALTS.......AAVVVAVLLVSTASSARTGNAAETLQAMREADLAGILGSAEVPYPSRPNIFKSPVELRQ 67
consensus
logo
Lva_NPF2 YLQELDKYYSQVSRPSPRSAPGPASQIQALEKTLKFLQLQELGKLYSLRSRPRFGKRSEYAVPPGDALMEASERL 119
Lwu_NPF2 YLQELDKYYSQVSRP.....................................RFGKRSEYAVPPGDALMEASERL 82
Mro_NPF1a YLQELDKYYSQVSRP.....................................RFGKRSEYAVPPGDVLMEASERL 82
Mro_NPF1b YLQELDKYYSQVSRPSPRSAPGPASQIQALEKTLKFLQLQELGKLYSLRARPRFGKRSEYAVPPGDVLMEASERL 119
Lva_NPF1 YLDALNAYYAIAGRP.....................................RFGKRGG.STPLR...STSQDDL 109
Lwu_NPF1 YLDALNAYYAIAGRP.....................................RFGKRGG.STPLR...STSQDDL 59
Mro_NPF2 YLDALNAYYAIAGRP.....................................RFGKRGG.AMPQR...SSSHDDL 101
consensus
logo
Lva_NPF2 LETLARRR 127
Lwu_NPF2 LETLARRR 90
Mro_NPF1a METLARRR 90
Mro_NPF1b METLARRR 127
Lva_NPF1 FDY..... 112
Lwu_NPF1 FDY..... 62
Mro_NPF2 LDY..... 104
consensus

Orcokinin

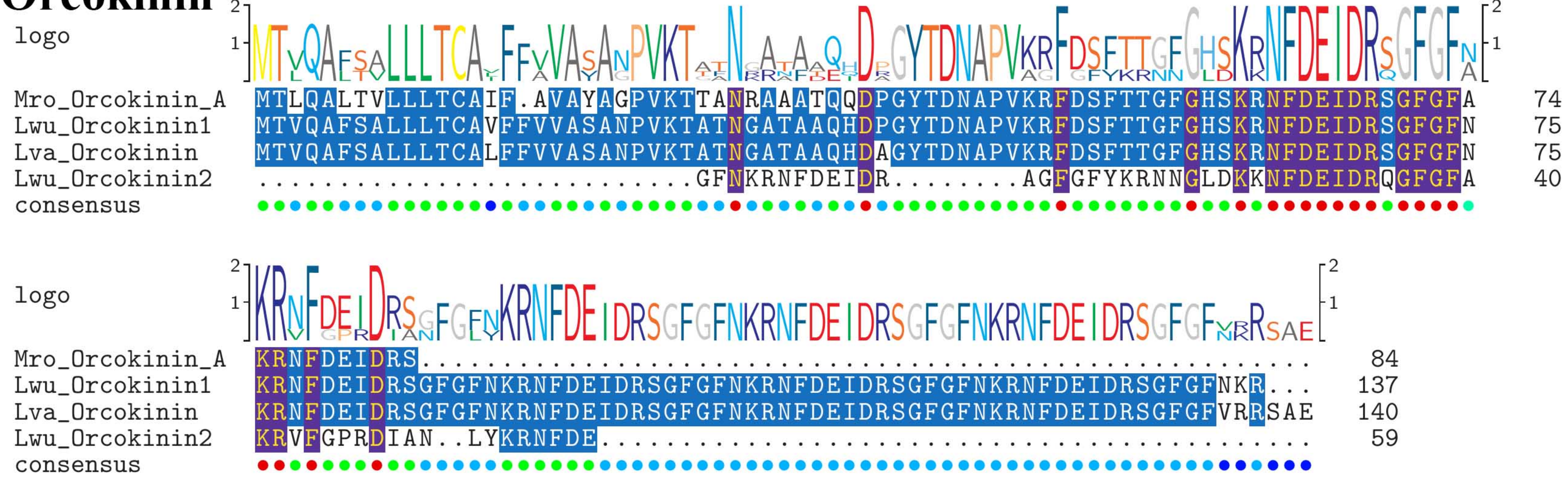
logo
Mro_Orcokinin_A MTLQALTVLLLTCAIF.AVAYAGPVKTTANRAAATQQDPGYTDNAPVKRFDSFTTGFGHSKRNFDEIDRSGFGFA 74
Lwu_Orcokinin1 MTVQAFSALLLTCAVFFVVASANPVKTATNGATAAQHDPGYTDNAPVKRFDSFTTGFGHSKRNFDEIDRSGFGFN 75
Lva_Orcokinin MTVQAFSALLLTCALFFVVASANPVKTATNGATAAQHDAGYTDNAPVKRFDSFTTGFGHSKRNFDEIDRSGFGFN 75
Lwu_Orcokinin2 ...........................GFNKRNFDEIDR........AGFGFYKRNNGLDKKNFDEIDRQGFGFA 40
consensus
logo
Mro_Orcokinin_A KRNFDEIDRS... 84
Lwu_Orcokinin1 KRNFDEIDRSGFGFNKRNFDEIDRSGFGFNKRNFDEIDRSGFGFNKRNFDEIDRSGFGFNKR... 137
Lva_Orcokinin KRNFDEIDRSGFGFNKRNFDEIDRSGFGFNKRNFDEIDRSGFGFNKRNFDEIDRSGFGFVRRSAE 140
Lwu_Orcokinin2 KRVFGPRDIAN..LYKRNFDE.. 59
consensus

PDH

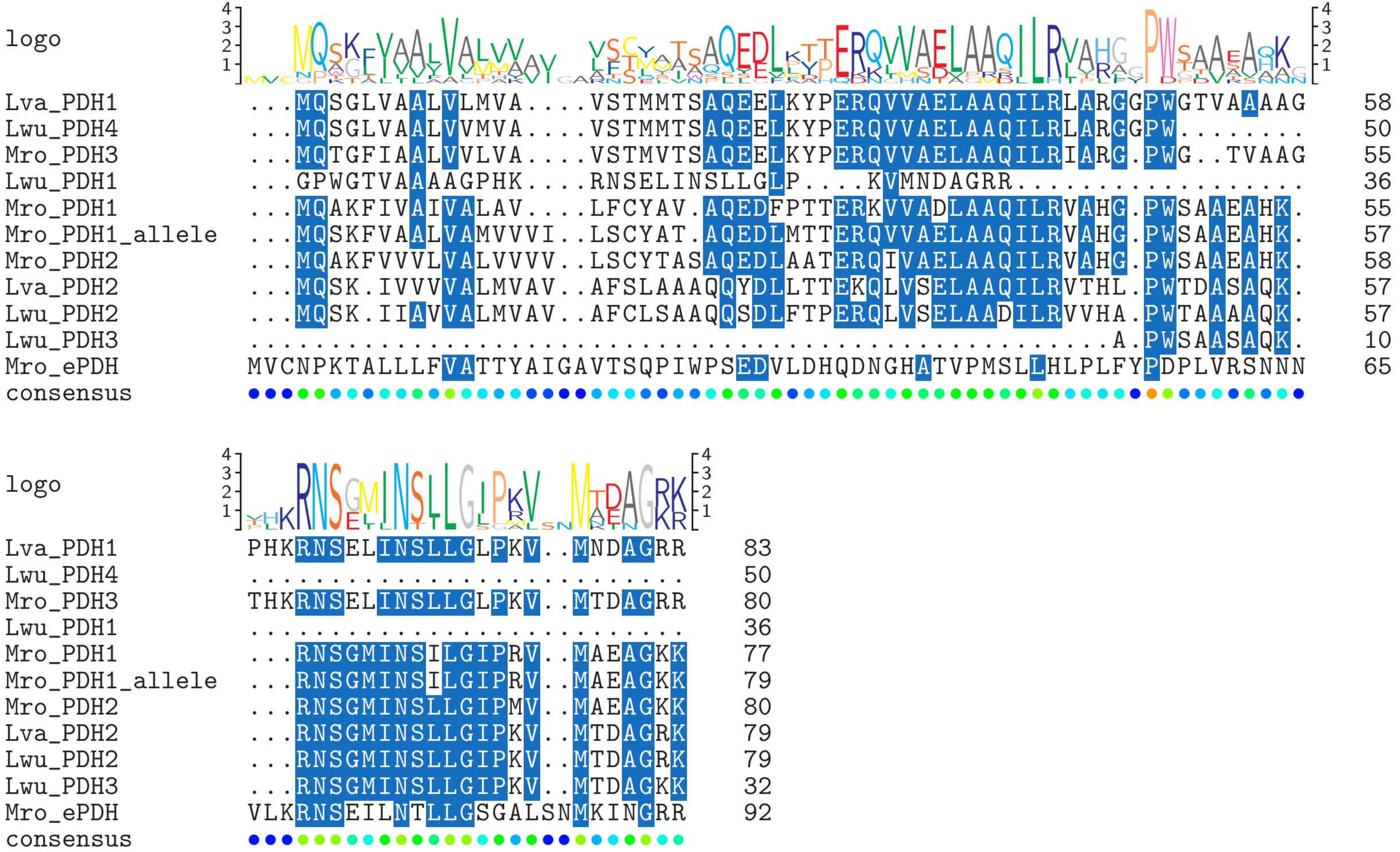
logo
Lva_PDH1 ...MQSGLVAALVLMVA....VSTMMTSAQEELKYPERQVVAELAAQILRLARGGPWGTVAAAAG 58
Lwu_PDH4 ...MQSGLVAALVVMVA....VSTMMTSAQEELKYPERQVVAELAAQILRLARGGPW........ 50
Mro_PDH3 ...MQTGFIAALVVLVA....VSTMVTSAQEELKYPERQVVAELAAQILRIARG.PWG..TVAAG 55
Lwu_PDH1 ...GPWGTVAAAAGPHK....RNSELINSLLGLP....KVMNDAGRR.................. 36
Mro_PDH1 ...MQAKFIVAIVALAV....LFCYAV.AQEDFPTTERKVVADLAAQILRVAHG.PWSAAEAHK. 55
Mro_PDH1_allele ...MQSKFVAALVAMVVVI..LSCYAT.AQEDLMTTERQVVAELAAQILRVAHG.PWSAAEAHK. 57
Mro_PDH2 ...MQAKFVVVLVALVVVV..LSCYTASAQEDLAATERQIVAELAAQILRVAHG.PWSAAEAHK. 58
Lva_PDH2 ...MQSK.IVVVVALMVAV..AFSLAAAQQYDLLTTEKQLVSELAAQILRVTHL.PWTDASAQK. 57
Lwu_PDH2 ...MQSK.IIAVVALMVAV..AFCLSAAQQSDLFTPERQLVSELAADILRVVHA.PWTAAAAQK. 57
Lwu_PDH3 ....A.PWSAASAQK. 10
Mro_ePDH MVCNPKTALLLFVATTYAIGAVTSQPIWPSEDVLDHQDNGHATVPMSLLHLPLFYPDPLVRSNNN 65
consensus
logo
Lva_PDH1 PHKRNSELINSLLGLPKV..MNDAGRR 83
Lwu_PDH4 50
Mro_PDH3 THKRNSELINSLLGLPKV..MTDAGRR 80
Lwu_PDH1 36
Mro_PDH1 ...RNSGMINSILGIPRV..MAEAGKK 77
Mro_PDH1_allele ...RNSGMINSILGIPRV..MAEAGKK 79
Mro_PDH2 ...RNSGMINSLLGIPMV..MAEAGKK 80
Lva_PDH2 ...RNSGMINSLLGIPKV..MTDAGRK 79
Lwu_PDH2 ...RNSGMINSLLGIPKV..MTDAGRK 79
Lwu_PDH3 ...RNSGMINSLLGIPKV..MTDAGKK 32
Mro_ePDH VLKRNSEILNTLLGSGALSNMKINGRR 92
consensus

Proctolin

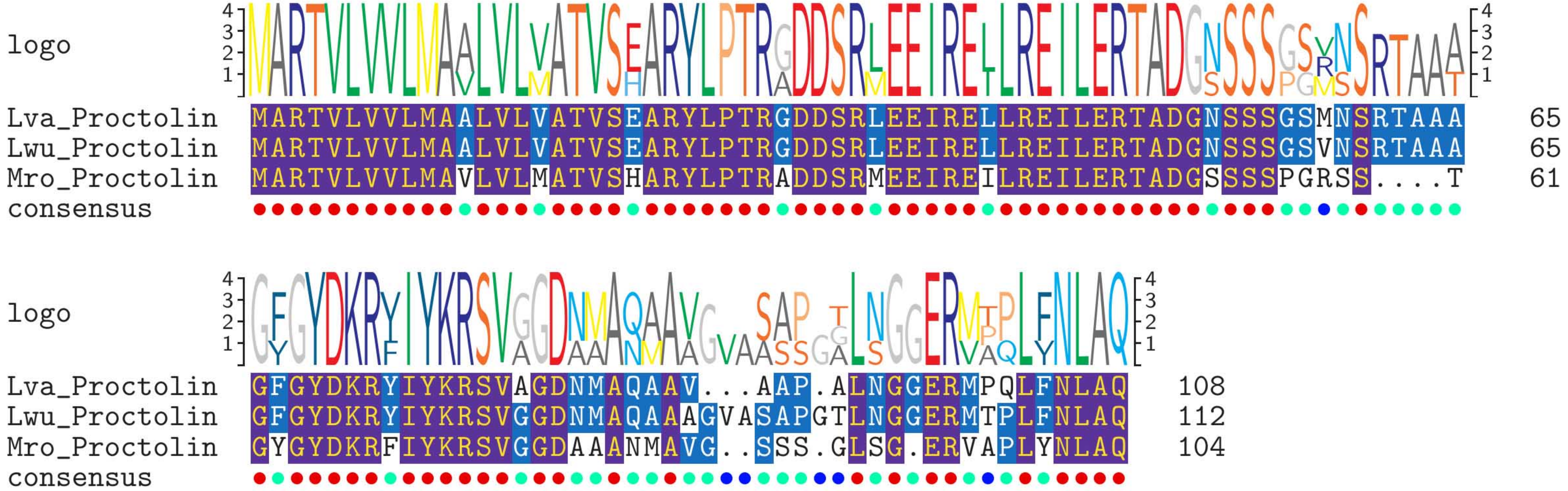
logo
Lva_Proctolin MARTVLVVLMAALVLVATVSEARYLPTRGDDSRLEEIRELLREILERTADGNSSSGSMNSRTAAA 65
Lwu_Proctolin MARTVLVVLMAALVLVATVSEARYLPTRGDDSRLEEIRELLREILERTADGNSSSGSVNSRTAAA 65
Mro_Proctolin MARTVLVVLMAVLVLMATVSHARYLPTRADDSRMEEIREILREILERTADGSSSSPGRSS....T 61
consensus
logo
Lva_Proctolin GFGYDKRYIYKRSVAGDNMAQAAV...AAP.ALNGGERMPQLFNLAQ 108
Lwu_Proctolin GFGYDKRYIYKRSVGGDNMAQAAAGVASAPGTLNGGERMTPLFNLAQ 112
Mro_Proctolin GYGYDKRFIYKRSVGGDAAANMAVG..SSS.GLSG.ERVAPLYNLAQ 104
consensus

Pyrokinin

```
logo
Lva_Pyrokinin    MLLLLITSSSSSSSSSSQPEVNSNNDVDVGSSWWWSKAAIRRSPFSPRLGKRDEAHYGLVYDDD   65
Lwu_Pyrokinin1   ................................................RLGKRDEVHYGLVYDDD   17
Mro_Pyrokinin    .................................WWWTQATVRRSPFSPRLGKRDDVQYGVLYDDD   32
Lwu_Pyrokinin2   ...................................MQAPNWIALVFICLTFAGCPSSSASLQNGG   30
consensus

logo
Lva_Pyrokinin    DDEEEDDFDTFDDDDDLNDIFEDSAAQSYSDETEVVSPQRLAVRSALVPRLGK...KAVAFSPRL  127
Lwu_Pyrokinin1   DDDEEDEFDSFEDGEDSSDVLDDLASQGYADE.EVVSPQRLAVRSALVPRLGK...KAVAFSPRL   78
Mro_Pyrokinin    YPDVDD....VSDDDSDNDLEEGFVISDYSEE..ALSPQRLSLRSALVPRLGK...RDFAFSPRL   88
Lwu_Pyrokinin2   QGDLSKPSYSVSSKGAFLEALLSYLYHIYPSS.NAVSSTNWNDQLDSADEFTQGTPKRLYYAPRA   94
consensus

logo
Lva_Pyrokinin    GKRVT.AFIPRLGKRGDFAFSPRLGKRGDFAFSPRLGKKADFAFSPRLGKRGDFAFSPRLGKKAD  191
Lwu_Pyrokinin1   GKRVT.AFIPRLGKRGDFAFSPRLGKRGDFAFSPRLGKKADFAFSPRLGKRGDFAFSPRLGKKAD  142
Mro_Pyrokinin    GKRVTTAFIPRLGKK.DFAFSPRLGKRGDFPFKPRTGKRVN.AFSPRLGKRDQTSASS...ASSS  148
Lwu_Pyrokinin2   GKRSAPAYEDVDDEK...QISEDTSKLNDGARWKRSAQEEETEEEKSRMKRDTSSSSNQPEINSN  156
consensus

logo
Lva_Pyrokinin    FAFSPRLGKRG.DFAFSPRLGKKSDFAFSPRLGKRGTAFIPRLGKRGTGASTSPSEDSWRTNAND  255
Lwu_Pyrokinin1   FAFSPRLGKRAPDFAFSPRLGKKSDFAFNPRLGKRGTAFIPRLGKRETGASTSASEDSWRTNANV  207
Mro_Pyrokinin    SSSSP.......EETWRP.........NTAQVHQRSVAFSPRLG.....................  176
Lwu_Pyrokinin2   D...........DMDLGSS.......WWWSKAAVRRSPFSPRLGKRDEVHYGLVYDDDDDDEEDE  203
consensus

logo
Lva_Pyrokinin    VSQQLQQRSVAFSPRLG  272
Lwu_Pyrokinin1   VSQQLQ...........  213
Mro_Pyrokinin    .................  176
Lwu_Pyrokinin2   FDSFEDG..........  210
consensus
```

RPCH

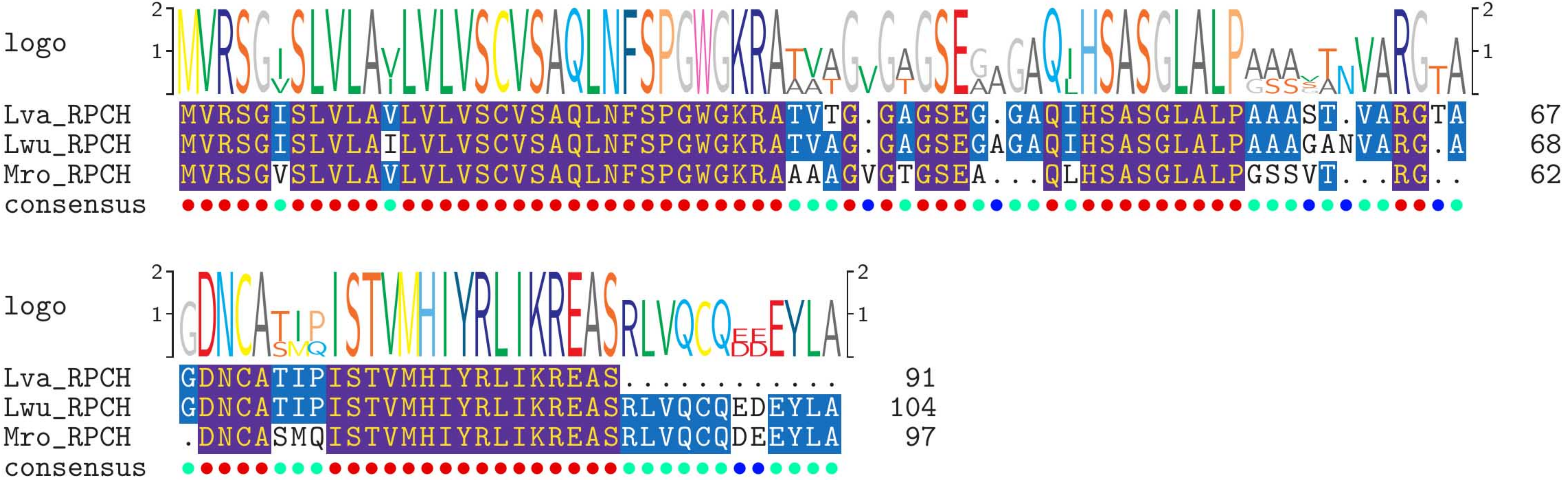

logo
Lva_RPCH MVRSGISLVLAVLVLVSCVSAQLNFSPGWGKRATVTG.GAGSEG.GAQIHSASGLALPAAAST.VARGTA 67
Lwu_RPCH MVRSGISLVLAILVLVSCVSAQLNFSPGWGKRATVAG.GAGSEGAGAQIHSASGLALPAAAGANVARG.A 68
Mro_RPCH MVRSGVSLVLAVLVLVSCVSAQLNFSPGWGKRAAAAGVGTGSEA...QLHSASGLALPGSSVT...RG.. 62
consensus
logo
Lva_RPCH GDNCATIPISTVMHIYRLIKREAS............ 91
Lwu_RPCH GDNCATIPISTVMHIYRLIKREASRLVQCQEDEYLA 104
Mro_RPCH .DNCASMQISTVMHIYRLIKREASRLVQCQDEEYLA 97
consensus

RYamide

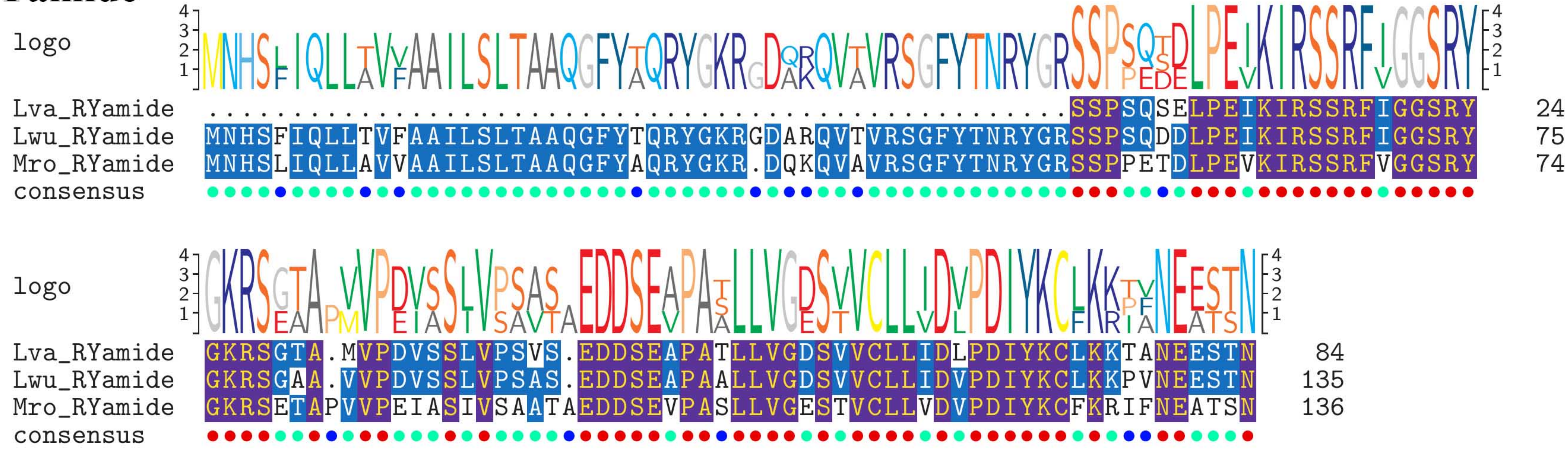
logo
Lva_RYamide ....SSPSQSELPEIKIRSSRFIGGSRY 24
Lwu_RYamide MNHSFIQLLTVFAAILSLTAAQGFYTQRYGKRGDARQVTVRSGFYTNRYGRSSPSQDDLPEIKIRSSRFIGGSRY 75
Mro_RYamide MNHSLIQLLAVVAAILSLTAAQGFYAQRYGKR.DQKQVAVRSGFYTNRYGRSSPPETDLPEVKIRSSRFVGGSRY 74
consensus
logo
Lva_RYamide GKRSGTA.MVPDVSSLVPSVS.EDDSEAPATLLVGDSVVCLLIDLPDIYKCLKKTANEESTN 84
Lwu_RYamide GKRSGAA.VVPDVSSLVPSAS.EDDSEAPAALLVGDSVVCLLIDVPDIYKCLKKPVNEESTN 135
Mro_RYamide GKRSETAPVVPEIASIVSAATAEDDSEVPASLLVGESTVCLLVDVPDIYKCFKRIFNEATSN 136
consensus

SIFamide

logo 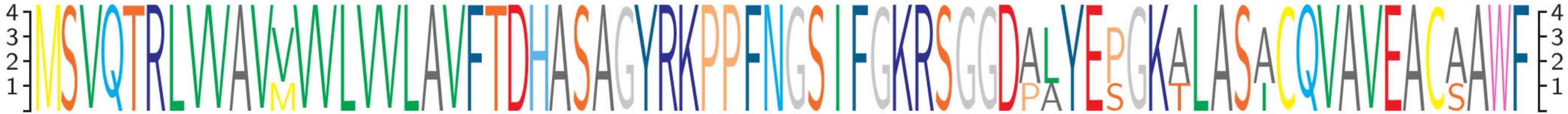

```
Lva_SIFamide  MSVQTRLVVAVVVVLVVLAVFTDHASAGYRKPPFNGSIFGKRSGGDALYEPGKALASACQVAVEACAAWF  70
Lwu_SIFamide  MSVQTRLVVAVVVVLVVLAVFTDHASAGYRKPPFNGSIFGKRSGGDALYEPGKALASACQVAVEACAAWF  70
Mro_SIFamide  MSVQTRLVVAVMVVLVVLAVFTDHASAGYRKPPFNGSIFGKRSGGDPAYESGKTLASICQVAVEACSAWF  70
consensus
```

logo 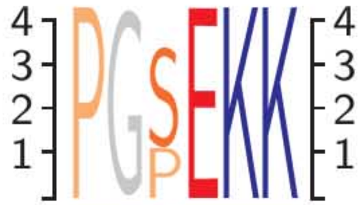

```
Lva_SIFamide  PGSEKK  76
Lwu_SIFamide  PGSEKK  76
Mro_SIFamide  PGPEKK  76
consensus
```

sNPF

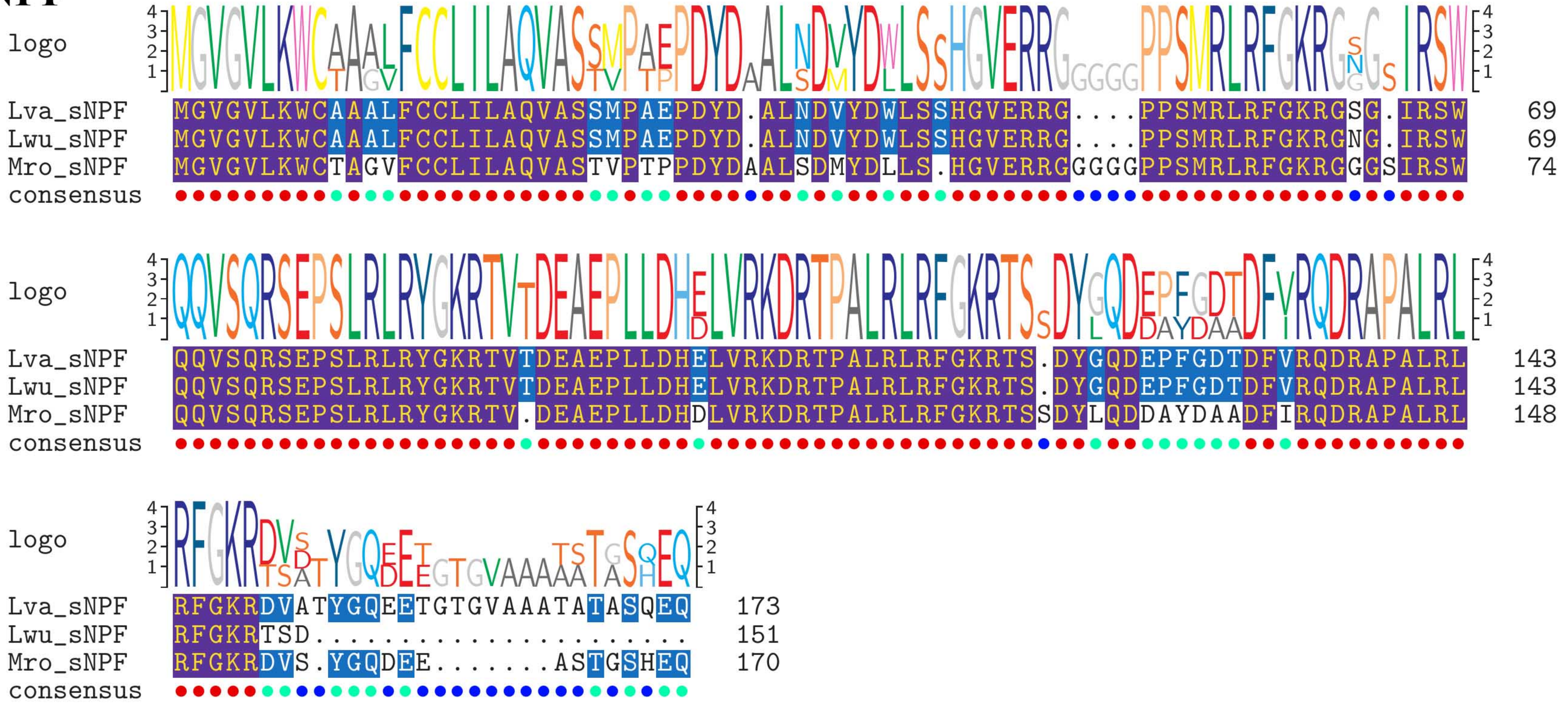

logo
Lva_sNPF MGVGVLKWCAAALFCCLILAQVASSMPAEPDYD.ALNDVYDWLSSHGVERRG....PPSMRLRFGKRGSG.IRSW 69
Lwu_sNPF MGVGVLKWCAAALFCCLILAQVASSMPAEPDYD.ALNDVYDWLSSHGVERRG....PPSMRLRFGKRGNG.IRSW 69
Mro_sNPF MGVGVLKWCTAGVFCCLILAQVASTVPTPPDYDAALSDMYDLLS.HGVERRGGGGGPPSMRLRFGKRGGGSIRSW 74
consensus
logo
Lva_sNPF QQVSQRSEPSLRLRYGKRTVTDEAEPLLDHELVRKDRTPALRLRFGKRTS.DYGQDEPFGDTDFVRQDRAPALRL 143
Lwu_sNPF QQVSQRSEPSLRLRYGKRTVTDEAEPLLDHELVRKDRTPALRLRFGKRTS.DYGQDEPFGDTDFVRQDRAPALRL 143
Mro_sNPF QQVSQRSEPSLRLRYGKRTV.DEAEPLLDHDLVRKDRTPALRLRFGKRTSSDYLQDDAYDAADFIRQDRAPALRL 148
consensus
logo
Lva_sNPF RFGKRDVATYGQEETGTGVAAATATASQEQ 173
Lwu_sNPF RFGKRTSD...................... 151
Mro_sNPF RFGKRDVS.YGQDEE.......ASTGSHEQ 170
consensus

Sulfakinin

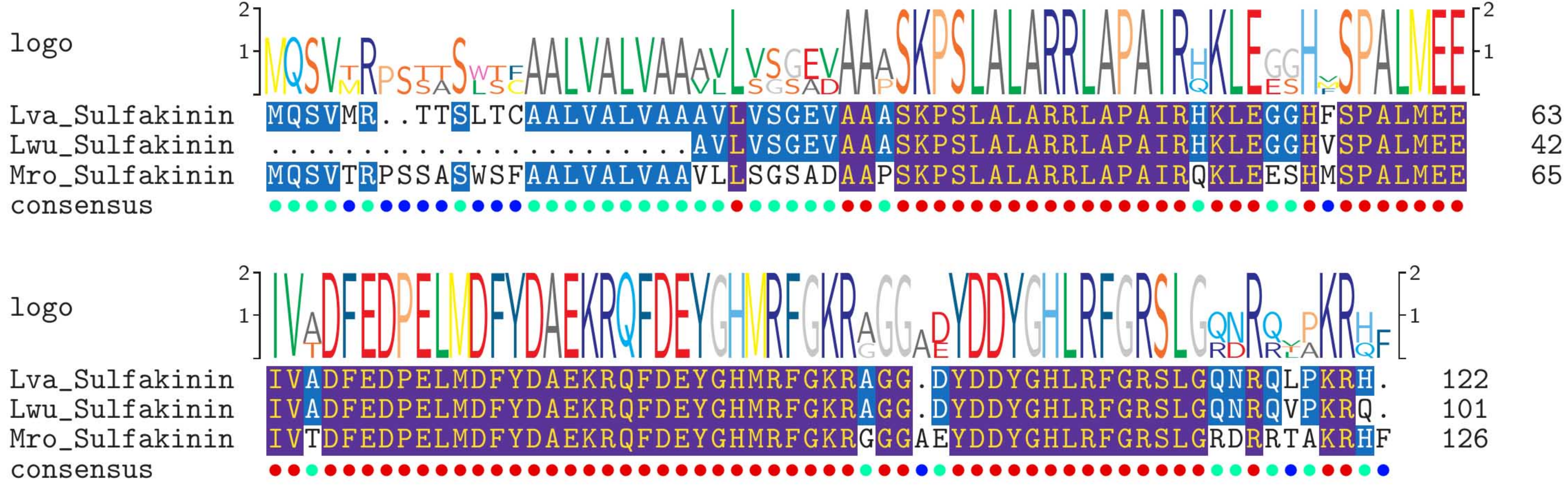
logo
Lva_Sulfakinin MQSVMR..TTSLTCAALVALVAAAVLVSGEVAAASKPSLALARRLAPAIRHKLEGGHFSPALMEE 63
Lwu_Sulfakinin ........................AVLVSGEVAAASKPSLALARRLAPAIRHKLEGGHVSPALMEE 42
Mro_Sulfakinin MQSVTRPSSASWSFAALVALVAAVLLSGSADAAPSKPSLALARRLAPAIRQKLEESHMSPALMEE 65
consensus
logo
Lva_Sulfakinin IVADFEDPELMDFYDAEKRQFDEYGHMRFGKRAGG.DYDDYGHLRFGRSLGQNRQLPKRH. 122
Lwu_Sulfakinin IVADFEDPELMDFYDAEKRQFDEYGHMRFGKRAGG.DYDDYGHLRFGRSLGQNRQVPKRQ. 101
Mro_Sulfakinin IVTDFEDPELMDFYDAEKRQFDEYGHMRFGKRGGGAEYDDYGHLRFGRSLGRDRRTAKRHF 126
consensus

Tachykinin

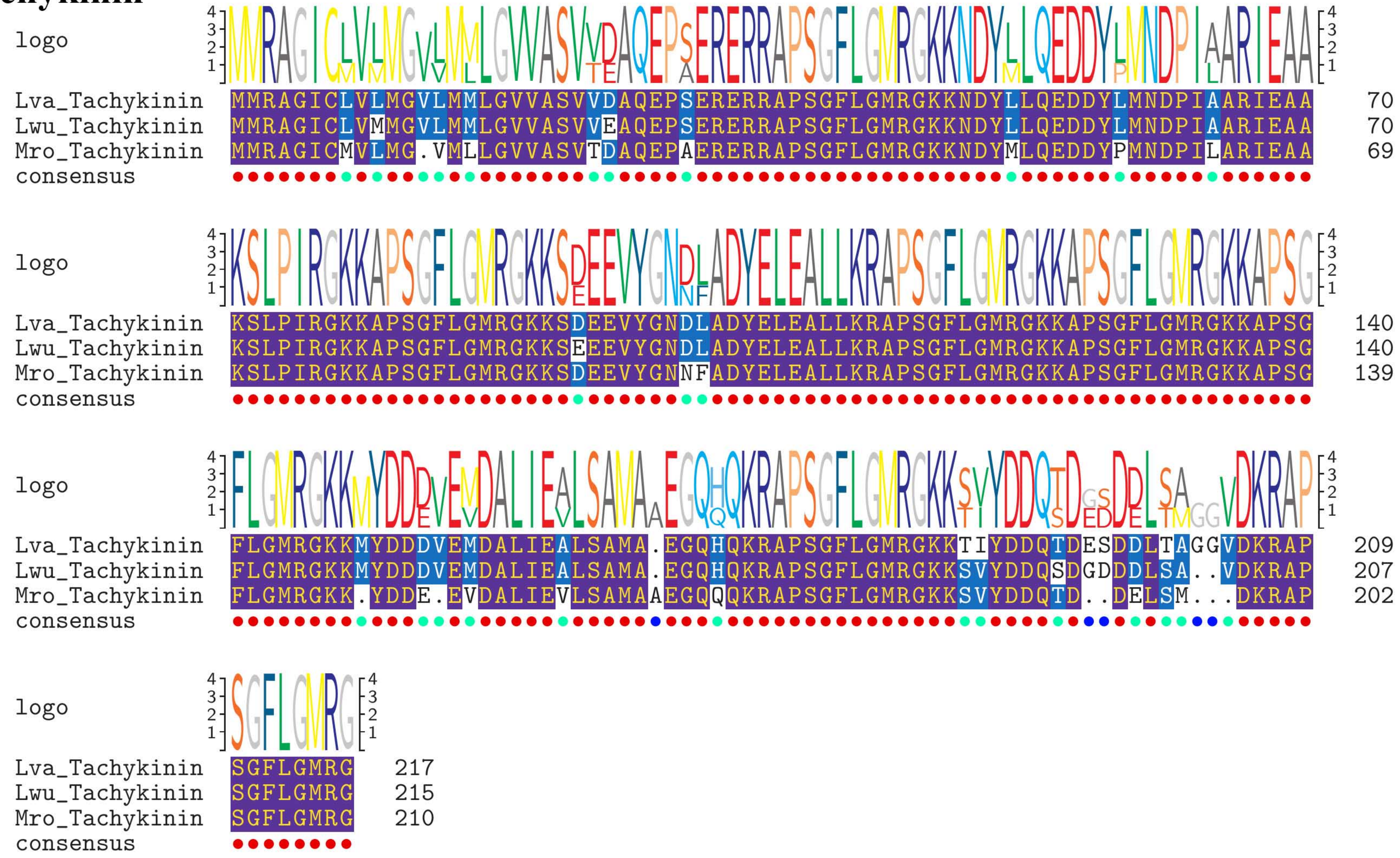

logo
Lva_Tachykinin MMRAGICLVLMGVLMMLGVVASVVDAQEPSERERRAPSGFLGMRGKKNDYLLQEDDYLMNDPIAARIEAA 70
Lwu_Tachykinin MMRAGICLVMMGVLMMLGVVASVVEAQEPSERERRAPSGFLGMRGKKNDYLLQEDDYLMNDPIAARIEAA 70
Mro_Tachykinin MMRAGICMVLMG.VMLLGVVASVTDAQEPAERERRAPSGFLGMRGKKNDYMLQEDDYPMNDPILARIEAA 69
consensus
logo
Lva_Tachykinin KSLPIRGKKAPSGFLGMRGKKSDEEVYGNDLADYELEALLKRAPSGFLGMRGKKAPSGFLGMRGKKAPSG 140
Lwu_Tachykinin KSLPIRGKKAPSGFLGMRGKKSEEEVYGNDLADYELEALLKRAPSGFLGMRGKKAPSGFLGMRGKKAPSG 140
Mro_Tachykinin KSLPIRGKKAPSGFLGMRGKKSDEEVYGNNFADYELEALLKRAPSGFLGMRGKKAPSGFLGMRGKKAPSG 139
consensus
logo
Lva_Tachykinin FLGMRGKKMYDDDVEMDALIEALSAMA.EGQHQKRAPSGFLGMRGKKTIYDDQTDESDDLTAGGVDKRAP 209
Lwu_Tachykinin FLGMRGKKMYDDDVEMDALIEALSAMA.EGQHQKRAPSGFLGMRGKKSVYDDQSDGDDDLSA..VDKRAP 207
Mro_Tachykinin FLGMRGKK.YDDE.EVDALIEVLSAMAAEGQQQKRAPSGFLGMRGKKSVYDDQTD..DELSM...DKRAP 202
consensus
logo
Lva_Tachykinin SGFLGMRG 217
Lwu_Tachykinin SGFLGMRG 215
Mro_Tachykinin SGFLGMRG 210
consensus

TAP

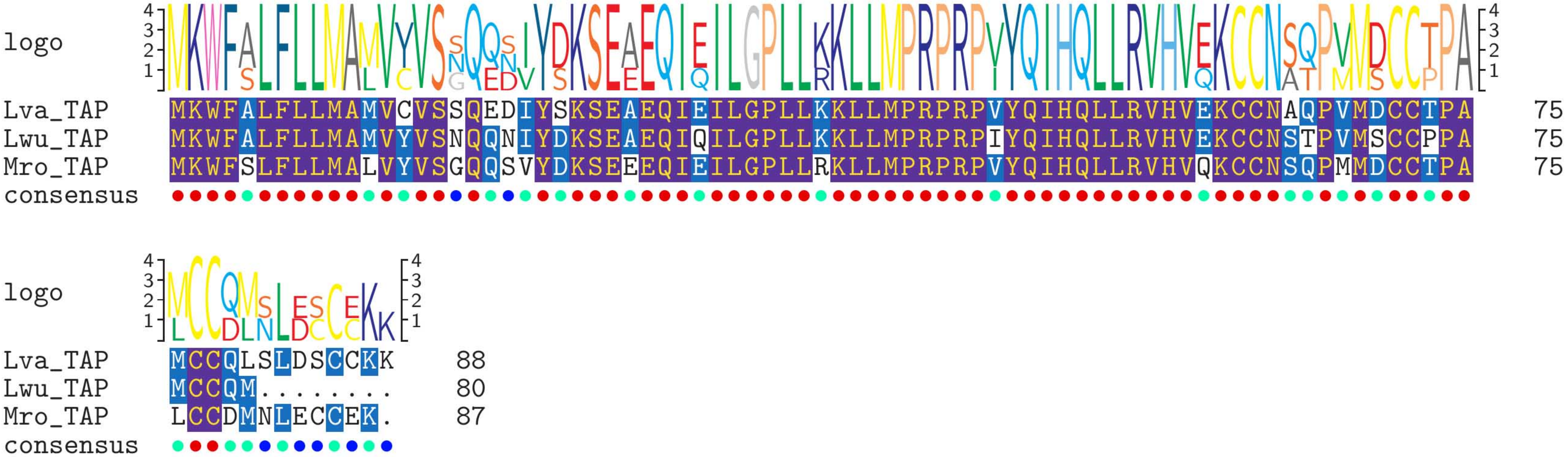
logo
Lva_TAP MKWFALFLLMAMVCVSSQEDIYSKSEAEQIEILGPLLKKLLMPRPRPVYQIHQLLRVHVEKCCNAQPVMDCCTPA 75
Lwu_TAP MKWFALFLLMAMVYVSNQQNIYDKSEAEQIQILGPLLKKLLMPRPRPIYQIHQLLRVHVEKCCNSTPVMSCCPPA 75
Mro_TAP MKWFSLFLLMALVYVSGQQSVYDKSEEEQIEILGPLLRKLLMPRPRPVYQIHQLLRVHVQKCCNSQPMMDCCTPA 75
consensus
logo
Lva_TAP MCCQLSLDSCCKK 88
Lwu_TAP MCCQM........ 80
Mro_TAP LCCDMNLECCEK. 87
consensus

Trissin

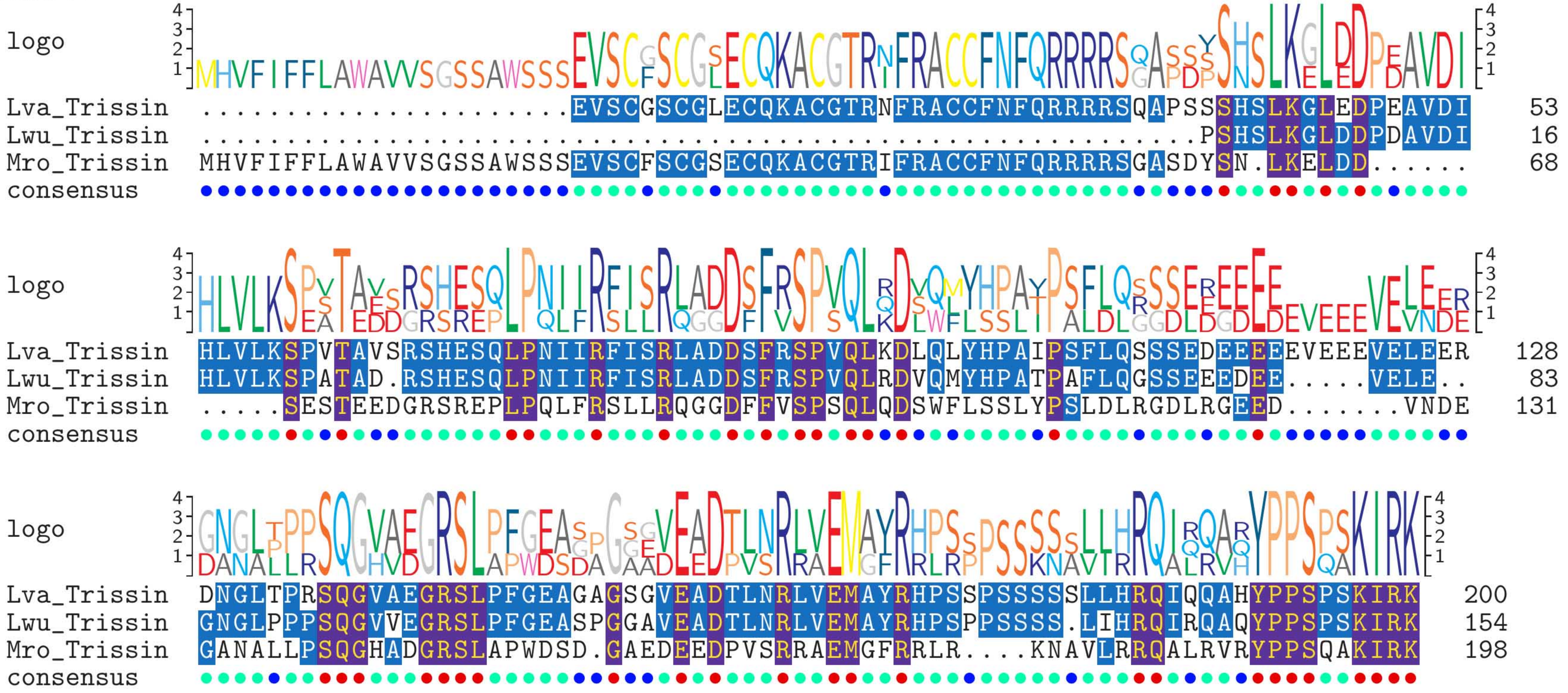
logo
Lva_Trissin ......................EVSCGSCGLECQKACGTRNFRACCFNFQRRRRSQAPSSSHSLKGLEDPEAVDI 53
Lwu_Trissin ....PSHSLKGLDDPDAVDI 16
Mro_Trissin MHVFIFFLAWAVVSGSSAWSSSEVSCFSCGSECQKACGTRIFRACCFNFQRRRRSGASDYSN.LKELDD...... 68
consensus
logo
Lva_Trissin HLVLKSPVTAVSRSHESQLPNIIRFISRLADDSFRSPVQLKDLQLYHPAIPSFLQSSSEDEEEEEVEEEVELEER 128
Lwu_Trissin HLVLKSPATAD.RSHESQLPNIIRFISRLADDSFRSPVQLRDVQMYHPATPAFLQGSSEEEDEE.....VELE.. 83
Mro_Trissin .....SESTEEDGRSREPLPQLFRSLLRQGGDFFVSPSQLQDSWFLSSLYPSLDLRGDLRGEED.......VNDE 131
consensus
logo
Lva_Trissin DNGLTPRSQGVAEGRSLPFGEAGAGSGVEADTLNRLVEMAYRHPSSPSSSSSLLHRQIQQAHYPPSPSKIRK 200
Lwu_Trissin GNGLPPPSQGVVEGRSLPFGEASPGGAVEADTLNRLVEMAYRHPSPPSSSS.LIHRQIRQAQYPPSPSKIRK 154
Mro_Trissin GANALLPSQGHADGRSLAPWDSD.GAEDEEDPVSRRAEMGFRRLR....KNAVLRRQALRVRYPPSQAKIRK 198
consensus

Vasopressin

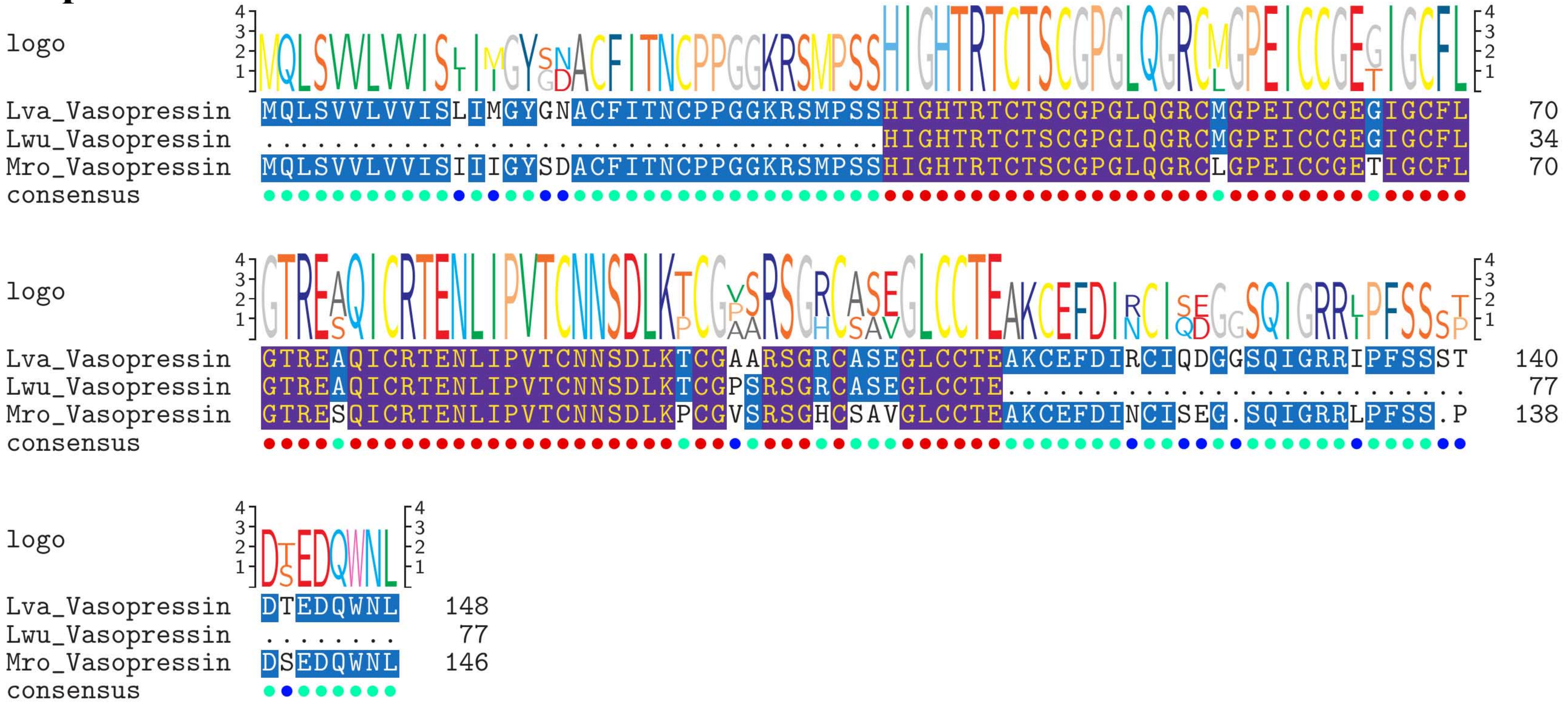

Supplement: Supplementary File 2 — Comparative sequence alignment of peptide precursor in L. vittata with M. rosenbergii and L. wurdemanni. [file Data_Sheet_2.PDF]
